# DNA Base Excision Repair Intermediates Influence Duplex–Quadruplex Equilibrium

**DOI:** 10.3390/molecules28030970

**Published:** 2023-01-18

**Authors:** Mark L. Sowers, James W. Conrad, Bruce Chang-Gu, Ellie Cherryhomes, Linda C. Hackfeld, Lawrence C. Sowers

**Affiliations:** 1 Department of Pharmacology and Toxicology, University of Texas Medical Branch, 301 University Boulevard, Galveston, TX 77555, USA; 2 MD-PhD Combined Degree Program, University of Texas Medical Branch, 301 University Boulevard, Galveston, TX 77555, USA; 3 Department of Internal Medicine, University of Texas Medical Branch, 301 University Boulevard, Galveston, TX 77555, USA

**Keywords:** DNA quadruplex, duplex-quadruplex equilibrium, base excision repair, pyridostatin, glycosylase, telomere

## Abstract

Although genomic DNA is predominantly duplex under physiological conditions, particular sequence motifs can favor the formation of alternative secondary structures, including the G-quadruplex. These structures can exist within gene promoters, telomeric DNA, and regions of the genome frequently found altered in human cancers. DNA is also subject to hydrolytic and oxidative damage, and its local structure can influence the type of damage and its magnitude. Although the repair of endogenous DNA damage by the base excision repair (BER) pathway has been extensively studied in duplex DNA, substantially less is known about repair in non-duplex DNA structures. Therefore, we wanted to better understand the effect of DNA damage and repair on quadruplex structure. We first examined the effect of placing pyrimidine damage products uracil, 5-hydroxymethyluracil, the chemotherapy agent 5-fluorouracil, and an abasic site into the loop region of a 22-base telomeric repeat sequence known to form a G-quadruplex. Quadruplex formation was unaffected by these analogs. However, the activity of the BER enzymes were negatively impacted. Uracil DNA glycosylase (UDG) and single-strand selective monofunctional uracil DNA glycosylase (SMUG1) were inhibited, and apurinic/apyrimidinic endonuclease 1 (APE1) activity was completely blocked. Interestingly, when we performed studies placing DNA repair intermediates into the strand opposite the quadruplex, we found that they destabilized the duplex and promoted quadruplex formation. We propose that while duplex is the preferred configuration, there is kinetic conversion between duplex and quadruplex. This is supported by our studies using a quadruplex stabilizing molecule, pyridostatin, that is able to promote quadruplex formation starting from duplex DNA. Our results suggest how DNA damage and repair intermediates can alter duplex-quadruplex equilibrium.

## 1. Introduction 

DNA constantly undergoes persistent damage and repair. If this damage goes unrepaired, it can result in genomic instability and mutations that predispose towards the development of cancer. The primary DNA repair pathway for repairing endogenous deamination and oxidative damage is the base excision repair (BER) pathway [1,2]. Multiple groups have made substantial efforts to explore mechanisms of DNA repair. However, most of these studies have been conducted with canonical duplex DNA and not with the unusual secondary structures also found in our genome. 

Substantially less is known about non-canonical DNA secondary structures and their potential roles. In DNA, specific sequence motifs are known to adopt non-duplex structures, including Z-DNA, triplexes, quadruplexes, hairpins, and cruciforms [3,4]. Deviating from normal Watson–Crick base pairing, and forming Hoogsteen pairing, protonated bases, or some combination of the two, allows for the formation of various alternative secondary structures. Sequence motifs known to form these different structures are found throughout the genome, including promoter regions and the telomeric ends of chromosomes. The biological functions of unusual DNA structures are incompletely understood but are thought to be involved in cellular processes, including DNA replication, recombination, and gene expression [5].

Among the possible non-canonical structures, the G-quadruplex has been studied in the greatest detail. Some investigators have estimated that there may be as many as ~700,000 quadruplex-forming regions in the human genome [6]. Many are present in the promoter regions of oncogenes and in telomeric repeats that are aberrantly extended in most human cancers. Under appropriate salt conditions, quadruplexes can form from DNA sequences containing four or more runs of three guanines (G), each separated by a few nucleotides (loops). Notably, various quadruplex configurations have been reported depending on the sequence and type of cationic salt conditions, including antiparallel, parallel, hybrid, and others. Furthermore, at different time scales, interconversions between different possible configurations can also occur [7,8,9]. Consequently, quadruplex structures can be dynamic and undergo folding, unfolding, and adopt different configurations. 

How DNA damage might influence quadruplex structure, or how its unique structure impacts BER is complex. The formation of alternative secondary structures, including the quadruplex, potentially interfering with DNA repair has been discussed previously [3,10,11]. Importantly, nearly all studies of DNA damage in quadruplex DNA have only focused on the impacts of oxidative damage to guanine [12,13,14,15,16,17]. Guanine is typically directly involved in the secondary structure of the quadruplex. Depending on the position of the oxidized guanine, quadruplex folding can be influenced [14,16,17]. Additionally, the type of guanine oxidation product, its position, and which DNA glycosylase is present, can all influence BER in quadruplex DNA. 

**Figure 1:**
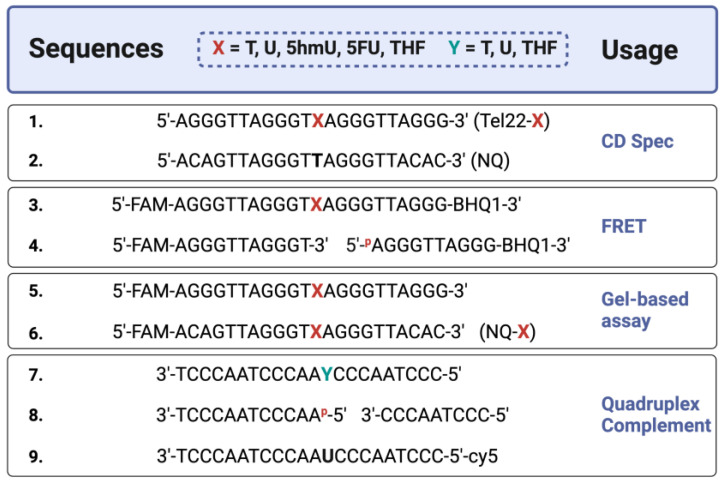
Sequences of oligonucleotides used in this study. Synthetic oligonucleotides of the telomere repeat sequence (Tel22), known to form a quadruplex structure, were prepared with various modifications. CD studies were performed on oligos without a fluorophore or quencher [Oligos 1–2]. FRET studies used a 6-carboxyfluorescein (FAM) and a 3′-non-fluorescent quencher (BHQ1) [Oligos 3–4], containing thymine (T) or its analogs uracil (U), 5-hydroxymethyluracil (5hmU), 5-fluorouracil (5FU), and a stable synthetic abasic site (THF). For gel-based studies, FAM-only oligos were used [Oligos 5–6]. A non-quadruplex (NQ) sequence of the same length [Oligos 2 and 6] was also prepared. In certain experiments, the quadruplex strand was annealed to a complementary strand [Oligos 7–9] to form a duplex where ‘Y’ is either T, U, THF, or two shorter oligos that simulate a one-base gap. Created with Biorender.com.

These previous studies have revealed how some DNA glycosylases can or cannot act on guanine oxidation damage products but have overlooked two additional aspects of DNA damage and repair in quadruplexes. Firstly, it is valuable to examine DNA damage other than guanine oxidation. DNA bases other than guanine can also undergo endogenous DNA damage, including hydrolytic deamination and oxidation [18,19,20]. In order to investigate the role of pyrimidine DNA damage on quadruplex formation and subsequent repair, we inserted uracil (U), 5-hydroxymethyluracil (5hmU), and 5-fluorouracil (5FU) into the loop regions, in the place of thymine (T), of the human telomere sequence A(GGGTTA)_3_GGG (Tel22) [21]. The pyrimidine analogs examined herein represent hydrolytic deamination of cytosine (C) to U, oxidation of T to 5hmU, and the incorporation of the chemotherapy agent 5FU into DNA (Figure 1). 

The second unexplored factor is that previous investigations of DNA quadruplexes have been conducted primarily with the G-rich strand alone. While this ensures quadruplex formation, under most circumstances the complementary strand is present in vivo. When the complementary strand is present, an equilibrium is established between the duplex and quadruplex, with the duplex being the predominate configuration [22,23,24]. To date, few studies have examined how DNA damage and repair might influence the equilibrium that exists between duplex and quadruplex structures.

To address these two key factors, we first examined the influence of these analogs on the capacity of Tel22 to form a quadruplex structure using circular dichroism (CD) and fluorescence resonance energy transfer (FRET). We then probed the quadruplex structures with glycosylases and an AP endonuclease, which comprise the first two steps of BER, to determine how such structures influence DNA repair. Finally, we examined how the BER intermediates alter duplex–quadruplex equilibrium. 

## 2. Results

A series of oligonucleotides were synthesized for this study and the sequences and modifications are shown in Figure 1. Non-fluorescent quadruplex-forming oligonucleotides were prepared for CD spectroscopy studies as well as complementary oligonucleotides to form a duplex with the Tel22 quadruplex sequence. We used 5′-6-carboxyfluorescein (FAM) fluorophore-labeled oligonucleotides for gel-based studies. For FRET studies, we added a non-fluorescent quencher, 3′-BHQ1, in addition to the 5′-FAM fluorophore. 

### 2.1. Pyrimidine Analogs in Loops Do Not Affect Quadruplex Formation

**Figure 2:**
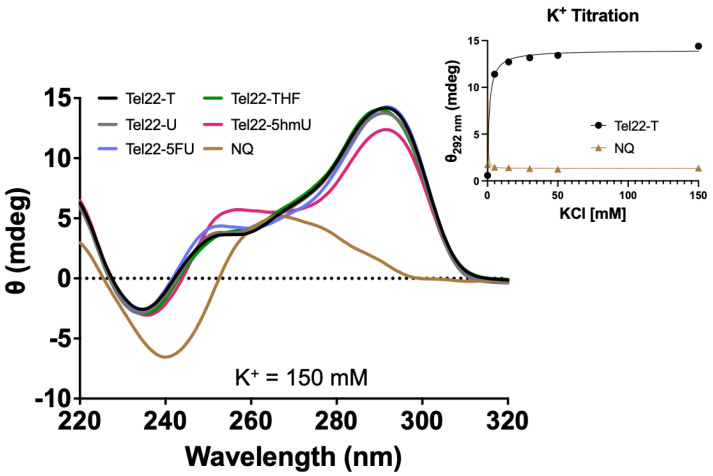
Pyrimidine analogs in the loop region did not disrupt G-quadruplex formation. CD spectra were acquired for quadruplex Tel22-X oligonucleotides or a non-quadruplex-forming (NQ) oligonucleotide. A 4 μM solution was prepared in 20 mM Tris buffer, pH 7.4, 150 mM KCl, and 15 mM NaCl and CD spectra were obtained from 320 to 220 nm at 37 °C. The spectra here were consistent with the formation of a hybrid-type G-quadruplex [21]. The 5hmU-containing oligonucleotide appeared slightly different from the others likely due to both intra- and intermolecular hydrogen bonding of 5hmU and N7 of adjacent guanines [25]. Tel22-T and the NQ were titrated from 0 to 150 mM KCl in a 20 mM Tris buffer at pH 7.4. Using a one-site specific binding model in the PRISM software, we estimated K_d_ of 1.2 ± 0.2 mM for quadruplex binding while no changes in CD spectra were seen with the NQ oligonucleotide. Titration experiments were done in triplicate, error bars of S.D. were smaller than the data points. Oligonucleotides were equilibrated in buffer at RT for a minimum of 30 min.

CD spectra were acquired for oligonucleotides containing the wild-type 22-mer telomeric repeat sequence (Tel22-T) or those containing a U, 5hmU, 5FU, or a stable abasic site, THF, (Tel22-X) as shown in Figure 1. Unless otherwise indicated, a physiologic-like buffer containing 150 mM KCl and 15 mM NaCl in 20 mM Tris buffer at pH 7.4 was used, and data were acquired at 37 °C (Figure 2). As a negative control, we used a non-quadruplex (NQ) forming 22-mer (Figure 1). Upon quadruplex formation, a positive band was seen near 295 nm, in accordance with a previous study demonstrating this telomeric repeat sequence forms a hybrid type quadruplex in the presence of K^+^ [21]. Spectra of the quadruplexes substituted with U, 5hmU, 5FU, and a tetrahydrofuran (THF) abasic site were nearly indistinguishable. The quadruplex containing a 5hmU substitution (red) showed a lower band intensity at 295 nm and increased band intensity at 250 nm. This could potentially be attributed to both intra- and intermolecular hydrogen bonding of 5hmU with the N7 of an adjacent guanine [25].

The CD spectra confirmed the formation of a quadruplex and that pyrimidine substitutions in the central loop did not interfere or prevent quadruplex formation. Quadruplex formation is highly dependent upon the environmental conditions. In particular, the presence of Na^+^ or K^+^ can greatly influence quadruplex formation and the type of quadruplex structure. We obtained the CD spectrum of the Tel22-T oligonucleotide as a function of KCl concentration (Figure 2). The approximate K_d_ was 1.2 ± 0.2 mM and complete quadruplex formation was seen by 30 mM KCl while the NQ sequence showed no peak at 295 nm or any changes as a function of K^+^.

### 2.2. The Activity of DNA Glycosylases and AP Endonuclease 1 (APE1) Are Impaired by Quadruplex Structure 

**Figure 3:**
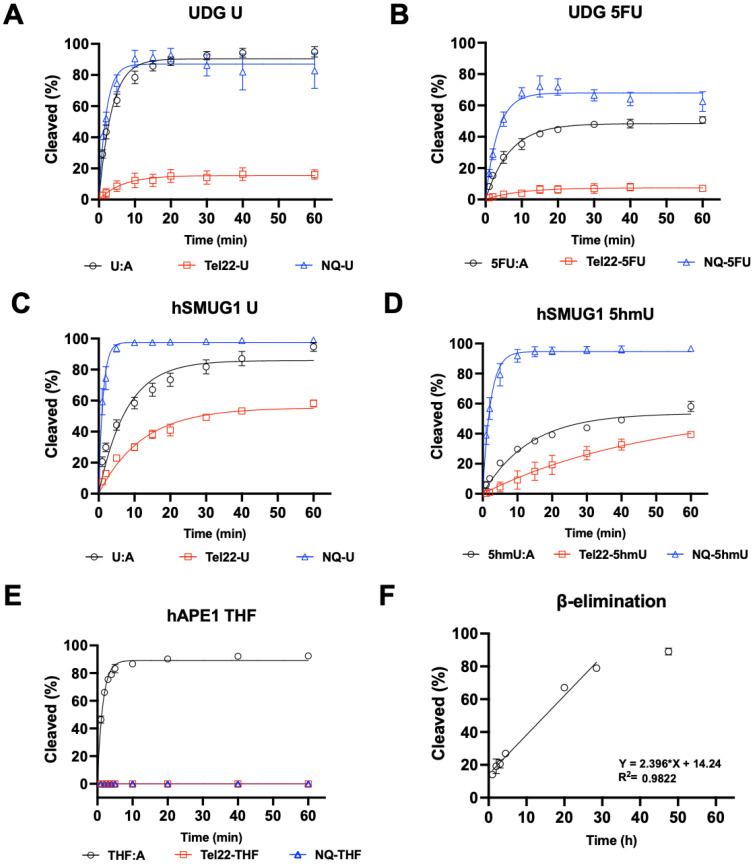
Quadruplex inhibited DNA glycosylase and APE1 activity. UDG, hSMUG1, and APE1 activity on quadruplex oligonucleotides (Tel22-X) containing U, 5hmU, 5FU, or THF was compared to duplex, or single-stranded DNA using the NQ-X oligonucleotides. (**A**) UDG (0.613 nM, 300:1 DNA to enzyme ratio) was fastest on single-stranded U (NQ-U) and duplex (U:A) followed by quadruplex (Tel22-U). (**B**) UDG (6.13 nM, 30:1 DNA to enzyme ratio) was overall less efficient on 5FU but the trend was the same, NQ-5FU > 5FU:A > Tel22-5FU. (**C**,**D**) hSMUG1 (6.2 nM, 30:1 DNA:enzyme ratio) followed a similar trend with either U or 5hmU. (**E**) Under the conditions tested, APE1 (1.31 nM, 140:1 DNA:enzyme ratio) was only active on duplex (THF:A) and no activity on Tel22-THF or NQ-THF was observed. (**F**) Because of the poor activity of APE1 on THF in a quadruplex, we wanted to estimate the rate of spontaneous β-elimination. We generated an abasic site in situ by using an excess of UDG to remove U from Tel22-U containing oligonucleotide. We then continued incubating the quadruplex containing abasic site for up to 50 h at 37 °C and measured spontaneous β-elimination over time using gel electrophoresis. The rate of elimination was determined by a linear fit to be 0.024 h^−1^ with a half-life of 21 h. Gels scans are shown in Figures S1–S16 Supplementary material.

Because the pyrimidine modifications U, 5hmU, and 5FU did not inhibit quadruplex formation, we then sought to understand how these structures would be substrates for DNA repair enzymes. In the studies shown in Figure 3, the targets for repair were placed into the quadruplex-forming strand and incubated with either DNA glycosylases or APE1, and enzymatic activity was quantified by gel [26]. When the quadruplex-forming oligonucleotide containing a U was paired with a complementary strand (U:A), the U was rapidly excised by UDG at a rate similar to the repair of U from a non-quad-forming single strand. However, when in a quadruplex, U excision by UDG was significantly inhibited (Figure 3A). UDG can also excise 5FU (Figure 3B), albeit more slowly. As with U repair, removal of 5FU from a quadruplex was slower than in duplex or single-stranded DNA. 

We then examined hSMUG1 activity under similar conditions but reduced the K^+^ concentration from 150 mM to 50 mM for optimal enzymatic activity [27]. hSMUG1 can excise U and 5hmU (Figure 3C,D). The trend in repair rates for U and for 5hmU was single-strand > duplex > quadruplex. hSMUG1 was not as inhibited as UDG by the quadruplex structure. Our results are in accord with previous studies that showed quadruplex formation inhibiting glycosylase excision [13,15,28].

Glycosylase excision of a target base is the initiating event for BER. The resulting abasic site is then cleaved by a lyase or AP endonuclease. In this experiment, an abasic site was simulated with the THF-abasic site. Substrates were incubated with repair enzymes in buffer at 37 °C, and the resulting fluorescently tagged oligonucleotides were separated by gel electrophoresis and quantified. When in a duplex (Figure 3E), the abasic site was cleaved by APE1. However, when an abasic site was in a quadruplex or single-stranded context, APE1 could not cleave its substrate or was greatly inhibited. This is consistent with previous reports of APE1 inhibition by quadruplex formation [29,30].

The inability of APE1 to cleave an abasic site when not in a duplex would halt the BER pathway. Furthermore, abasic sites are labile to attack by water resulting in β-elimination and strand cleavage, which could be cytotoxic. Here, we generated an abasic site in a U-containing quadruplex with UDG and then incubated the oligonucleotide at 37 °C for several days. Aliquots were taken as a function of time, and oligonucleotides were separated by gel electrophoresis. The apparent half-life for the cleavage of an abasic site in a quadruplex structure was approximately 21 h (Figure 3F). Previously, the rate of abasic site cleavage in duplex DNA was reported to be 190 h at pH 7.4 at 37 °C in a buffer containing Mg^2+^ [31].

### 2.3. Quadruplex–Duplex Equilibrium Can Be Monitored by FRET

**Figure 4:**
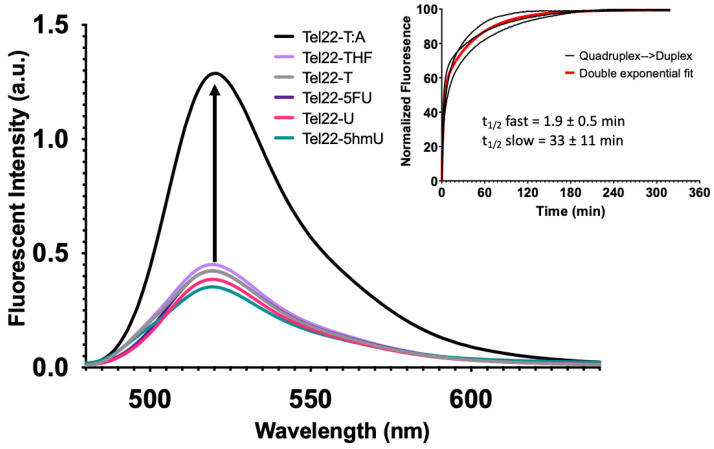
G-quadruplex formation quenched fluorescence and the addition of a complementary strand promoted duplex formation. While quadruplexes are highly stable secondary DNA structures, in the presence of the complementary strand, they are in equilibrium with the corresponding duplex. A FRET strategy was employed to monitor quadruplex formation at 37 °C. Relatively low fluorescent excitation was observed when oligonucleotides formed a G-quadruplex as the fluorophore (FAM) and quencher (BHQ1) were in close contact. However, fluorescence increased when the quadruplex-containing sequence was annealed to its complementary strand to form a duplex (Inset). We then performed a time course experiment to examine the kinetics of quadruplex unfolding as it transitioned to a duplex. Black curves represent three independent experiments and the exponential fit in red. A single exponential fit poorly; therefore, we applied a double-exponential fit, which suggested that there was a relatively fast phase of quadruplex unfolding to one or more intermediate structures, followed by a slower complete unfolding to duplex. The first half-life was 1.9 ± 0.5 min and the second was 33 ± 11 min. The equation for the curve was $y=A_{1}\left(1-e^{-k_{1}t}\right)+A_{2}\left(1-e^{-k_{2}t}\right)$ where *k*_1_ and *k*_2_ are 0.37 min^−1^ and 0.02 min^−1^, respectively. The first and second amplitudes were estimated to be 54.5 and 99.3, respectively.

Quadruplex formation can also be monitored by FRET as described previously [32]. In this system, a fluorophore (FAM) was placed on the 5′-end of the oligonucleotide, and a non-fluorescent quencher (BHQ1) was placed at the 3′-end. When paired with a complementary sequence, the quadruplex-forming strand formed a duplex, separating the fluorophore and quencher. This yielded a maximum fluorescence intensity as seen for the wild-type Tel22 sequence (Figure 4, Tel22-T:A). However, in the absence of the complementary strand, the Tel22 sequence may fold into a quadruplex structure. This brings the fluorophore and quencher in proximity, resulting in a minimum fluorescent intensity (Figure 4). Neither the pyrimidine analogs nor the abasic site (Tel22-THF) oligonucleotides altered quadruplex formation. Therefore, quadruplex formation was not affected by these modifications when placed in the loop regions.

The overlaid fluorescence spectra of the Tel22-X sequences alone and annealed to its complement (i.e., duplex) are shown in Figure S17, respectively. The presence of the complementary strand mostly inhibited quadruplex formation regardless of the pyrimidine analog. We also examined how a gap in the Tel22 sequence (Figure 1, Oligo 4) would affect quadruplex formation as the presence of an abasic site BER intermediate surprisingly did not inhibit quadruplex formation (Figure 2 and Figure 4, Tel22-THF). However, formation of a gap generates two fragments of the quadruplex where the FAM and BHQ1 ends are now separate strands. As expected, the quadruplex fragments (Oligo 4) abolished quadruplex formation and a similar fluorescence intensity to the duplex oligonucleotides was observed (Quadruplex Fragments, Figure S17).

Upon addition of the complementary strand, unwinding of the quadruplex with formation of a duplex occurred with all systems studied here; however, the transition was not instantaneous. Fluorescence intensity as a function of time was monitored with a qPCR instrument. Experimental fluorescence intensity from the Tel22-T as it unfolded and formed a duplex with its complement (T:A) is shown in the inset of Figure 4. The data from the three separate reactions were best fit by a double exponential equation as shown (red). The initial half-life was 1.9 ± 0.5 min while the second half-life was 33 ± 11 min. We propose that the quadruplex and complement initially bind at one end in a fast step and the remaining quadruplex is then unzipped, forming the duplex in a slow step. Our study is in accord with previous studies, which show that quadruplex–duplex interconversion can be slow and may occur through one or more stable intermediates [33,34]. The significance of this finding is that the kinetics of interconversion might become important when trying to interpret the biological consequences of quadruplex-forming sequences [35]. 

### 2.4. Base Excision Repair and Quadruplex Stabilizing Ligands Shift the Duplex–Quadruplex Equilibrium towards Quadruplex

**Figure 5:**
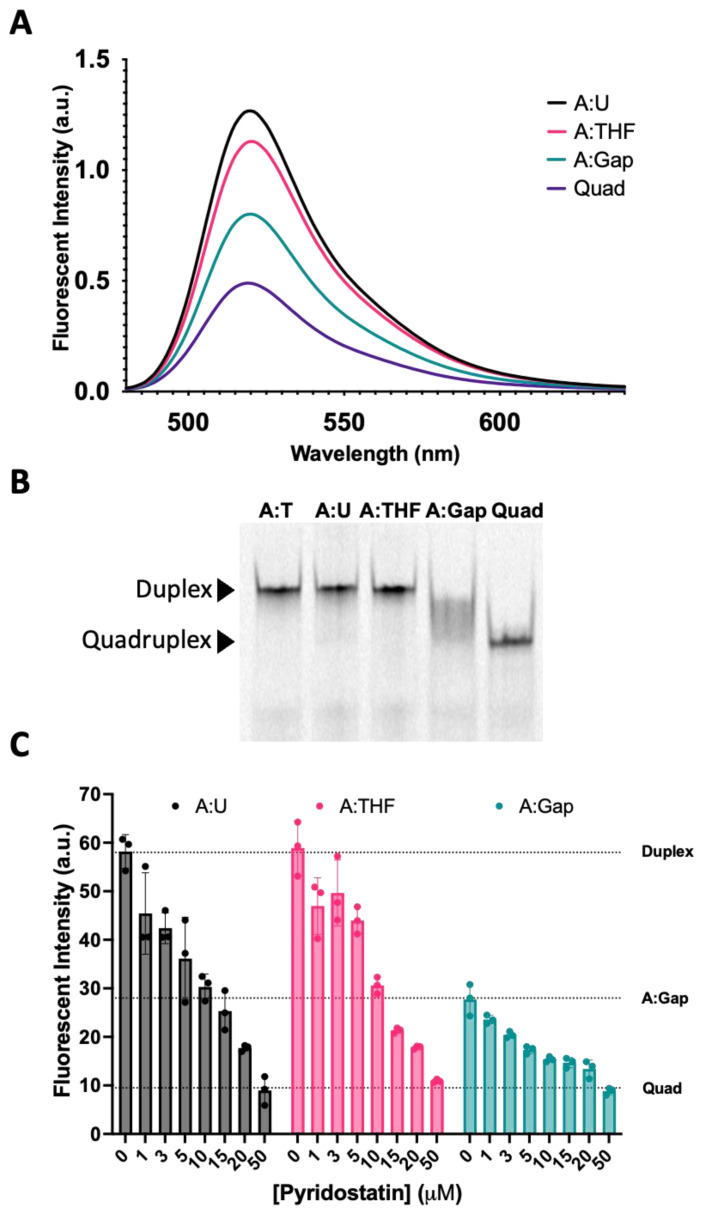
Reconstitution of repair intermediates promoted quadruplex formation. (**A**) Annealing Tel22-T with a complementary strand containing U (A:U) promoted duplex formation as indicated by an increase in fluorescence relative to the quadruplex-only control (Quad). When a complementary strand containing a stable abasic site (A:THF) was used instead, fluorescence was reduced relative to the A:U duplex, suggesting the destabilization of the duplex and promoting quadruplex. Interestingly, generating a gap in the complementary strand (A:Gap) further destabilized the duplex and shifted the equilibrium towards quadruplex. (**B**) Using a 20% native polyacrylamide gel, we demonstrated that A:U and A:THF were duplex as they migrated the same as the A:T control (Lanes 1–3). On the other hand, a gapped complement (A:Gap) showed some intermediate between duplex and quadruplex (Lane 4). Lane 5 was the quadruplex-only control. (**C**) We saw a concentration-dependent decrease in fluorescence when we titrated oligonucleotides that could form a duplex with a quadruplex-stabilizing small molecule, pyridostatin. Consistent with A and B, the A:Gap oligonucleotide had ~50% of the fluorescence as either A:U or A:THF. Oligonucleotides were prepared as a 1 μM solution in 20 mM Tris, pH 7.4, 150 mM KCl, and 15 mM NaCl and equilibrated in buffer for a minimum of 30 min. 1.2 equivalents of the corresponding complementary strands were annealed at 90 °C for 5 min and cooled at RT. Gel samples were prepared identically. Pyridostatin was allowed to equilibrate with oligonucleotides for 30 min at 37 °C and fluorescence emission of FAM was acquired using a qPCR instrument.

We then examined the fluorescence intensity of oligonucleotide complexes that recapitulate the steps in BER (Figure 5A). The fluorescence spectra for the Tel22-T quadruplex annealed to a complement containing uracil (A:U) were nearly identical to the wild-type duplex from Figure 4 (Tel22-T:A). When the U in the complementary strand was replaced with a THF abasic site, the fluorescence intensity at equilibrium decreased (magenta), reflecting a shift in the duplex–quadruplex equilibrium toward the quadruplex. A one-base gap was simulated by adding two oligonucleotides complementary to the 5′-side and the 3′-side of the quadruplex-forming sequence (Figure 1, Oligo 8). The fluorescence intensity dropped further (green) to a level approximately midway between the duplex and quadruplex. We sought to further examine the duplex–quadruplex equilibrium by non-denaturing gel electrophoresis (Figure 5B). The fluorescently labeled A:T, A:U, and A:THF duplexes were observed as a single band with the same relative gel migration. The quadruplex, in the absence of a complementary strand, was observed as a single band with lower gel migration. However, the quadruplex sequence with a one-base gap in the complementary strand did not show resolution towards either a single-stranded quadruplex or duplex (Figure 5B). The fluorescence data as well as the gel data suggest this system is best described as an ensemble of interconverting structures, including both duplex and quadruplex configurations, in approximately equal proportions. 

To further explore this hypothesis, we used a quadruplex-stabilizing small molecule, pyridostatin, to examine if the equilibrium could be shifted from duplex towards quadruplex. We titrated A:U, A:THF, and A:Gap oligonucleotides with increasing amounts of pyridostatin and monitored changes in fluorescence intensity using a qPCR instrument (Figure 5C). In the absence of pyridostatin, the A:U and A:THF oligonucleotides had relatively high fluorescence while the A:Gap oligonucleotide had roughly ~50% of the fluorescence intensity. Surprisingly, 5–10 µM of pyridostatin was sufficient to drop the fluorescent intensity to that of the A:Gap oligonucleotide. With all the oligonucleotides examined, increasing amounts of pyridostatin further quenched the fluorescence. This suggests that duplex and quadruplex may interconvert and pyridostatin traps the oligonucleotide in the quadruplex state. In all cases, 50 µM of pyridostatin drove all complexes to quadruplex. These results were consistent with previous reports that demonstrated a small molecule could promote quadruplex formation in the presence of its complementary C-rich strand [36].

**Figure 6:**
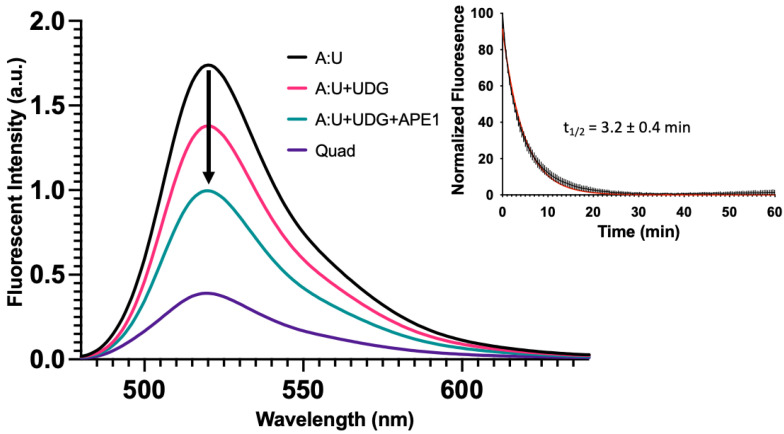
Base excision repair promotes quadruplex formation. In Figure 5, we demonstrated with synthetic oligonucleotides that the presence of a complementary strand containing an abasic site or gap destabilized the duplex and promoted quadruplex formation. Here, we simulated the same process but enzymatically prepared the abasic site and gapped DNA duplex using UDG or UDG and APE1, respectively. The fluorescent emission spectrum was taken for the quadruplex-only strand, duplex oligonucleotide containing a U in the C-rich strand (A:U), duplex [1 µM] incubated with 10 U of UDG (3.39 pmol, 34 nM), and duplex incubated with 10 U of UDG (3.39 pmol, 34 nM) and 20 U of APE1 (0.71 pmol, 7.1 nM) for 2 h at 37 °C in 100 µL total volume. Following UDG treatment, fluorescence decreased ~25% followed by a ~50% decrease in fluorescence with UDG and APE1. This suggested that the equilibrium between duplex and quadruplex could shift following DNA repair. We then wanted to estimate the time scale for secondary structure changes in the quadruplex strand, as a gap formed in the opposing strand. The duplex A:U containing oligonucleotide [1 µM] was treated with 1.25 U of UDG (0.42 pmol, 16.8 nM) and 2.5 U of APE1 (0.09 pmol, 3.6 nM) and fluorescence was monitored over time at 37 °C in a qPCR instrument in 25 µL total volume. As a gap was introduced into the opposing strand, fluorescence of the quadruplex strand decreased with time. This suggested that as the gap was introduced, the quadruplex-forming strand underwent relatively fast changes in configuration that brought the FAM and BHQ1 quencher closer together. The half-life was estimated to be 3.2 ± 0.4 min using a single exponential decay (Inset). This contrasted with the much slower unfolding of the quadruplex (Figure 4, Inset). The black curve represents the average of three replicates with the vertical lines as the S.D. The red curve shows a single exponential fit. In an identical experiment where the complementary oligonucleotide containing U was labeled with Cy5, we monitored the gap formation by gel (Figure S18).

In the study shown in Figure 5, repair intermediates were generated by combining various oligonucleotides and allowing them to form duplexes prior to the fluorescence measurements. Having examined the fluorescence intensity changes associated with simulated BER intermediates, we sought to determine if similar results would be obtained in a reconstituted DNA repair system (Figure 6). In this study, we measured the fluorescence spectra of the same A:U duplex (Figure 6, black). Upon incubation with uracil DNA glycosylase (UDG), an abasic site was formed and fluorescence dropped (Figure 6, magenta). The simultaneous addition of UDG and APE1 generated a repair gap with a further drop in fluorescence (Figure 6, green). These data revealed that the fluorescence changes for the simulated and enzyme-generated intermediates were essentially the same, and therefore measuring fluorescence could be used to monitor enzymatic DNA repair reactions. Our earlier studies showed that some time was required for these systems to reach equilibrium (Figure 4, Inset). We therefore measured the change in fluorescence intensity of this system as a function of time following the addition of UDG and APE1. Data from three independent experiments were averaged (Figure 6, Inset). The apparent half-life for reaching equilibrium was approximately 3.2 ± 0.4 min. It appears that as a gap was formed in the complementary strand (Figure S18), simultaneously the quadruplex-forming strand underwent changes in its configuration such that it brought the 5′ and 3′ ends closer into contact. Regardless, the apparent rate at which a quadruplex-like structure began to form from a damaged duplex was substantially faster than the rate of quadruplex unfolding during duplex formation (Figure 4, Inset). 

Previous studies on the biological consequences of quadruplex-forming sequences have examined the systems once equilibrium has been achieved. Our results suggest that achieving structural equilibrium in such systems may be slower than biochemical reactions including base excision and AP endonuclease cleavage. Therefore, inferring biological consequences of quadruplex structures when at equilibrium might need to be reconsidered when such structures are generated as part of a biochemical pathway.

## 3. Discussion 

### 3.1. DNA Damage and Repair in Quadruplex DNA

The DNA of all organisms is persistently damaged by endogenous reactive molecules as well as exogenous agents [18]. Most of the single-base lesions can be repaired by the BER pathway. Previous BER studies have focused primarily upon the repair of normal duplex DNA and not quadruplex structures, which can affect DNA repair. Furthermore, the studies that have examined base lesions and their repair in DNA quadruplexes have primarily only examined the oxidative damage and repair of guanine adducts [12,13,14,15,16,17]. In addition to DNA damage of guanine, other forms of DNA damage can and do occur, such as hydrolytic deamination of C to U, oxidation of T to 5hmU, abasic sites, and strand breaks. These forms of DNA damage and their repair have been understudied in the context of DNA quadruplexes. 

Pyrimidine analogs can also be misincorporated by DNA polymerases, in place of T, under physiological conditions. This is exploited by chemotherapy drugs including methotrexate, which impairs folate metabolism. This compromises thymidylate synthase resulting in increased formation of dUTP, which can compete with dTTP during DNA replication, placing U into DNA [18,37]. Similarly, pyrimidine analogs 5hmU and 5FU can be converted to the corresponding 5′-triphosphates and compete for incorporation into DNA during replication [38,39,40,41,42,43]. The cytotoxicity resulting from the presence of U, 5hmU, and 5FU incorporation into DNA is incompletely understood. If unrepaired, the presence of these analogs can interfere with DNA–protein interactions. Furthermore, the initiation of the BER pathway can be cytotoxic when the total number of strand breaks exceeds the threshold for repair capacity [37,38,39,41]. 

Pyrimidine analogs in the loops of quadruplexes did not disrupt folding. U, 5hmU, and 5FU did not inhibit quadruplex formation by either CD spectroscopy or FRET-based techniques (Figure 2 and Figure 4). Similarly U and likely 5FU and 5hmU, when located in the C-rich complementary strand of a corresponding duplex, did not substantially diminish duplex stability or alter the equilibrium between duplex and quadruplex configurations (Figure 5 and Figure 6). Surprisingly, the introduction of an abasic site, mimicking a BER intermediate, into the quadruplex sequence did not inhibit its formation (Figure 2 and Figure 4). However, if a single-base gap was introduced into the loop region, generating quadruplex fragments (Figure 1, Oligo 4), this abolished quadruplex formation (Figure S17, Quadruplex Fragments). 

While these pyrimidine analogs did not inhibit quadruplex formation, their repair was significantly hampered. The three analogs studied here (U, 5hmU, and 5FU) are substrates for the monofunctional glycosylases UDG and SMUG1 when located in duplex structures but are excised much more slowly when in the loop regions of quadruplex structures (Figure 3). Importantly, APE1 was unable to cleave an abasic site when it was present in either single-stranded or quadruplex DNA. These results are in accord with previous studies [10,28,29,30]. Abasic sites are unable to be repaired by APE1 and could undergo spontaneous β-elimination, generating a strand break. We estimated the apparent half-life for spontaneous β-elimination to be 21 h, which was significantly faster than the rate previously reported for duplex DNA, 190 h [31]. 

The challenges with repairing these analogs in quadruplexes could represent a therapeutic approach for cancer treatment. For example, incorporation of some pyrimidine analogs, and their subsequent repair by DNA glycosylases, in quadruplexes could be poorly repaired and potentially lethal. If the glycosylases can remove the analogs, this would result in an abasic site. Because of the very poor APE1 activity on quadruplex forming DNA, this would result in spontaneous β-elimination of the abasic site and formation of a single-strand break. Closely spaced single-strand breaks could result in potentially lethal double-strand breaks [44].

### 3.2. Base Excision Repair Promotes Quadruplex Formation

Most quadruplex-forming sequences are in equilibrium with duplex DNA. Although the thermal stability of a quadruplex is similar to that of the corresponding duplex, the duplex is the preferred configuration under physiological conditions of salt concentration and temperature [22,23,24]. Given sufficient time, we observed that the quadruplex converted to duplex at physiologic conditions (Figure 4, Inset). The addition of a single strand complement to a preformed quadruplex could unwind the quadruplex with formation of a duplex that had a fast and slow half-life of approximately 2 and 33 min, respectively. These results were similar in timescale to quadruplex unfolding seen previously [34]. 

Duplex–quadruplex equilibrium can be shunted towards quadruplex by destabilizing the duplex. The BER intermediates, introduced either synthetically or enzymatically in the complementary strand of a quadruplex sequence, altered the duplex–quadruplex equilibrium (Figure 5 and Figure 6). DNA glycosylase mediated excision of an analog from the strand complementary to the quadruplex strand, shifted the equilibrium slightly toward the quadruplex (Figure 6). Cleavage of the resulting abasic site with APE1, generating a one-base gap, resulted in roughly equal populations of duplex and quadruplex configurations. Using a FRET approach, we observed that the formation of a quadruplex from a duplex was not instantaneous and proceeded with a half-life of approximately 3 min (Figure 6, Inset). 

### 3.3. Duplex to Quadruplex Transition Using Pyridostatin

**Figure 7:**
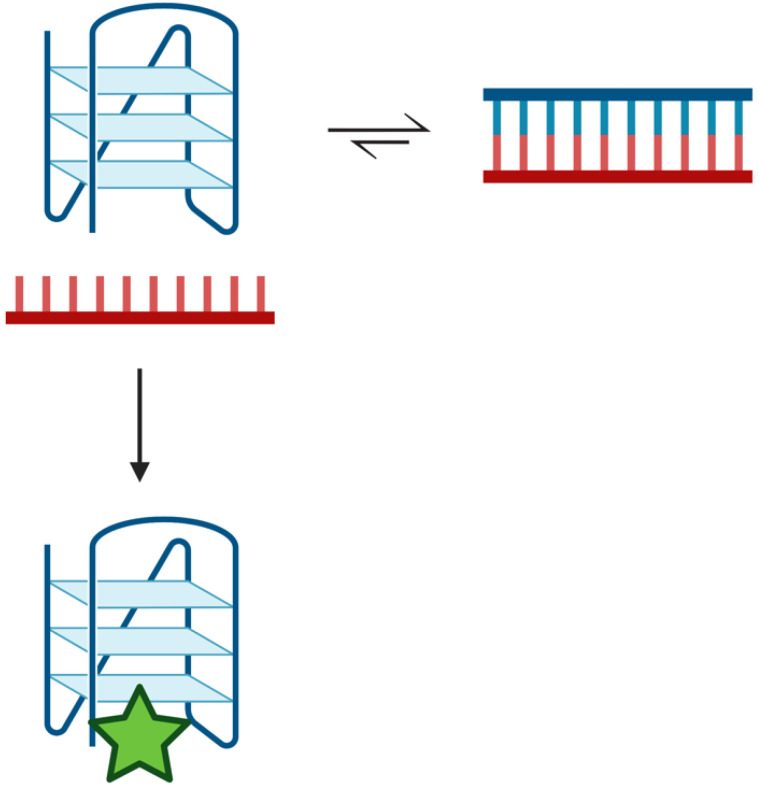
Duplex–quadruplex equilibrium scheme. Potentially quadruplex-forming regions of the genome are in equilibrium with duplex. Duplex is the preferred configuration in the presence of the complementary strand [22,23,24]. Transiently, a quadruplex may form that can be ‘trapped’ by quadruplex-binding ligands, such as pyridostatin (green star). The ligand stabilizes the quadruplex structure such that it prevents its unfolding and reforming duplex. Created with Biorender.com.

Previous studies have suggested that the toxicity of quadruplex-stabilizing small molecules, such as pyridostatin, might be attributed to the stabilization of quadruplex structures, which could inhibit DNA replication [45]. We examined the capacity of pyridostatin to drive the duplex DNA towards quadruplex formation (Figure 5C). Unexpectedly with increasing concentrations of pyridostatin, a duplex formed from a quadruplex-forming 22-mer (Tel22) and its complementary strand (A:U) was shifted towards quadruplex formation. Despite introducing an abasic site into the C-rich complementary strand, a similar concentration of pyridostatin was required to generate predominantly quadruplex DNA. Upon introduction of a gap, a similar amount of pyridostatin was needed to form quadruplex, although this destabilized the duplex configuration. This indicates that the binding affinity of pyridostatin to the quadruplex was unchanged since the quadruplex sequence itself was otherwise unaltered. Therefore, we propose that despite duplex being favored under physiologic conditions, duplex and quadruplex can rapidly interconvert and pyridostatin serves to trap the quadruplex configuration—preventing its conversion back to duplex (Figure 7). This was demonstrated when we added a 50-fold excess of pyridostatin relative to DNA and trapped all available quadruplex-forming oligonucleotides (Figure 5C). Thus, the cytotoxicity of pyridostatin may be attributed to stabilizing existing quadruplexes but also trapping the quadruplex configuration in the apparently hundreds of thousands of quadruplex-forming regions that are in equilibrium with duplex [6]. Furthermore, the duplex–quadruplex equilibrium may similarly be influenced and regulated by the presence of quadruplex-binding enzymes [29,36,45,46,47].

### 3.4. Conclusions and Limitations of This Study

The biological functions of unusual, non-duplex structures in DNA are not well understood. However, the work presented here demonstrates that the introduction of some modified bases into DNA, and the subsequent conversion to intermediates of the BER pathway, can alter duplex–quadruplex equilibrium under physiological conditions. The intersection of BER repair intermediates generated by the repair of pyrimidine analogs and unusual DNA structures results in complexities that may contribute to the cytotoxicity of these analogs that has implications for the development of chemotherapies. Within the context of antimicrobial or antitumor chemotherapy, various pyrimidine analogs could potentially be used in combinations where specific glycosylases are known to be differentially expressed. It remains unclear if overexpression and high activity of DNA glycosylases, which would promote strand-breaks, would be more cytotoxic than a lack of repair activity, resulting in an accumulation of the nucleoside analog and potential disrupt ion of DNA-protein interaction. 

This study focused on the human telomeric repeat sequence, which is one of the most well-studied DNA quadruplex-forming sequences [12,16,21]. The similarity among quadruplex-forming oligonucleotides is the core of hydrogen-bonding guanine bases, and variability is created by the length and base composition of the loop regions. The results of our study might not be generally extendible to other sequences with varying loop lengths, such as *c-MYC* and *hTERT* promoters, although duplex to quadruplex transition with small molecules has been shown with the *c-MYC* promoter [36,48]. 

Structural restraints induced by the quadruplex might interfere with base extrusion and therefore glycosylase excision [28]. Monofunctional glycosylases recognize a damaged or modified base, generally in a duplex, and extrude that damaged base from the helix into a binding pocket. Short loops like those found in the human telomere appear to interfere with base extrusion and therefore glycosylase excision. However, if the enzyme is active on single-strand DNA and a loop is of sufficient length, target bases might be more readily excised by monofunctional glycosylase and the abasic site cleaved by an endonuclease or lyase [28].

The modified bases examined here, U, 5hmU, and 5FU are targets of the monofunctional glycosylases of the BER pathway, but not of the bifunctional glycosylases. Therefore, the implications of this study are limited to targets of the monofunctional glycosylases. Bifunctional glycosylases, including FPG and OGG1, are also components of BER. The bifunctional glycosylases excise target bases such as 8-oxoguanine and cleave the DNA backbone resulting in simultaneous strand cleavage. Further studies are in progress to examine the activity of the bifunctional glycosylases on DNA sequences that can form alternative structures. 

## 4. Materials and Methods

### 4.1. Oligonucleotide Synthesis 

Oligonucleotides were synthesized by solid-phase phosphoramidite methods and purified using HPLC. All phosphoramidites, including those for the modified bases U, 5hmU, 5FU, THF, 6FAM, and BHQ1, were obtained from Glen Research. Oligonucleotide composition was verified using MALDI-MS and enzymatic digestion, followed by HPLC analysis (Supplementary Section S1). Oligonucleotide purity was verified using gel electrophoresis. 

### 4.2. Enzymes

*E. coli* uracil DNA glycosylase (UDG, ca# M0280S), human AP endonuclease 1 (APE1, ca# M0282S), and human single-strand selective monofunctional uracil DNA glycosylase (hSMUG1, ca# M0336S) were purchased from New England Biolabs (NEB). 

### 4.3. Buffers and Reagents

UDG reactions were conducted in a buffer containing 15 mM NaCl, 150 mM KCl, 20 mM Tris, and pH 7.4. APE1 reactions were conducted in buffer containing 15 mM NaCl, 150 mM KCl, 10 mM magnesium acetate (Mg-Ac), 20 mM Tris, pH 7.4. hSMUG1 reactions were conducted in a buffer containing 15 mM NaCl, 50 mM KCl, 20 mM Tris, and pH 7.4. Unless otherwise stated, all chemicals were purchased from Sigma-Aldrich. Pyridostatin was purchased from APExBIO (ca# A3742) and dissolved in double-deionized water and stored at 4 °C or −20 °C for longer term storage. 

### 4.4. CD Spectroscopy Studies

CD spectra were obtained on a Jasco J-815 CD spectrometer. Spectra were obtained at 37 °C from 320 to 220 nm. Oligonucleotides (4 μM) were typically prepared in a buffer containing 20 mM Tris, pH 7.4, 15 mM NaCl, and 150 mM KCl. For the K^+^ titration experiments, NaCl was excluded, and the KCl concentration was varied from 0 to 150 mM KCl. Oligonucleotides were equilibrated at room temperature (RT) for at least 30 min before spectra were acquired. Spectra were acquired in a 100 μL cuvette with a 1 cm path length (Starna, ca#26.100LHS-Q-10/Z15). Pyridostatin was added to some oligonucleotides as indicated in the figure legends to measure its effect.

### 4.5. Fluorescence Studies

Solutions containing oligonucleotides with a 5′-6FAM fluorophore and a 3′-BHQ1 quencher were prepared as a 1 μM solution (100 pmol in 100 μL) in 20 mM Tris buffer, pH 7.4, 15 mM NaCl, and 150 mM KCl unless otherwise indicated using the Jasco J-815. For duplex formation, 1.2 equivalents (120 pmol) of the C-rich complementary strand were annealed at 90 °C for 5 min and cooled to RT. All samples were equilibrated for 30 min at RT prior to the acquisition of fluorescence spectra.

When comparing fluorescence spectra from different oligonucleotides, absolute fluorescence varied among the samples. Fluorescence intensity for each oligonucleotide was therefore normalized to the fluorescence intensity measured in the absence of added K^+^ or Na^+^ cations, in 20 mM Tris buffer at pH 7.4.

Fluorescence emission spectra were acquired at 37 °C from 480 to 640 nm in 1 nm intervals with an excitation of 495 nm using a Jasco J-815 CD spectrometer equipped with a fluorimeter. Sensitivity was set to a default 600 V, D.I.T 0.125 s, and bandwidth set to 10 nm. A 100 μL fluorometer cuvette was used (Starna, ca# 16.100F-Q-10/Z15). 

To examine the effect of BER intermediates on the duplex–quadruplex equilibrium, the labeled quadruplex strand was annealed with 1.2 equivalents (120 pmol) of each complementary strand containing C, U, a THF abasic site or a one-base gap in 20 mM Tris buffer pH 7.4 with 150 mM KCl and 15 mM NaCl, heated to 90 °C for 5 min and cooled to RT. Spectra were then acquired as described above.

To simulate BER intermediates prepared enzymatically, we used UDG (abasic site), or UDG and APE1 (one-base gap). The fluorescent emission spectrum was taken for the quadruplex-only strand and a duplex oligonucleotide containing a U in the C-rich strand (A:U). In addition, spectra were also acquired for the duplex incubated with 10 U of UDG (3.39 pmol, 34 nM), and duplex incubated with 10 U of UDG (3.39 pmol, 34 nM) and 20 U of APE1 (0.71 pmol, 7.1 nM) for 2 h at 37 °C. For all samples, 10 mM Mg-Ac was supplemented to each reaction.

### 4.6. Quadruplex Fluorescence-Based Kinetic Studies and Pyridostatin Titration

Kinetic measurements were performed in 96-well plates on a Roche 480 Lightcycler II qPCR instrument using the default excitation and emission filters for fluorescein (FAM). Pyridostatin was added to some oligonucleotides, as indicated in the figure legends, and fluorescence was measured after oligonucleotides were incubated with increasing amounts of pyridostatin for 30 min at 37 °C.

The quadruplex unfolding and duplex formation time-course experiments were conducted on a Roche 480 qPCR instrument at 37 °C. Quadruplex-forming oligonucleotides (25 pmol, 1 μM) in 20 mM Tris buffer, pH 7.4, 15 mM NaCl, and 150 mM KCl were first equilibrated for 30 min at RT, at a final volume of 25 µL. Each reaction was then placed on ice for ~20 min and an equimolar amount (25 pmol) of unlabeled complementary strand was added. Fluorescence emission intensity at ~520 nm was then measured every 20 s for 35 min and every 60 s for the remaining 285 min. The data were fit to a two-phase exponential and plotted with GraphPad PRISM 9.4.1. These experiments were performed in triplicate.

We monitored structural changes in a quadruplex-forming strand of a duplex as U in the complementary C-rich strand was removed. The U-containing duplex was incubated simultaneously with 1.25 U of UDG (0.42 pmol, 16.8 nM) and 2.5 U of APE1 (0.09 pmol, 3.6 nM) in the buffer described above supplemented with 10 mM Mg-Ac. Fluorescence intensity was measured every 20 s at 37 °C. A single exponential decay was used to fit the data, and all studies were performed in triplicate.

### 4.7. Native Gel Electrophoresis of DNA Repair Intermediates

Oligonucleotides used in the above studies were examined using 20% polyacrylamide native gel electrophoresis (PAGE) in 1× TBE buffer (Fisher Scientific, Hampton, NH, USA). Oligonucleotide mixtures (2.5 pmol) were mixed with an equal volume of 20% glycerol and loaded onto the gels and electrophoresed for 70 min at 180 V, 4 °C.

### 4.8. DNA Glycosylase and AP Endonuclease Studies by Denaturing Gel Electrophoresis

Time points were taken from 2.5 pmol aliquots (12.5 μL, 0.2 μM) of a single reaction, per analog, that consisted of 28.75 pmol (0.2 μM) of oligonucleotide in 144 μL. Reactions were prepared in the appropriate buffer for each enzyme and equilibrated at RT for 30 min. After 30 min, the negative control aliquot containing no enzyme was taken. Enzyme was then added, and the reaction incubated at 37 °C. Aliquots were then taken at each indicated time point up to 60 min. Reactions were quenched by mixing an aliquot (12.5 μL) with 3 μL of 1 M NaOH and placing on ice. All reactions were performed with 3–4 replicates. 

UDG reactions with uracil-containing DNA had 0.26 U of UDG (0.0882 pmol, 0.613 nM), which is a 300:1 DNA:enzyme ratio. UDG reactions with 5FU-containing DNA had 2.6 U of UDG (0.882 pmol, 6.13 nM), which is a 30:1 ratio. hSMUG1 reactions with U and 5hmU-containing DNA used 0.8925 pmol (6.2 nM) of hSMUG1 (13.1 U), which is a 30:1 ratio. APE1 reactions used 0.189 pmol (1.31 nM) of APE1 (5.3 U), which is a 140:1 ratio. 

Reactions containing UDG were in a buffer containing 15 mM NaCl, 150 mM KCl, 20 mM Tris, pH 7.4. APE1 reactions were prepared in an identical buffer but supplemented with 10 mM Mg-Ac. Reactions with hSMUG1 contained 15 mM NaCl, 50 mM KCl, 20 mM Tris, pH 7.4. The KCl concentration was reduced to 50 mM as hSMUG1 activity is salt-dependent [27]. 

Following glycosylase removal of a target pyrimidine, the DNA backbone was cleaved by addition of 3 μL 1 M NaOH and heating at 95 °C for 10 min. Each reaction was then neutralized with 3 μL of 1 M acetic acid and an equal volume of formamide was added. Before loading gels, all reactions were heated to 95 °C for 1 min. Samples were then loaded onto a 6 M urea, 20% PAGE gel and run at 180 V for 70 min. A single exponential was fit to the data and rate constants were obtained using PRISM 9.4.1. The rate constants are reported in Table S1. The raw unnormalized gels corresponding to these experiments are shown in the supplementary information (Figures S1–S15).

### 4.9. Spontaneous β-Elimination Time Course

To measure spontaneous β-elimination of an abasic site at 37 °C, a quadruplex-forming oligonucleotide containing a U (100 pmol in 100 μL, 1 μM) was first incubated in 15 mM NaCl, 150 mM KCl, 20 mM Tris, pH 7.4 at 37 °C with 3.36 pmol (33.6 nM) UDG (10 U). Aliquots were taken at selected times and stored at −20 °C. Samples were then loaded and run on a denaturing gel as described above. The amount of cleaved product was quantified, and the rate of β-elimination was determined by linear regression of data points obtained from 1 h to 28.5 h. Reactions were performed in triplicate (Figure S16). The percentage of β-elimination at each time point was normalized to the total amount of cleavage observed in a sample incubated for 3 h with UDG at 37 °C followed by NaOH-induced cleavage. 

### 4.10. Gel Quantification and Statistical Analysis

Gels images were analyzed in ImageJ as previously described [26]. Error bars represent the standard deviation (S.D.) of three independent experiments. Error bars not seen indicate a S.D. smaller than the data point. 

## Data Availability

All data used to prepare this manuscript are available in the supporting information and Supplementary Section S1.

## Supplementary Materials

The following supporting information can be downloaded at: https://www.mdpi.com/article/10.3390/molecules28030970/s1, Table S1: DNA glycosylase and APE1 rate constants for Figure 3; Figure S1: Unedited gel scans of UDG time course with U:A substrate in Figure 3A; Figure S2: Unedited gel scans of UDG time course with Tel22-U substrate in Figure 3A; Figure S3: Unedited gel scans of UDG time course with NQ-U substrate in Figure 3A; Figure S4: Unedited gel scans of UDG time course with 5FU:A substrate in Figure 3B: Figure S5: Unedited gel scans of UDG time course with Tel22-5FU substrate in Figure 3B: Figure S6: Unedited gel scans of UDG time course with NQ-5FU substrate in Figure 3B; Figure S7: Unedited gel scans of hSMUG1 time course with substrate U:A in Figure 3C; Figure S8: Unedited gel scans of hSMUG1 time course with substrate Tel22-U in Figure 3C; Figure S9: Unedited gel scans of hSMUG1 time course with substrate NQ-U in Figure 3C; Figure S10: Unedited gel scans of hSMUG1 time course with substrate 5hmU:A in Figure 3D; Figure S11: Unedited gel scans of hSMUG1 time course with substrate Tel22-5hmU in Figure 3D; Figure S12: Unedited gel scans of hSMUG1 time course with substrate NQ-5hmU in Figure 3D; Figure S13: Unedited gel scans of APE1 time course with substrate THF:A in Figure 3E; Figure S14: Unedited gel scans of APE1 time course with substrate Tel22-THF in Figure 3E; Figure S15: Unedited gel scans of APE1 time course with substrate NQ-THF in Figure 3E; Figure S16: Unedited gel scans of the β-elimination time course with UDG treated substrate Tel22-U in Figure 3F; Figure S17: Quadruplexes quench fluorescence compared to duplex. In addition, when the quadruplex oligonucleotide is separated in half (quadruplex fragment), simulating a DNA repair gap, fluorescence is also no longer quenched and similar to the values seen for quadruplex strands annealed to their complement; Figure S18: Rate of gap formation in Figure 6 time course; Supplementary Section S1: This document contains the detailed synthesis and characterization for the oligonucleotides prepared and used in this study.
