# Supplementary material for: DNA Base Excision Repair Intermediates Influence Duplex–Quadruplex Equilibrium
Source: Molecules. 2023 Jan 18;28(3):970. doi: 10.3390/molecules28030970 (PMC9920732; doi:10.3390/molecules28030970)

Supplementary Appendix

Table of Contents

A.	Oligonucleotides synthesized and methods	2-3
B.	MALDI-MS+ results for oligonucleotides and methods	4-5
C.	MALDI-MS+ spectra of oligonucleotides	6-18
D.	HPLC results for nucleoside standards and methods	19
E.	UV spectra from HPLC PDA for nucleoside standards	20-21
F.	Enzyme digestion results for oligonucleotides and methods	22
G.	HPLC and UV spectra for enzyme digestion of oligonucleotides	23-35
H.	GC/MS results for oligonucleotides and methods	36
I.	GC/MS spectra of hydrolyzed and derivatized oligonucleotides	37-49

A. Oligonucleotides synthesized and methods

Table 1: Synthesis of Oligonucleotides

	Oligonucleotide	Sequence	Lot	Synthesis Conditions	Purification & Detritylation
1	Quad-22mer-T	5'-AGGGTTAGGGT**T**AGGGTTAGGG-3'	L-981	Standard A	Standard A
2	Quad-22mer-U	5'-AGGGTTAGGGT**U**AGGGTTAGGG-3'	L-9123	Standard C	Standard A
3	Quad-22mer-5FU	5'-AGGGTTAGGGT**5FU**AGGGTTAGGG-3'	L-9143	Standard C	Standard A
4	Quad-22mer-5hmU	5'-AGGGTTAGGGT**5hmU**AGGGTTAGGG-3'	L-1051	Standard D	Standard A
5	Quad-22mer-THF	5'-AGGGTTAGGGT**THF**AGGGTTAGGG-3'	L-1026	Standard A	Standard A
6	Quad-22mer-T-FAM	5'-6FAM-AGGGTTAGGGT**T**AGGGTTAGGG-3'	L-999	Standard A	Standard A
7	Quad-22mer-U-FAM	5'-6FAM-AGGGTTAGGGT**U**AGGGTTAGGG-3'	L-9113	Standard C	Standard A
8	Quad-22mer-5FU-FAM	5'-6FAM-AGGGTTAGGGT**5FU**AGGGTTAGGG-3'	L-1011	Standard C	Standard A
9	Quad-22mer-5hmU-FAM	5'-6FAM-AGGGTTAGGGT**5hmU**AGGGTTAGGG-3'	L-1041	Standard D	Standard A
10	Quad-22mer-THF-FAM	5'-6FAM-AGGGTTAGGGT**THF**AGGGTTAGGG-3'	L-1006	Standard C	Standard A
11	Quad-22mer-T-FAM-BHQ1	5'-6FAM-AGGGTTAGGGT**T**AGGGTTAGGG-BHQ1-3'	L-9102	Standard A	Standard A
12	Quad-22mer-U-FAM-BHQ1	5'-6FAM-AGGGTTAGGGT**U**AGGGTTAGGG-BHQ1-3'	L-9118	Standard C	Standard A
13	Quad-22mer-5FU-FAM-BHQ1	5'-6FAM-AGGGTTAGGGT**5FU**AGGGTTAGGG-BHQ1-3'	L-1016	Standard C	Standard A
14	Quad-22mer-5hmU-FAM-BHQ1	5'-6FAM-AGGGTTAGGGT**5hmU**AGGGTTAGGG-BHQ1-3'	L-1046	Standard D	Standard A
15	Quad-22mer-THF-FAM-BHQ1	5'-6FAM-AGGGTTAGGGT**THF**AGGGTTAGGG-BHQ1-3'	L-1021	Standard C	Standard A
16	Quad-11mer-FAM	5'-6FAM-AGGGTTAGGGT-3'	L-9105	Standard A	Standard A
17	Quad-10mer-BHQ1-phos	5'-pAGGGTTAGGG-BHQ1-3'	L-1079	Standard A	Standard B
18	Quad-22mer-T-Comp	3'-TCCCAATCCCAA**T**CCCAATCCC-5'	L-984	Standard A	Standard A
19	Quad-22mer-U-Comp	3'-TCCCAATCCCAA**U**CCCAATCCC-5'	L-9128	Standard A	Standard A
20	Quad-22mer-THF-Comp	3'-TCCCAATCCCAA**THF**CCCAATCCC-5'	L-1031	Standard A	Standard A
21	Quad-12mer-phos	3'-TCCCAATCCCAAp-5'	L-1082	Standard A	Standard B
22	Quad-9mer	3'-CCCAATCCC-5'	L-987	Standard A	Standard A
23	Quad-22mer-U-Comp-Cy5	3'-TCCCAATCCCAA**U**CCCAATCCC-Cy5-5'	L-1131	Standard C	Standard A
24	22mer-nonquad_5hmU	5'-6FAM-ACAGTTAGGGT**5hmU**AGGGTTACAC-3'	L-1154	Standard B	Standard A
25	22mer-nonquad_U	5'-6FAM-ACAGTTAGGGT**U**AGGGTTACAC-3'	L-1121	Standard A	Standard A
26	22mer-nonquad_compa	5'-GTGTAACCCT**A**ACCCTAACTGT-3'	L-1141	Standard A	Standard A

All oligonucleotides were synthesized on an Expedite 8909 DNA synthesizer.

Standard A: Oligonucleotides were synthesized using standard phosphoramidites (Bz-dA, Bz-dC, iBu-dG, dT) and appropriate modified phosphoramidite. The oligonucleotides were deprotected in ammonium hydroxide at 55°C for 15-17 hours.

Standard B: Oligonucleotides were synthesized using standard phosphoramidites (Bz-dA, iBu-dG, dT), Ac-dC and appropriate modified phosphoramidite. The oligonucleotides were deprotected in ammonium hydroxide at 55°C for 15-17 hours.

Standard C: Oligonucleotides were synthesized using standard phosphoramidites (Bz-dA, Bz-dC, iBu-dG, dT) and appropriate modified phosphoramidite. The oligonucleotides were deprotected in ammonium hydroxide at room temperature for 40-90 hours.

Standard D: Oligonucleotides were synthesized using standard phosphoramidites (Bz-dA, iBu-dG, dT), Ac-dC and appropriate modified phosphoramidite. The oligonucleotides were deprotected in ammonium hydroxide at 55°C for 39-64 hours.

Ultramild A: Oligonucleotides were synthesized using ultramild phosphoramidites (Pac-dA, Ac-dC, iPr-Pac-dG, dT) and appropriate modified phosphoramidite. The oligonucleotides were deprotected in ammonium hydroxide at room temperature for 15 hours.

Satndard A purification and detritylation by C18 Sep-pak: C18 Sep-pak cartridge (Waters WAT020515) was prepared by washing with acetonitrile (5 mL) and 1 M triethylammonium acetate (10 mL). The crude DMT-on oligonucleotide was loaded onto the cartridge in 1 M triethylammonium acetate (2 mL) then failure sequences were eluted with 10% ammonium hydroxide (5 mL) and water (5 mL). Detritylation was done with 2% trifluoroacetic acid (5 mL) and the cartridge washed with water (5 mL). The DMT-off oligonucleotide was then eluted with acetonitrile in water (2 mL of 20% ACN, 4 mL of 50% ACN).

Standard B purification and detritylation by C18 Sep-pak: C18 Sep-pak cartridge (Waters WAT020515) was prepared by washing with acetonitrile (5 mL) and 1 M triethylammonium acetate (10 mL). The crude DMT-on oligonucleotide was loaded onto the cartridge in 1 M triethylammonium acetate (2 mL) then failure sequences were eluted with 10% ammonium hydroxide (5 mL) and water (5 mL). Detritylation was done with 2% trifluoroacetic acid (5 mL) and the cartridge washed with water (5 mL). The DMT-off oligonucleotide was then eluted with acetonitrile in water (2 mL of 20% ACN, 4 mL of 50% ACN). The 5’-phosphate group was deprotected in ammonium hydroxide at room temperature for 15 hours.

B. MALDI-MS+ results for oligonucleotides and methods

Table 2: Maldi-MS of Oligonucleotides

		Maldi-MS+			
	Oligonucleotide	Expected (M+H)	Observed (M+H)	Δ	Data File (D:\Data\Linda)
1	Quad-22mer-T	6967.31	6968.6	+1.29	\03222022\L-981\0_A14\1\1SRef
2	Quad-22mer-U	6953.29	6950.78	-2.51	\03222022\L-9123\0_C1\1\1SRef
3	Quad-22mer-5FU	6971.28	6971.17	-0.11	\03252022\L-9143\0_H3\1\1SRef
4	Quad-22mer-5hmU	6983.31	6985.23	+1.92	\03252022\L-1051\0_G5\1\1SRef
5	Quad-22mer-THF	6843.22	6841.93	-1.29	\03222022\L-1026\0_C12\1\1SRef
6	Quad-22mer-T-FAM	7534.79	7535.22	+0.43	\03222022\L-999\0_B19\1\1SRef
7	Quad-22mer-U-FAM	7520.77	7521.43	+0.66	\03222022\L-9113\0_B23\1\1SRef
8	Quad-22mer-5FU-FAM	7538.76	7536.48	-2.28	\03222022\L-1011\0_D9\1\1SRef
9	Quad-22mer-5hmU-FAM	7550.79	7548.2	-2.59	\03222022\L-1041\0_D15\1\1SRef
10	Quad-22mer-THF-FAM	7410.7	7409.64	-1.06	\03222022\L-1006\0_D8\1\1SRef
11	Quad-22mer-T-FAM-BHQ1	8089.29	8087.99	-1.3	\03222022\L-9102\0_A20\1\1SRef
12	Quad-22mer-U-FAM-BHQ1	8075.26	8074.47	-0.79	\03252022\L-9118\0_G2\1\1SRef
13	Quad-22mer-5FU-FAM-BHQ1	8093.25	8094.45	+1.2	\03252022\L-1016\0_D10\1\1SRef
14	Quad-22mer-5hmU-FAM-BHQ1	8105.29	8103.98	-1.31	\03222022\L-1046\0_C16\1\1SRef
15	Quad-22mer-THF-FAM-BHQ1	7965.19	7965.8	+0.61	\03222022\L-1021\0_C11\1\1SRef
16	Quad-11mer-FAM	4020.66	4020.01	-0.65	\03222022\L-9105\0_B21\1\1SRef
17	Quad-10mer-BHQ1-phos	3783.48	3784.43	+0.95	\03252022\L-1079_15hr\0_G8\1\1SRef
18	Quad-22mer-T-Comp	6505.05	6504.11	-0.94	\03222022\L-984\0_A15\1\1SRef
19	Quad-22mer-U-Comp	6491.03	6470.6	-20.43	\03222022\L-9128\0_C2\1\1SRef
20	Quad-22mer-THF-Comp	6380.96	6378.75	-2.21	\03222022\L-1031\0_C13\1\1SRef
21	Quad-12mer-phos	3616.24	3614.58	-1.66	\03252022\L-1082_15hr\0_H10\1\1SRef
22	Quad-9mer	2604.66	2614.21	+9.55	\03222022\L-987\0_A16\1\1SRef
23	Quad-22mer-U-Comp-Cy5	7024.64	7023.71	-0.93	\04252022\L-1131\0_I6\1\1SRef
24	22mer-nonquad_5hmU	7398.72	7394.64	-4.08	\04252022\L-1154\0_J9\1\1SRef
25	22mer-nonquad_U	7368.7	7364.77	-3.93	\04252022\L-1121\0_I4\1\1SRef
26	22mer-nonquad_compa	6655.15	6652.14	-3.01	\04252022\L-1141\0_I3\1\1SRef

Desalting of C18 Sep-pak purified oligonucleotides: Micro BioSpin P6 column (BioRad 732-6221) was prepared by centrifugation at 1000G for 2 minutes. Water (500 µL) was added and the column washed by centrifugation at 1000G for 1 minute then a second wash was done. 1 OD of purified oligonucleotide in water (100 µL) was loaded and eluted into a new collection tube by centrifugation at 1000G for 4 minutes. The water was evaporated under reduced pressure and the oligo resuspended in water (25 µL).

Maldi Sample Preparation: 0.4 OD of HPLC purified or P6 BioSpin column desalted oligo in water (10 µL) was mixed with desalting ion exchange resin (2 µL) for 1 hour.

Desalting Ion Exchange Resin: Prepare slurry of cation exchange resin (6 g) in 50 / 50 acetonitrile / water (5 mL), pour into column and let settle (gravity). Wash resin with 50 / 50 acetonitrile / water (3-4 column volumes), 5% ammonium hydroxide (2 x 10 mL), 2 M ammonium acetate (3 x 10 mL) and water (3-4 column volumes). Aliquot into 1 mL fractions (50% suspension) and store -20°C.

Matrix: 3-hydroxypicolinic acid (70 mg) and ammonium citrate (10 mg) in 50/50 acetonitrile / water (1 mL) with 0.1% trifluoroacetic acid

The Maldi plate was spotted with HPA matrix (1 µL) and allowed to dry. The sample (1µL) was spotted on top of the matrix and allowed to dry before running on Bruker Autoflex Maldi-MS in positive mode.

Expected (M+H) values were calculated using average molecular weights.

C. MALDI-MS+ spectra of oligonucleotides

Quad-22mer-T

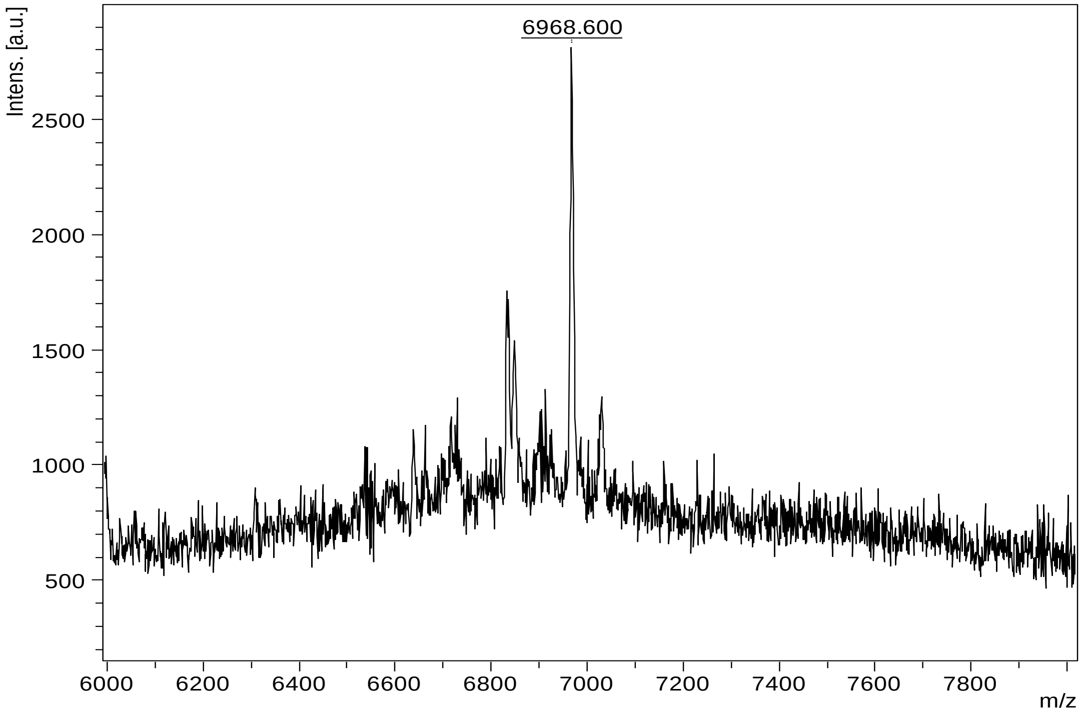

Quad-22mer-U

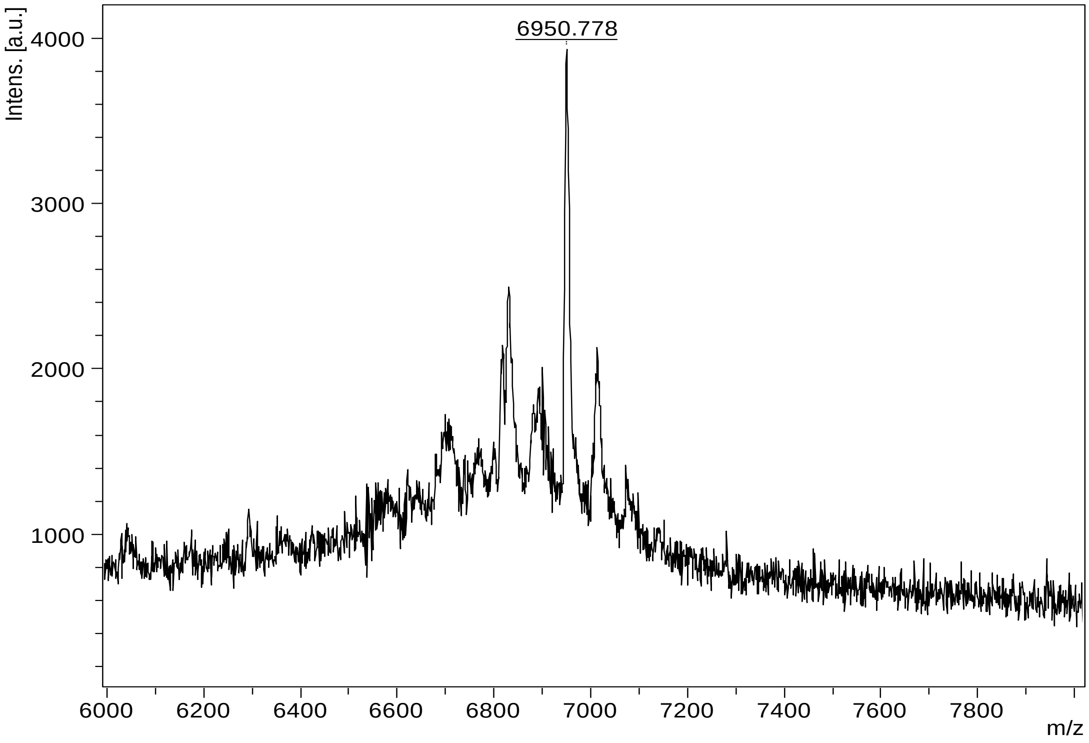

Quad-22mer-5FU

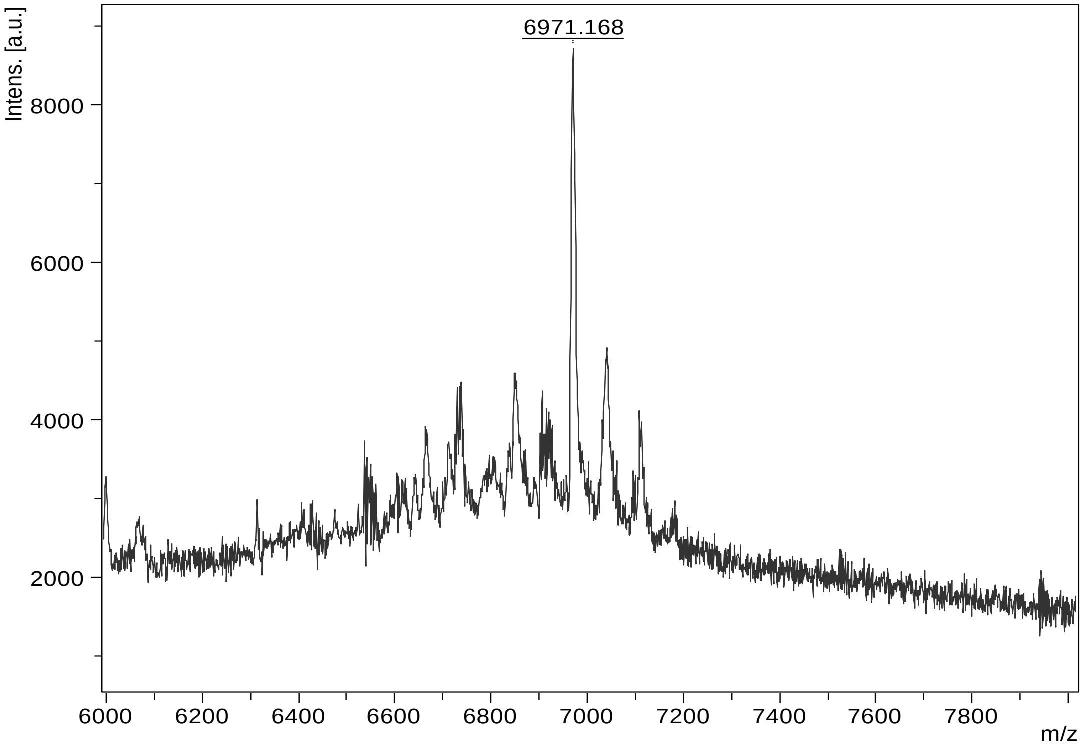

Quad-22mer-5hmU

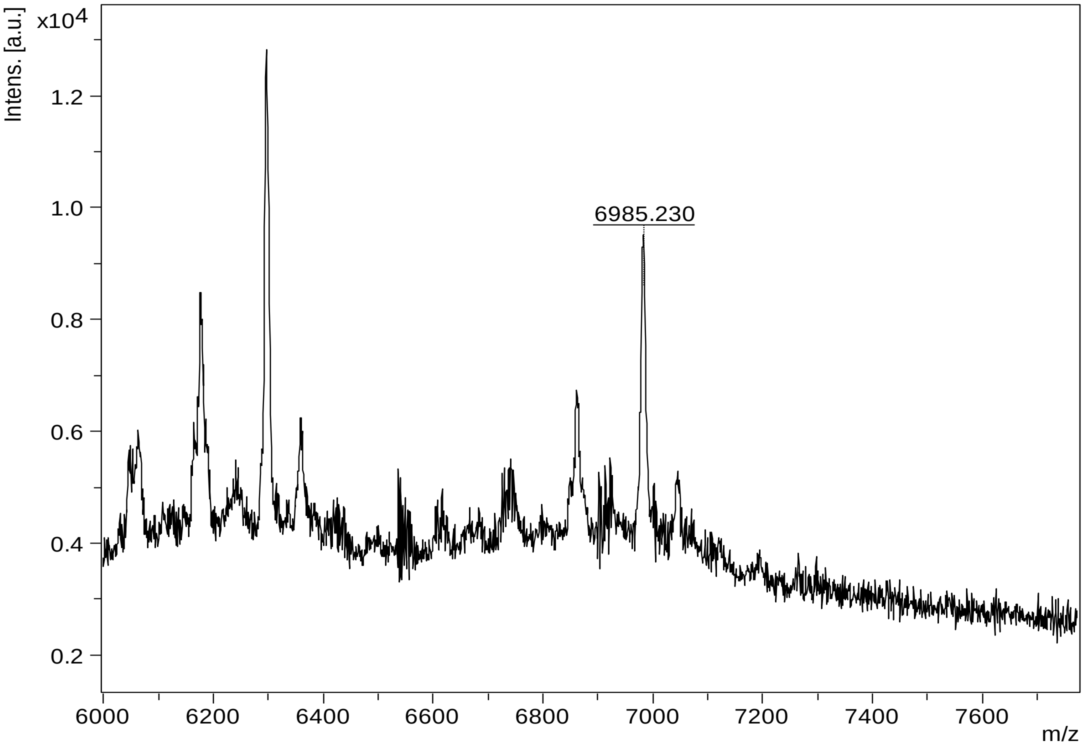

Quad-22mer-THF

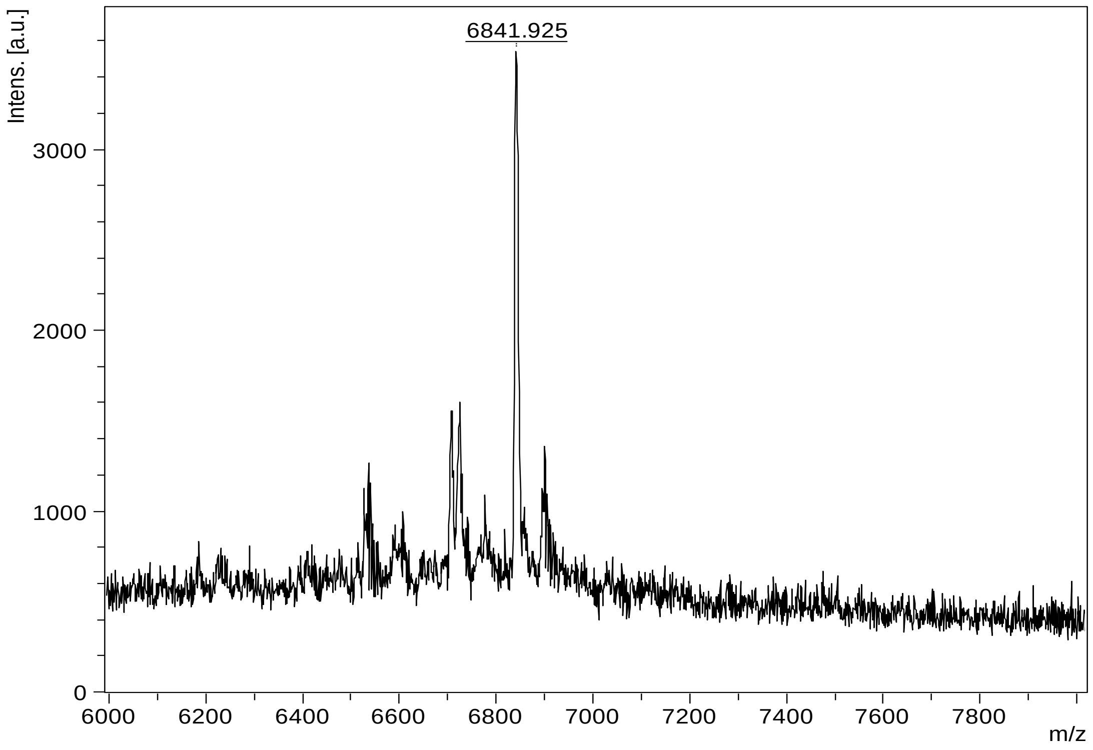

Quad-22mer-T-FAM

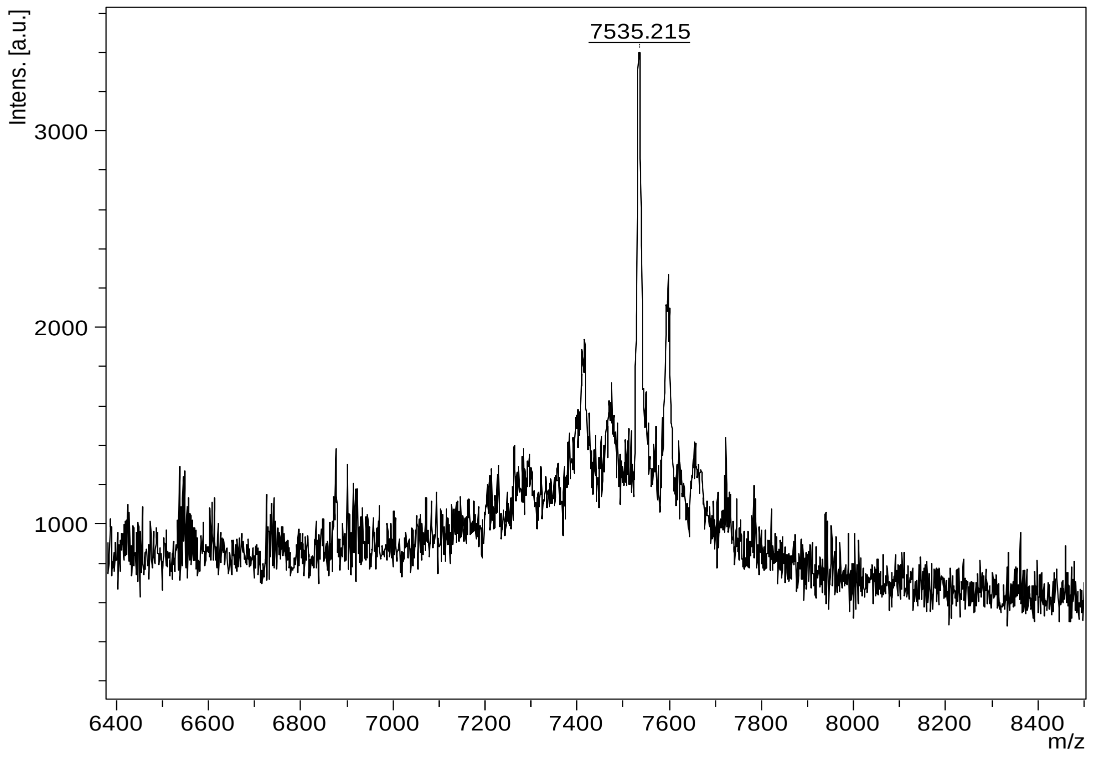

Quad-22mer-U-FAM

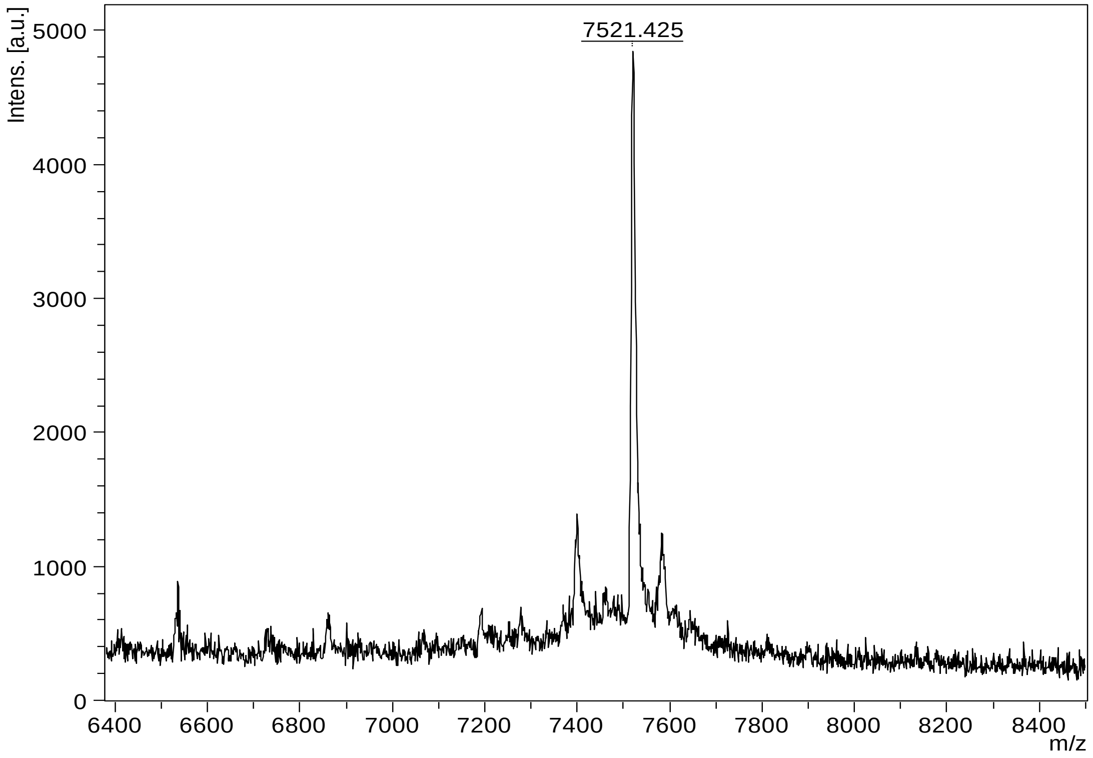

Quad-22mer-5FU-FAM

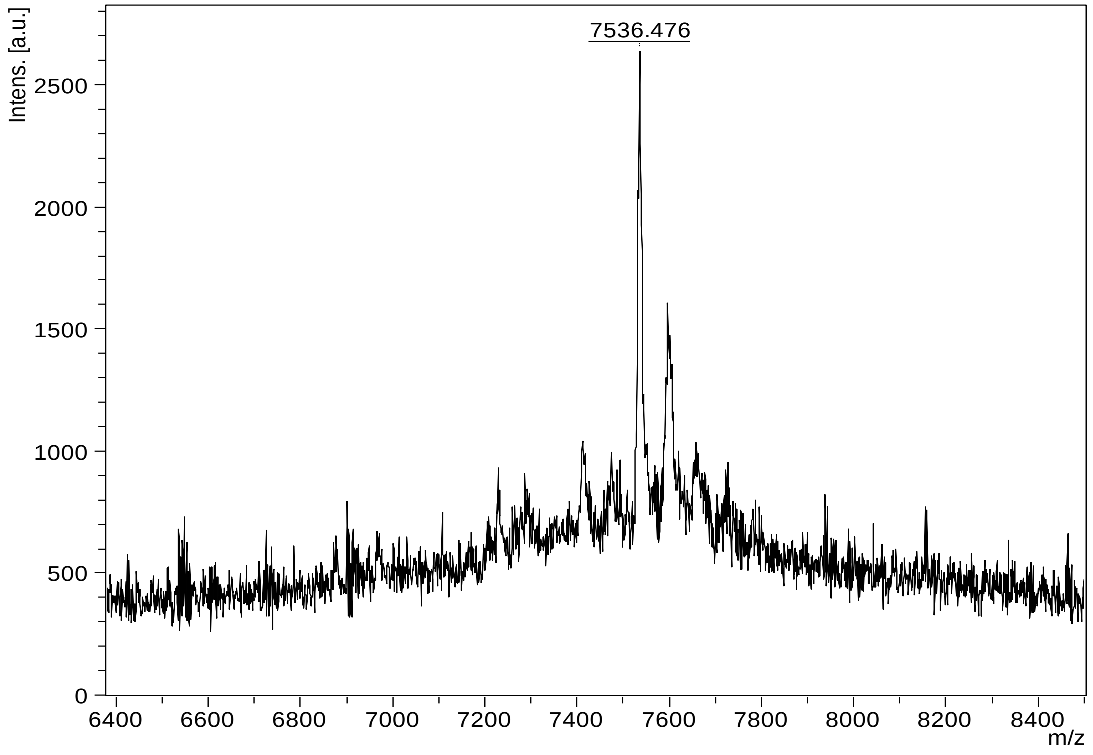

Quad-22mer-5hmU-FAM

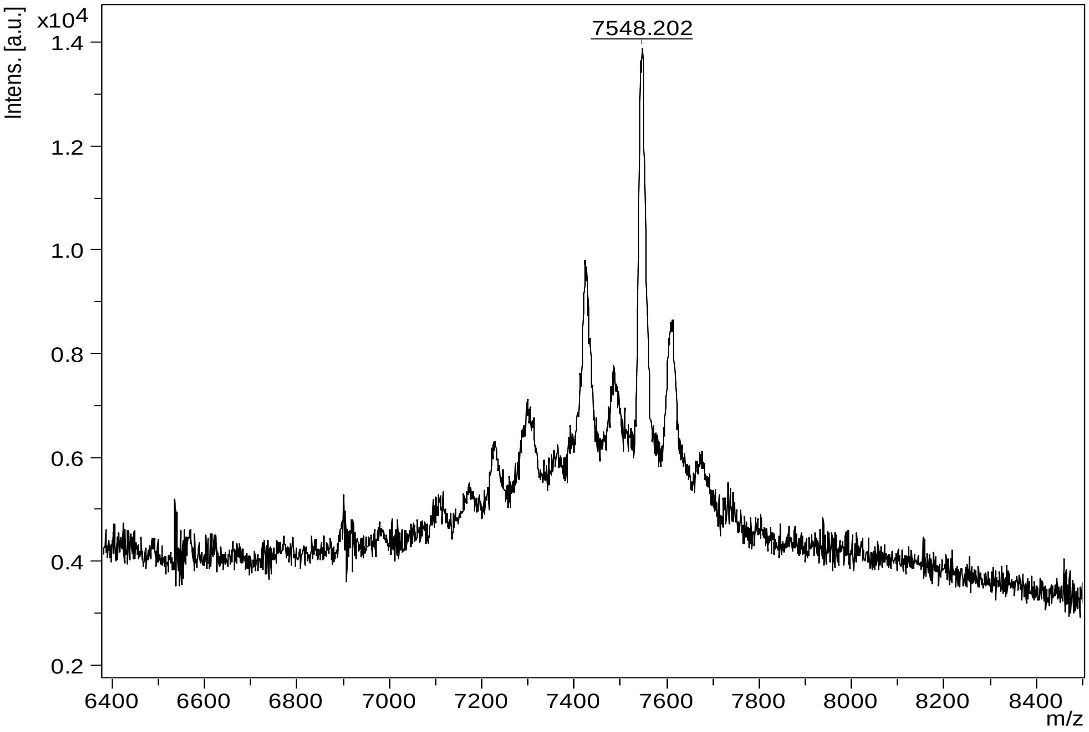

Quad-22mer-THF-FAM

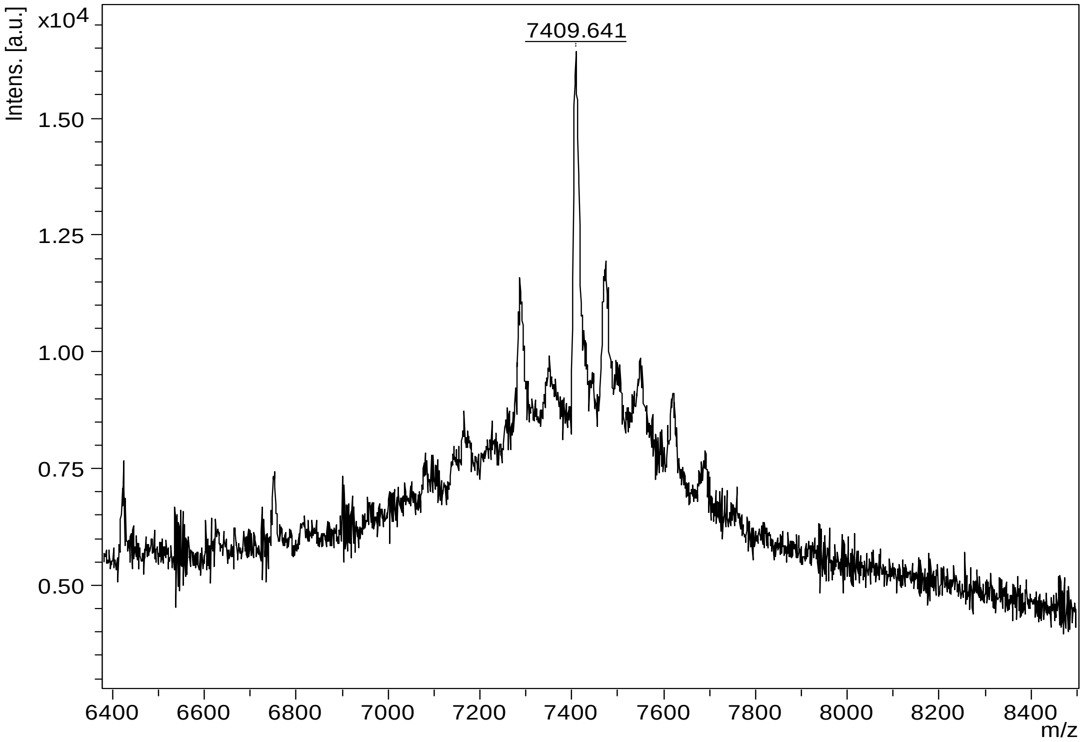

Quad-22mer-T-FAM-BHQ1

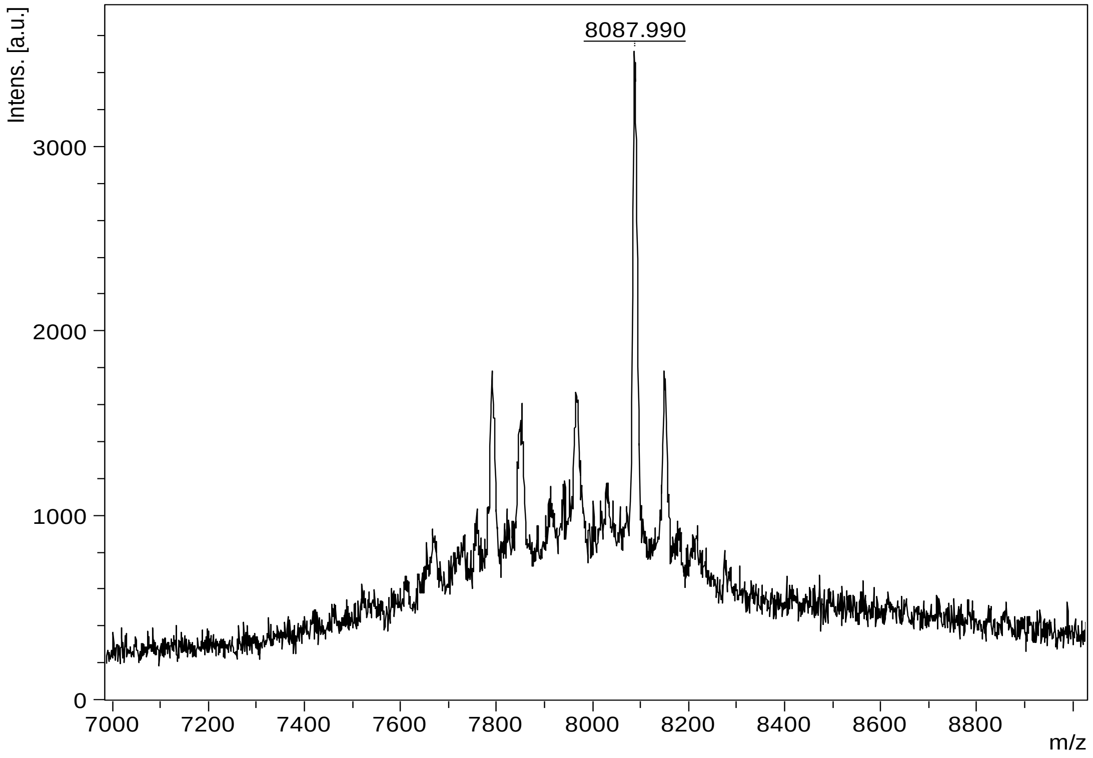

Quad-22mer-U-FAM-BHQ1

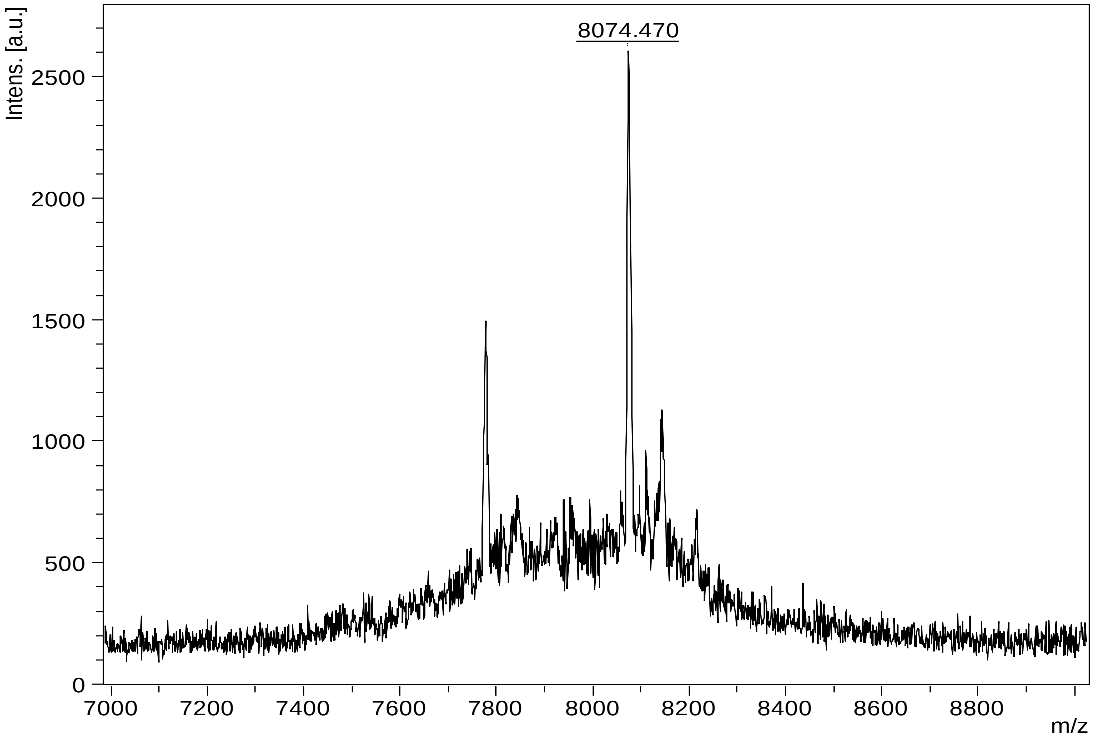

Quad-22mer-5FU-FAM-BHQ1

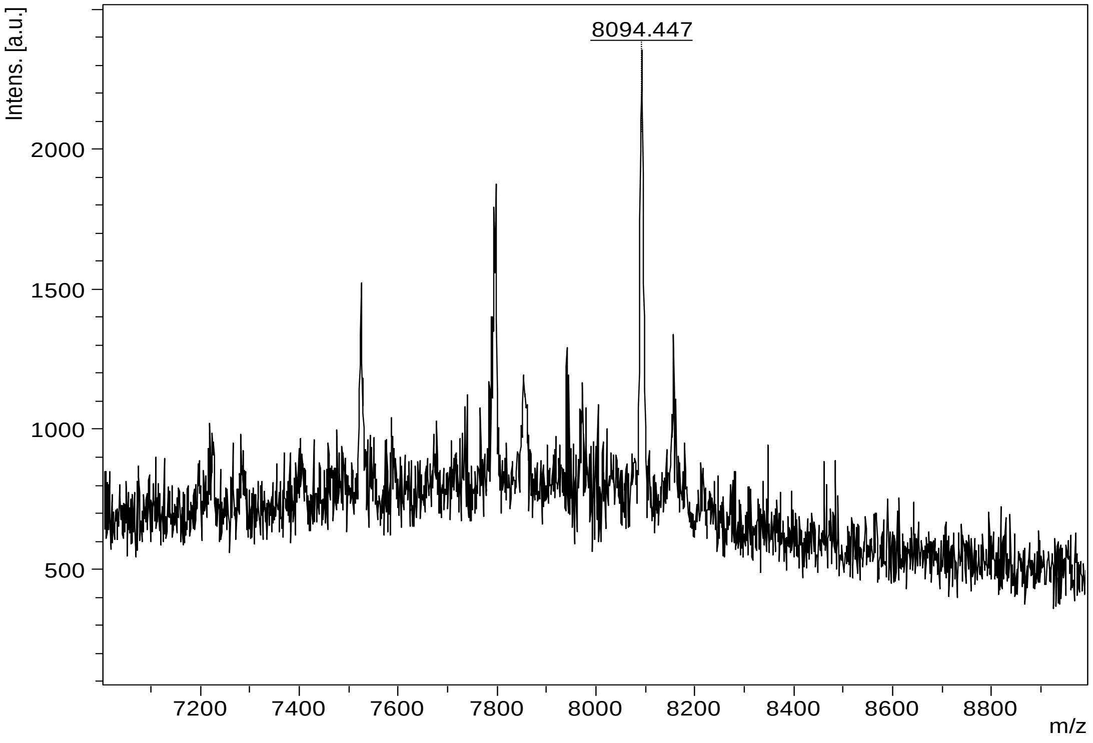

Quad-22mer-5hmU-FAM-BHQ1

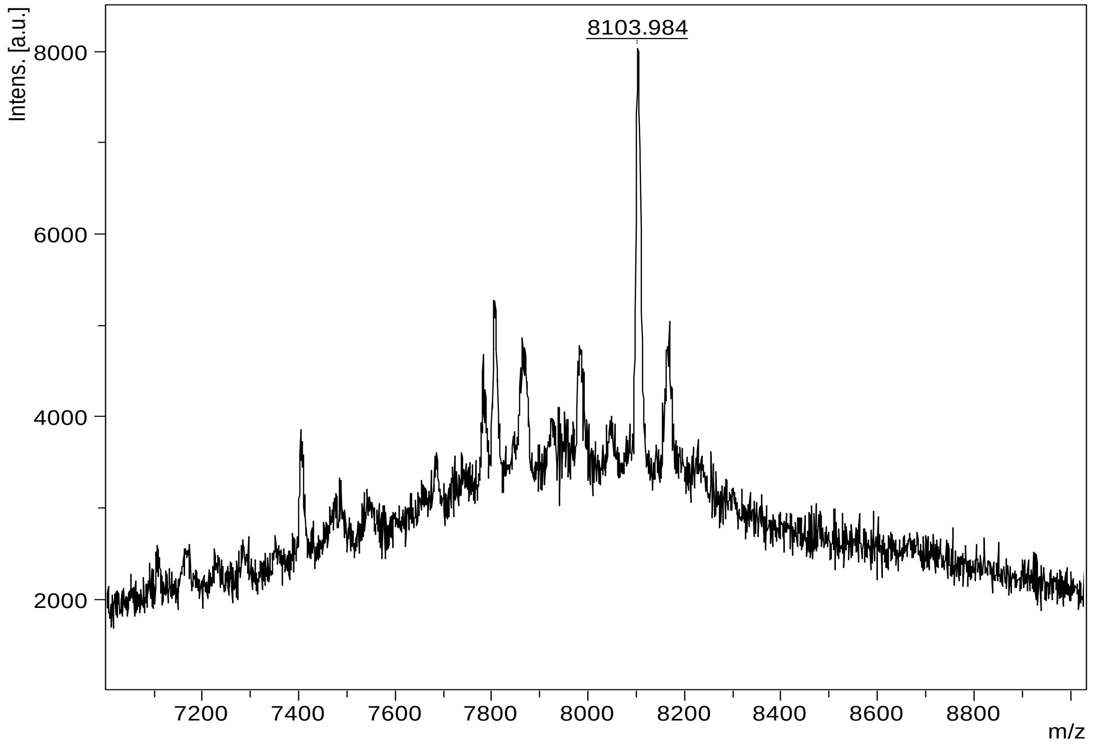

Quad-22mer-THF-FAM-BHQ1

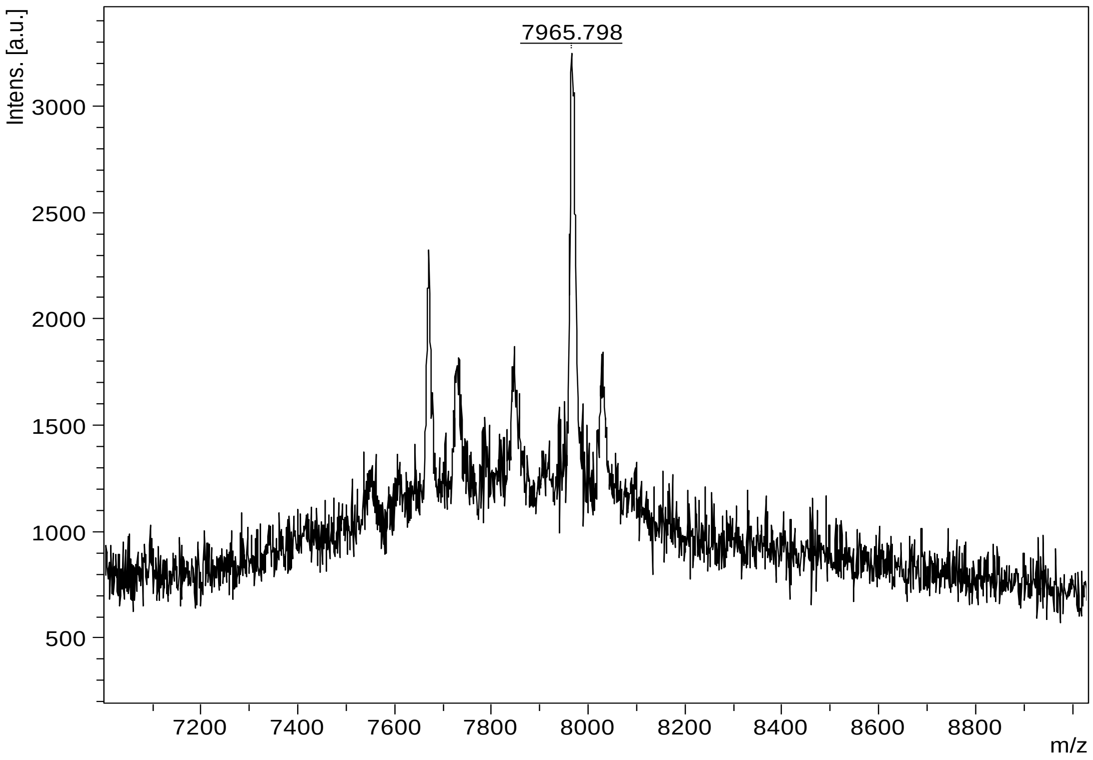

Quad-11mer-FAM

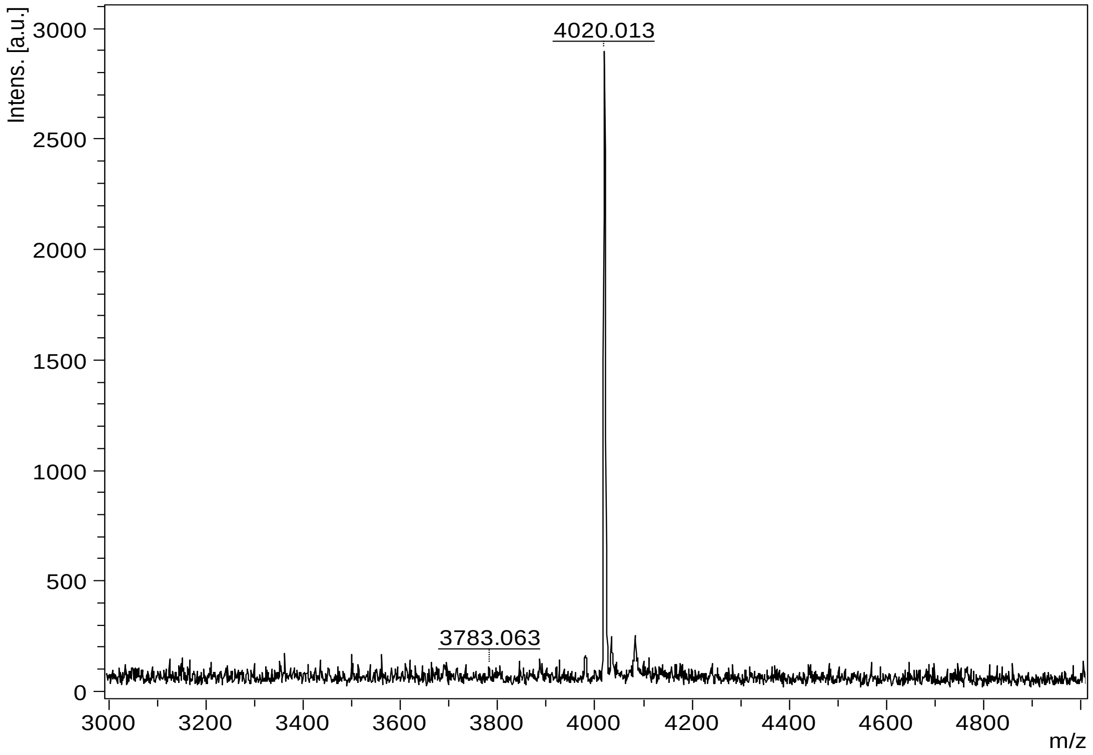

Quad-10mer-BHQ1-phos

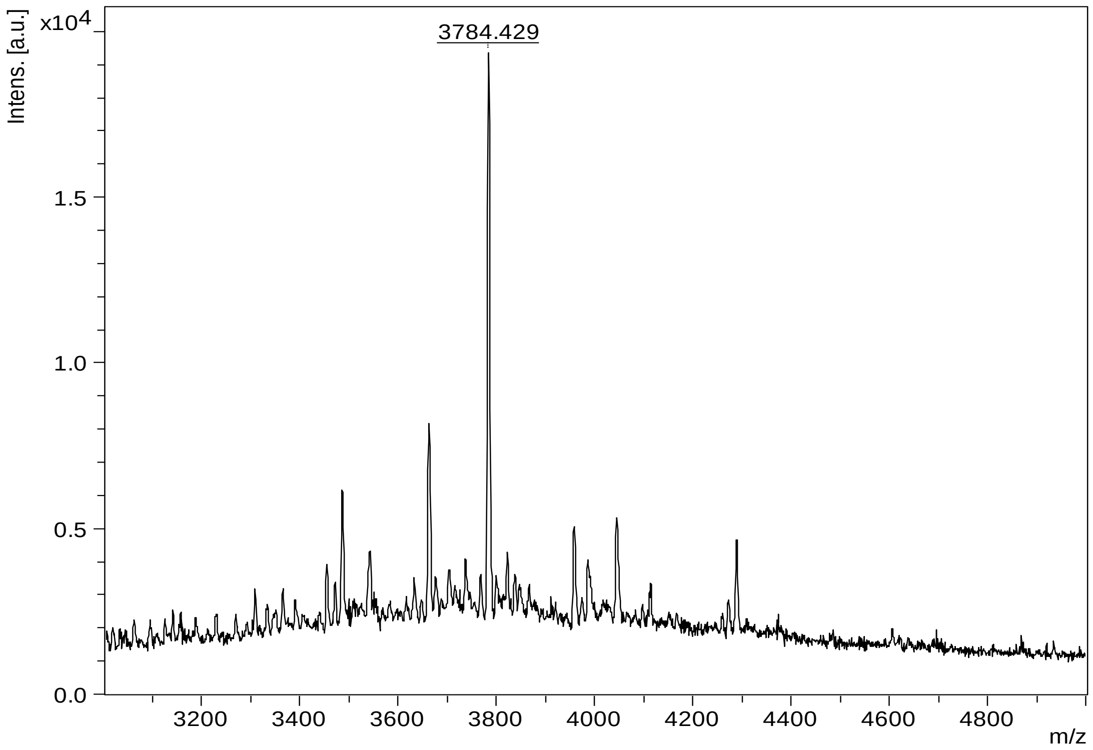

Quad-22mer-T-Comp

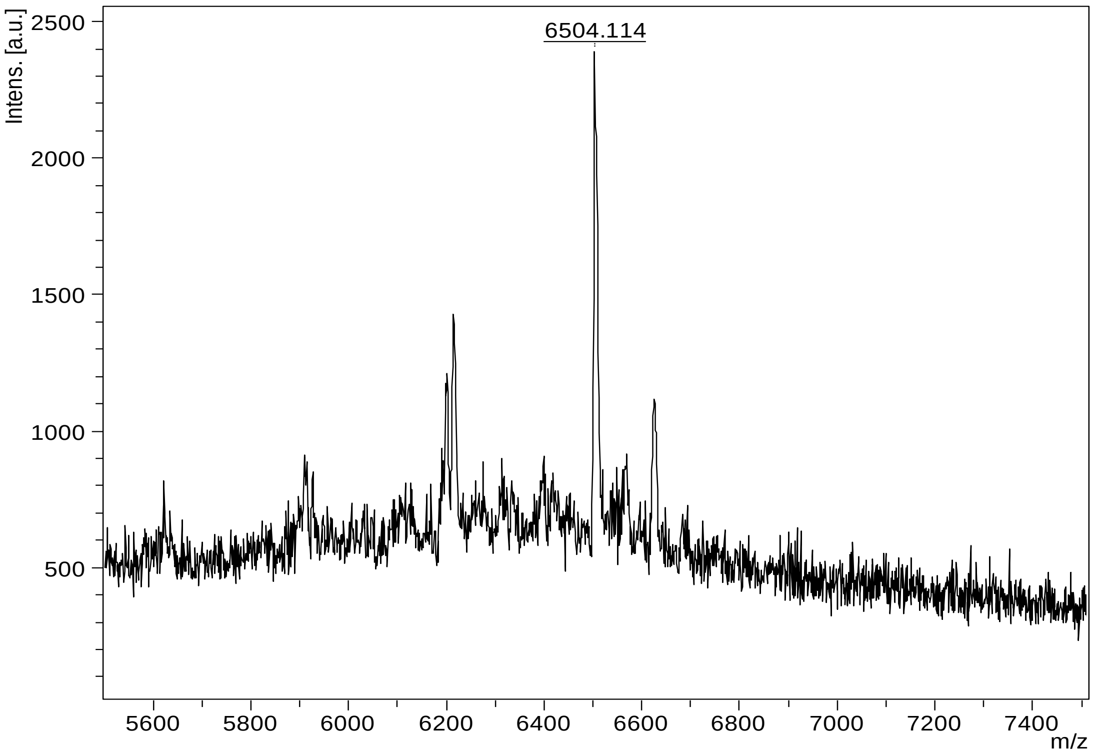

Quad-22mer-U-Comp

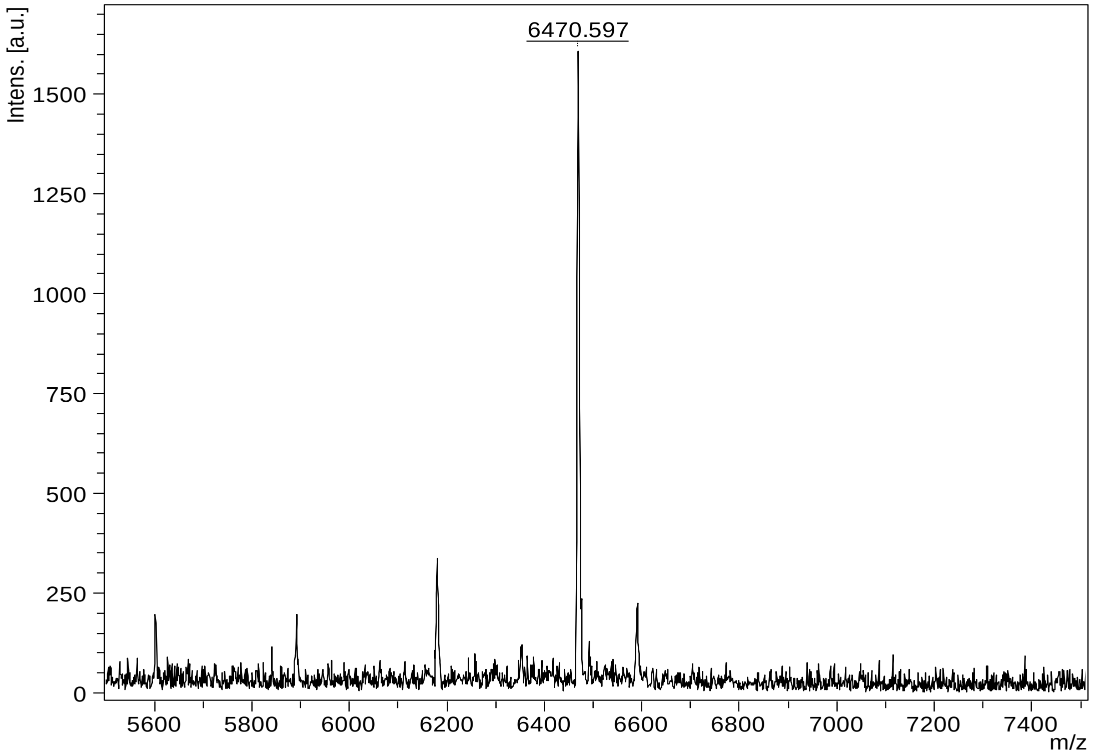

Quad-22mer-THF-Comp

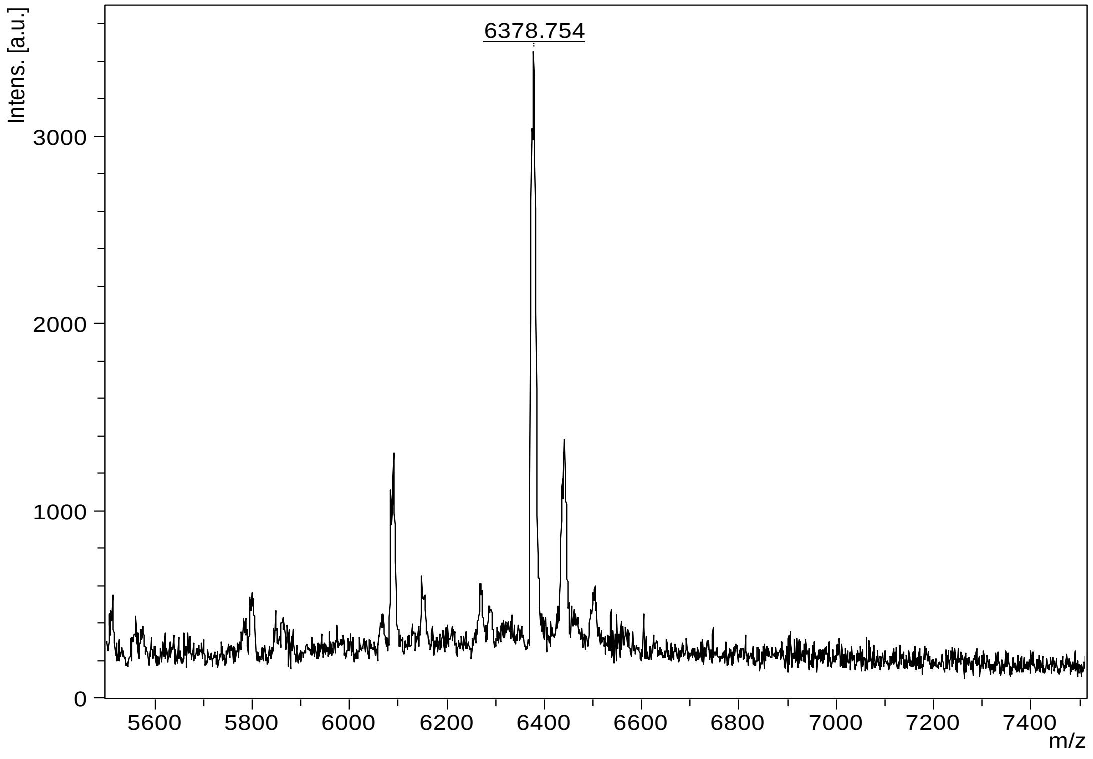

Quad-12mer-phos

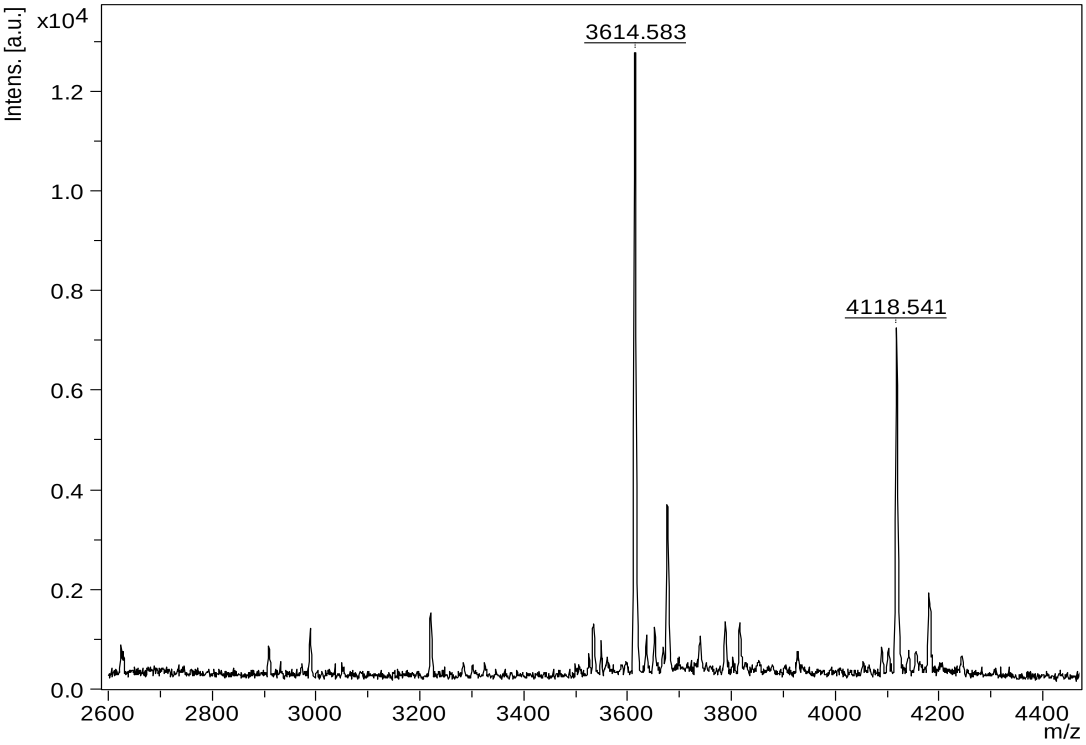

Quad-9mer

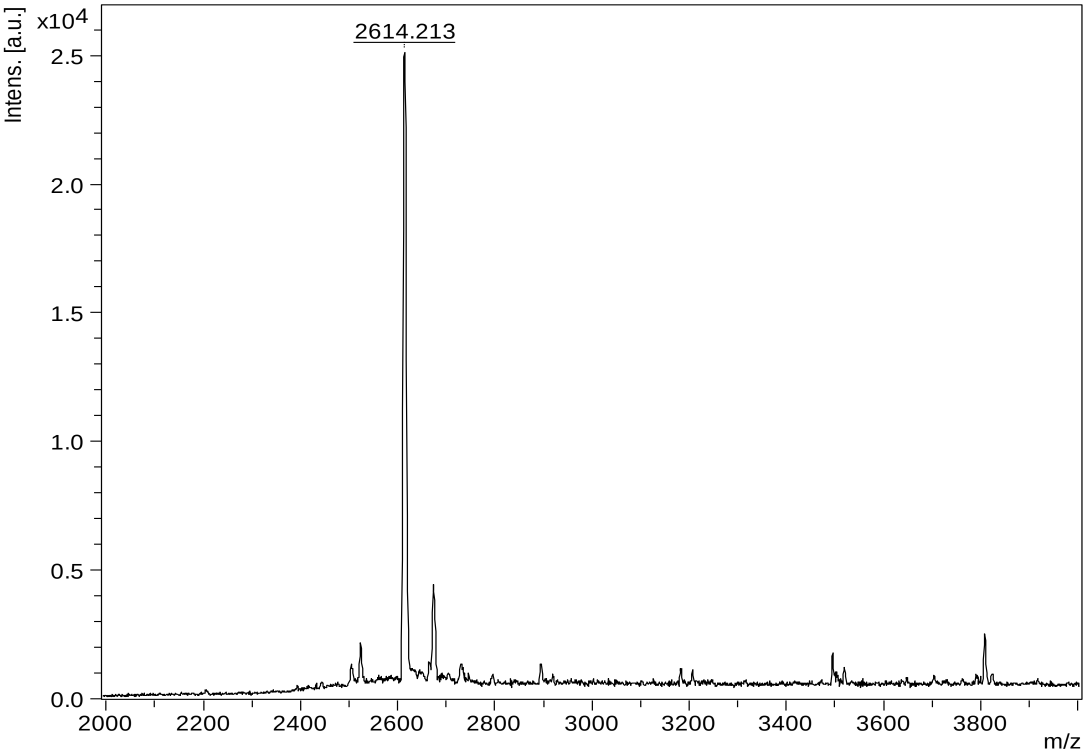

Quad-22mer-U-Comp-Cy5

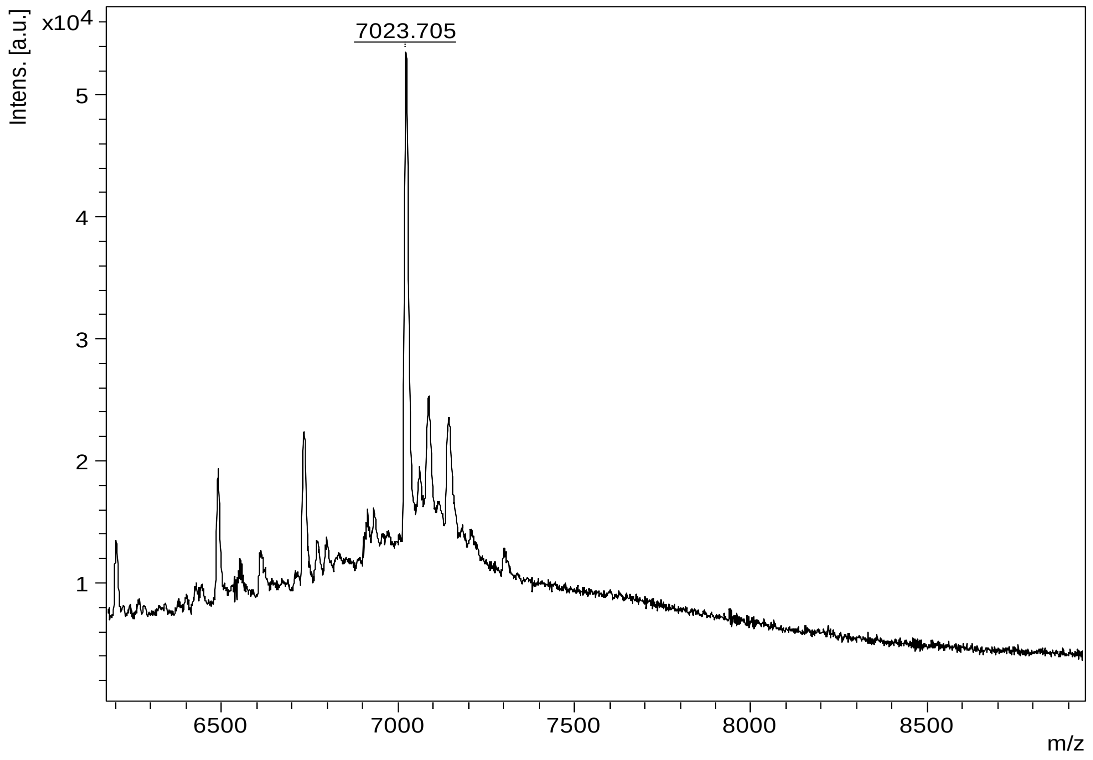

22mer-nonquad_5hmU

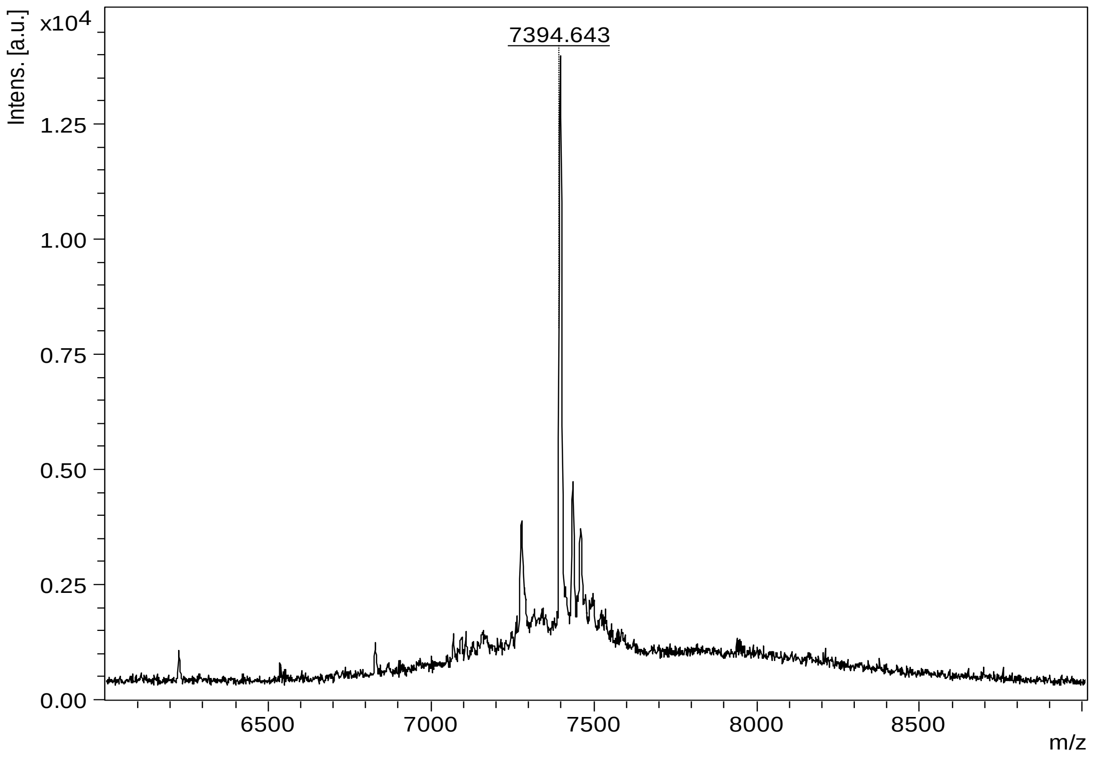

22mer-nonquad_U

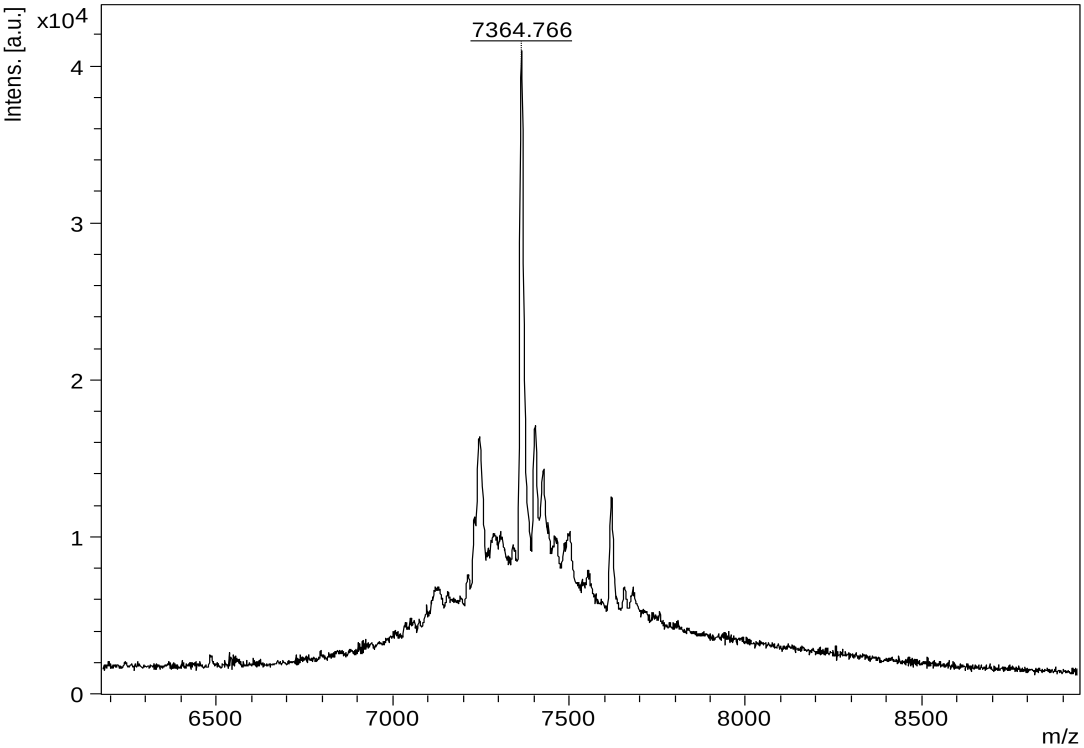

22mer-nonquad_compA

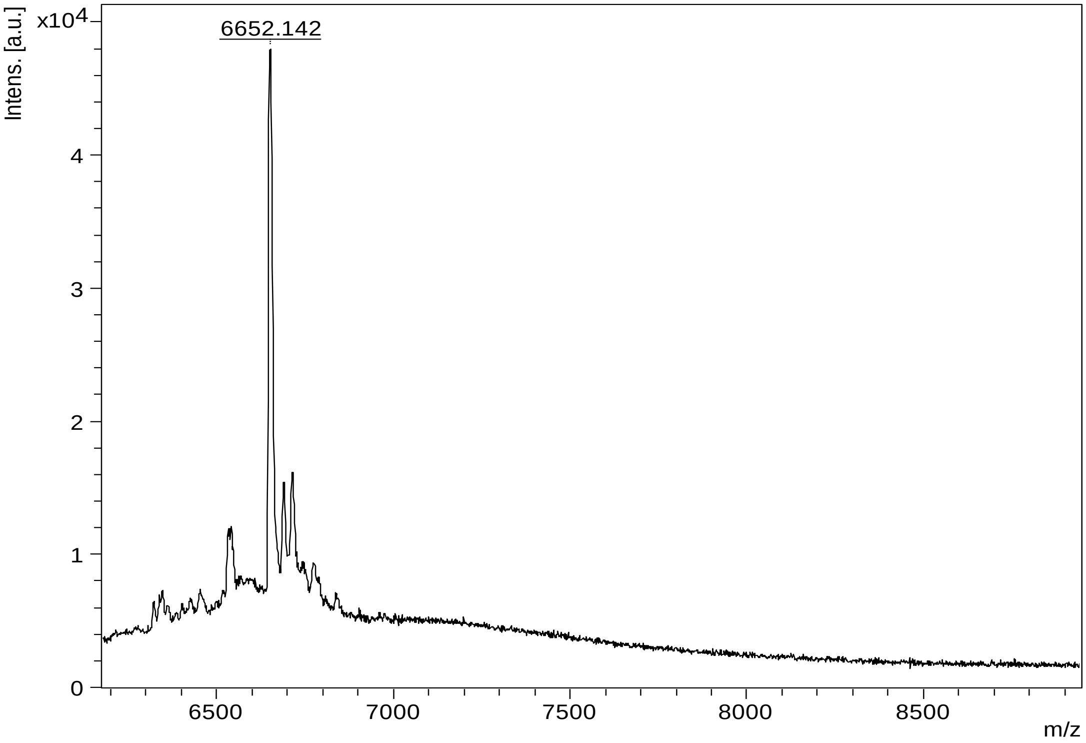

D. HPLC results for nucleoside standards and methods

Table 3: HPLC of Nucleoside Standards: Analysis was done on a Thermo Finnigan Surveyor HPLC with a Supelcosil LC-18-S column (250 x 4.6 mm) and a gradient of methanol in 0.1% formic acid (pH 4). [Data file: D:\Linda\Data\2022_April\Quad_std_mix2]

Nucleoside	Retention Time (minutes)	λmax
dU	13.11	262
5hmdU	13.11	264
5FdU	13.98	268
dC	15.14	279
dT	19.66	267
dG	22.23	254
dA	25.16	258

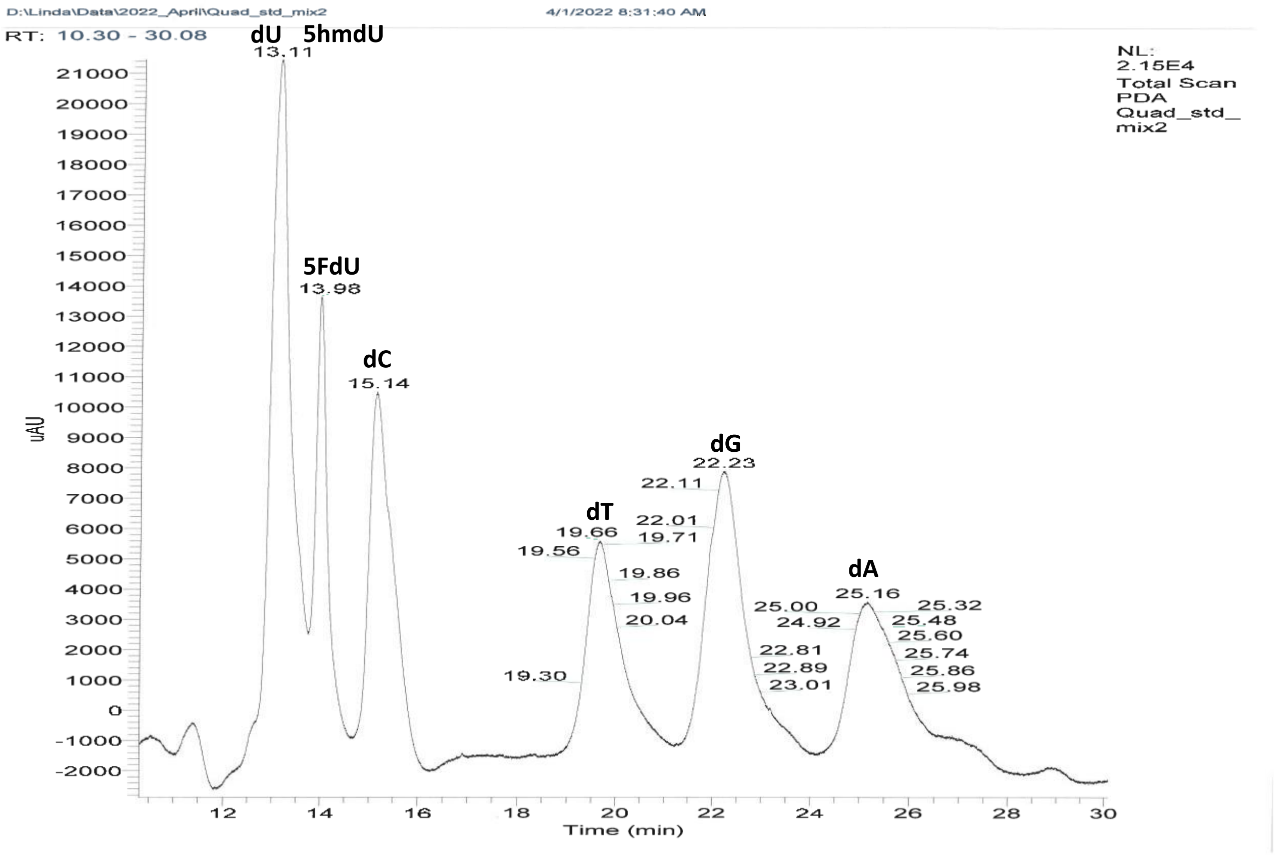

E. UV spectra from HPLC PDA for nucleoside standards

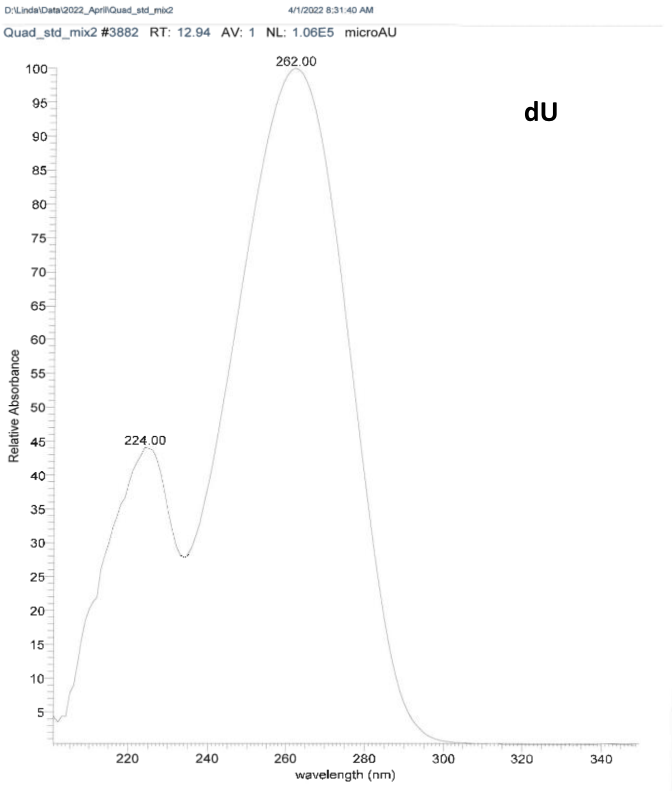

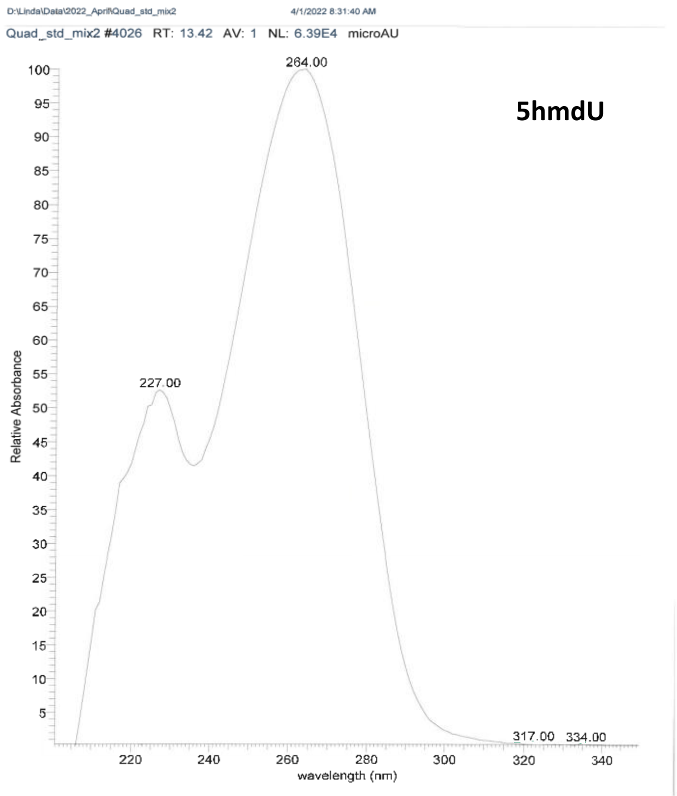

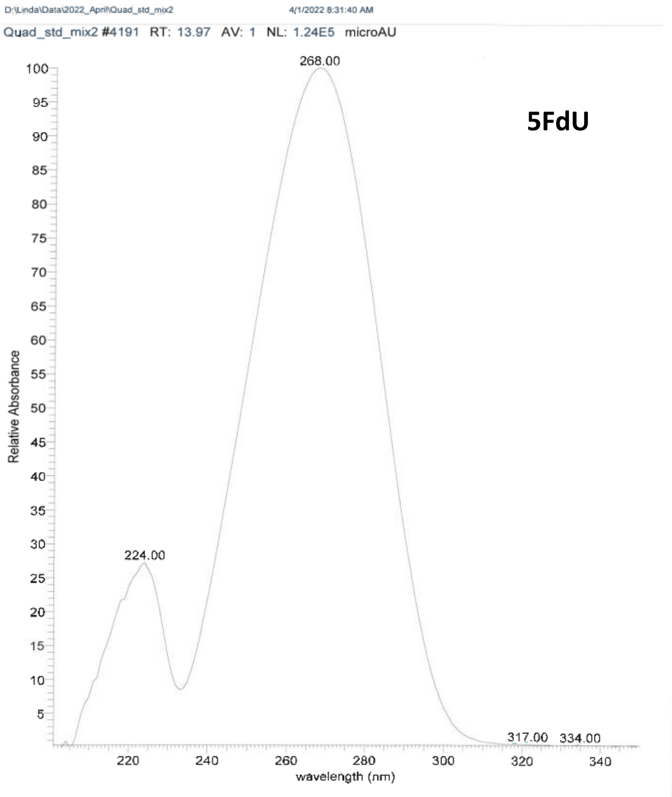

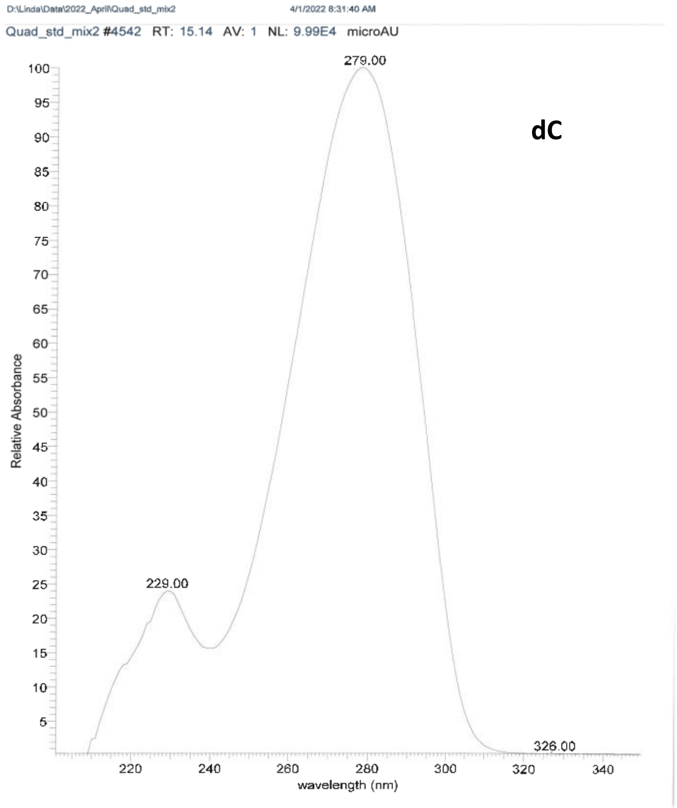

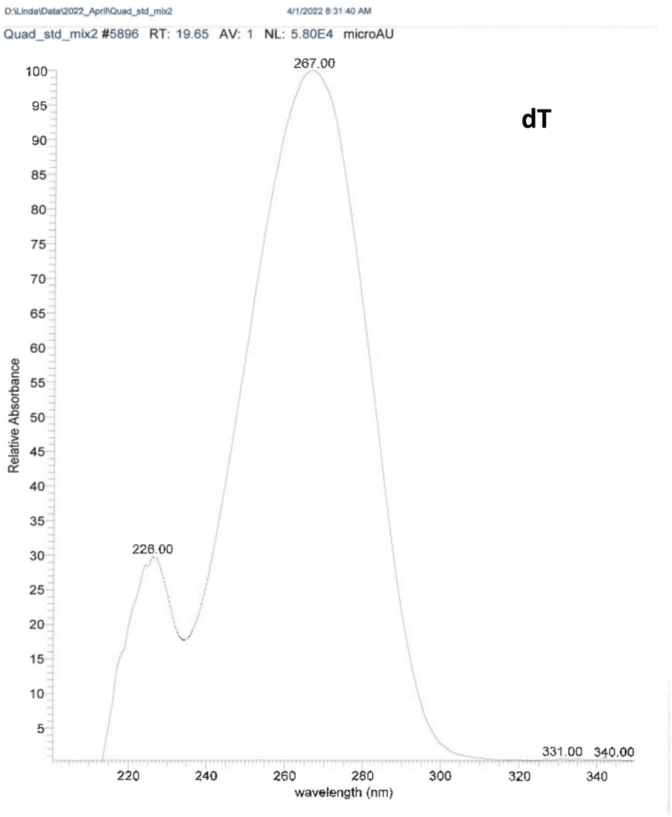
D:\Linda\Data\2022_April\Quad_std_mix2
4/1/2022 8:31:40 AM
Quad_std_mix2 #5896 RT: 19.65 AV: 1 NL: 5.80E4 microAU
dT
267.00
228.00
331.00
340.00
Relative Absorbance
wavelength (nm)

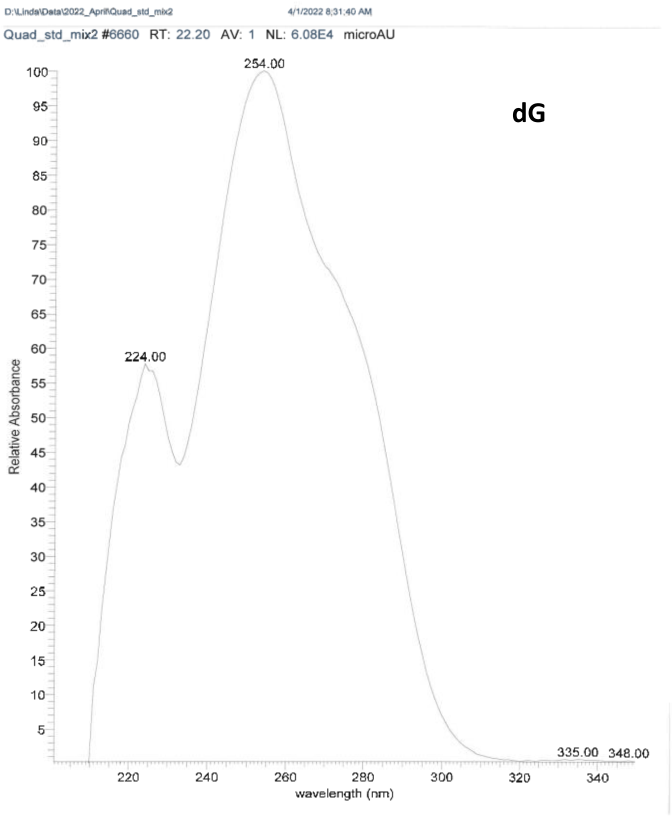
D:\Linda\Data\2022_April\Quad_std_mix2
4/1/2022 8:31:40 AM
Quad_std_mix2 #6660 RT: 22.20 AV: 1 NL: 6.08E4 microAU
dG
254.00
224.00
335.00
348.00
Relative Absorbance
wavelength (nm)

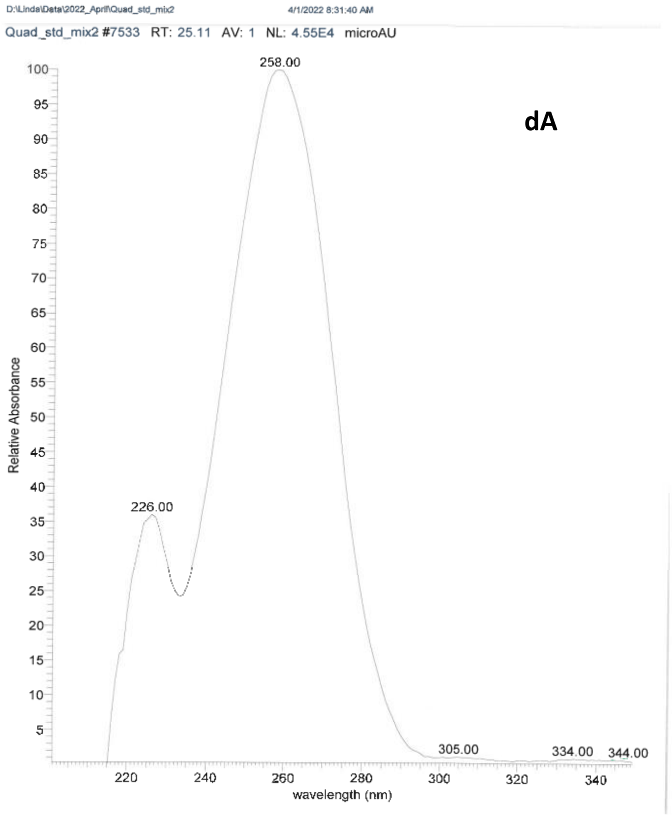
D:\Linda\Data\2022_April\Quad_std_mix2
4/1/2022 8:31:40 AM
Quad_std_mix2 #7533 RT: 25.11 AV: 1 NL: 4.55E4 microAU
dA
258.00
226.00
305.00
334.00
344.00
Relative Absorbance
wavelength (nm)

F. Enzyme digestion results for oligonucleotides and methods

Table 4: Enzyme Digestion and HPLC Analysis of 18mer Oligonucleotides (see Table 1 for oligonucleotide sequence)

Oligo	Nucleoside	RT (minutes)	λmax	Data File (D:\Linda\Data)
Quad-22mer-U	dU	10.59	262	\2022_March\L-9123
Quad-22mer-5FU	5FdU	11.63	269	\2022_April\L-9143
Quad-22mer-5hmU	5hmdU	14.68	265	\2022_April\L-1051_220401142442
Quad-22mer-U-FAM	dU	11.98	261	\2022_March\L-9113
Quad-22mer-5FU-FAM	5FdU	17.29	269	\2022_April\L-1011
Quad-22mer-5hmU-FAM	5hmdU	13.08	263	\2022_April\L-1041_220401133628
Quad-22mer-U-FAM-BHQ1	dU	13.02	261	\2022_March\L-9118
Quad-22mer-5FU-FAM-BHQ1	5FdU	16.70	269	\2022_April\L-1016
Quad-22mer-5hmU-FAM-BHQ1	5hmdU	16.25	264	\2022_April\L-1046
Quad-22mer-U-Comp	dU	12.29	262	\2022_March\L-9128
Quad-22mer-U-Comp-Cy5	dU	6.44	262	\2022_April\L-1131_220427094628
22mer-nonquad_5hmU	5hmdU	15.99	264	\2022_April\L-1154
22mer-nonquad_U	dU	6.6	261	\2022_April\L-1121

Enzyme Digestion: Per 1 OD oligo, water (36 µL) and nuclease P1 buffer (4 µL) were added. Nuclease P1 (0.4 µL) was added and the mixture heated to 37°C for 1 hour. Water (7.2 µL) was added along with bacterial alkaline phosphatase buffer (0.8 µL). Bacterial alkaline phosphatase (0.2 µL) was added and the mixture was heated to 37°C for 1 hour. An additional 18.07 µL of water was added and 25 µL was injected on the Thermo Finnigan Surveyor HPLC using a Supelcosil LC-18-S column (250 x 4.6 mm) and a gradient of methanol in 0.1% formic acid, pH 4.

Nuclease P1 (New England BioLabs M0660S): Storage buffer: 2 mM Tris-HCl, 50 mM NaCl, 1 mM $ZnCl_2$, 50% glycerol, pH 7.2; 10x Reaction buffer: 500 mM sodium acetate pH 5.5

Bacterial alkaline phosphatase (Invitrogen 18011-015): Storage buffer: 10 mM Tris-HCl, pH 8, 120 mM NaCl, 50% (v/v) glycerol; 10x Reaction buffer: 100 mM Tris-HCl, pH 8.0

Reference:

G1. Fujimoto, J.; Tran, L. and Sowers, L. C. (1997) Synthesis and cleavage of oligodeoxynucleotides containing 5-hydroxyuracil residue at a defined site. *Chem. Res. Toxicol*. **10**, 1254-1258. DOI: 10.1021/TX970102B PMID: 9403179

G. HPLC and UV spectra for enzyme digestion of oligonucleotides

Quad-22mer-U

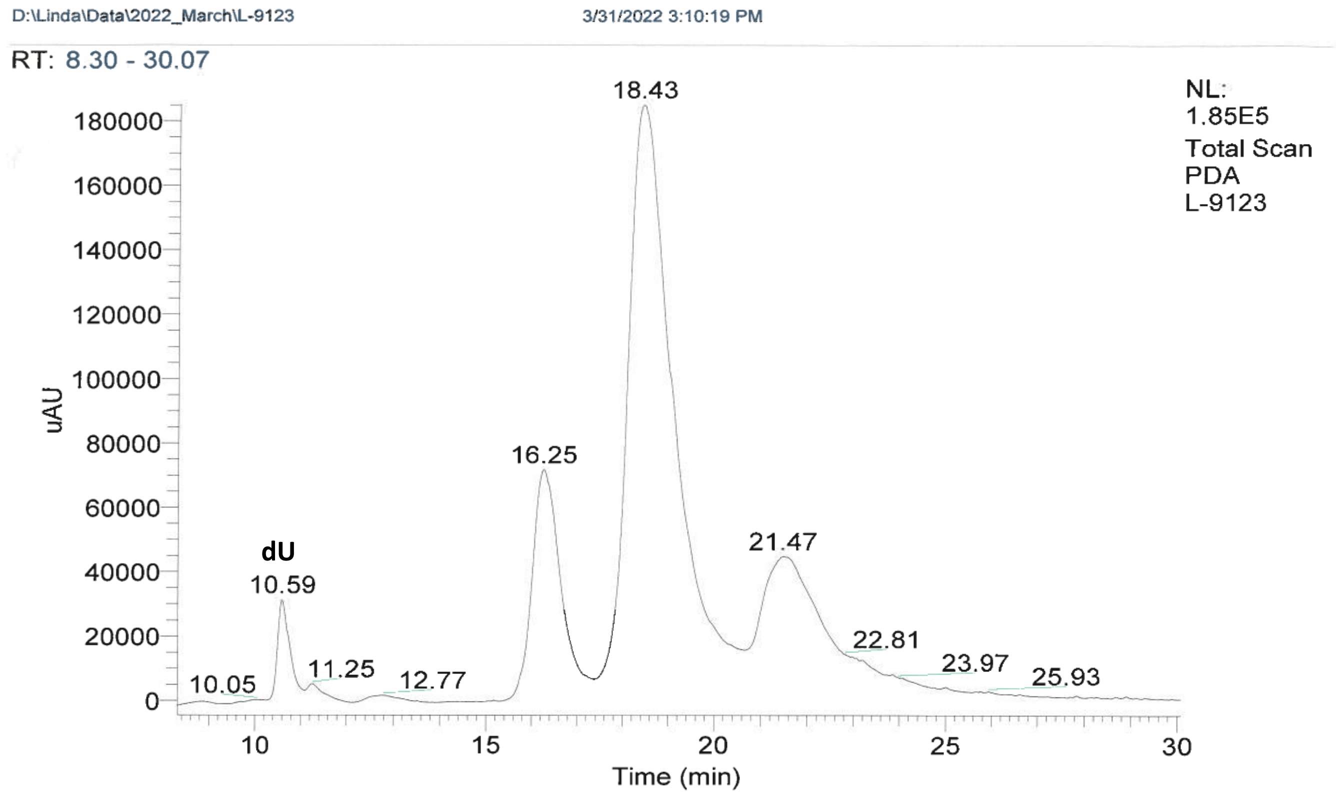

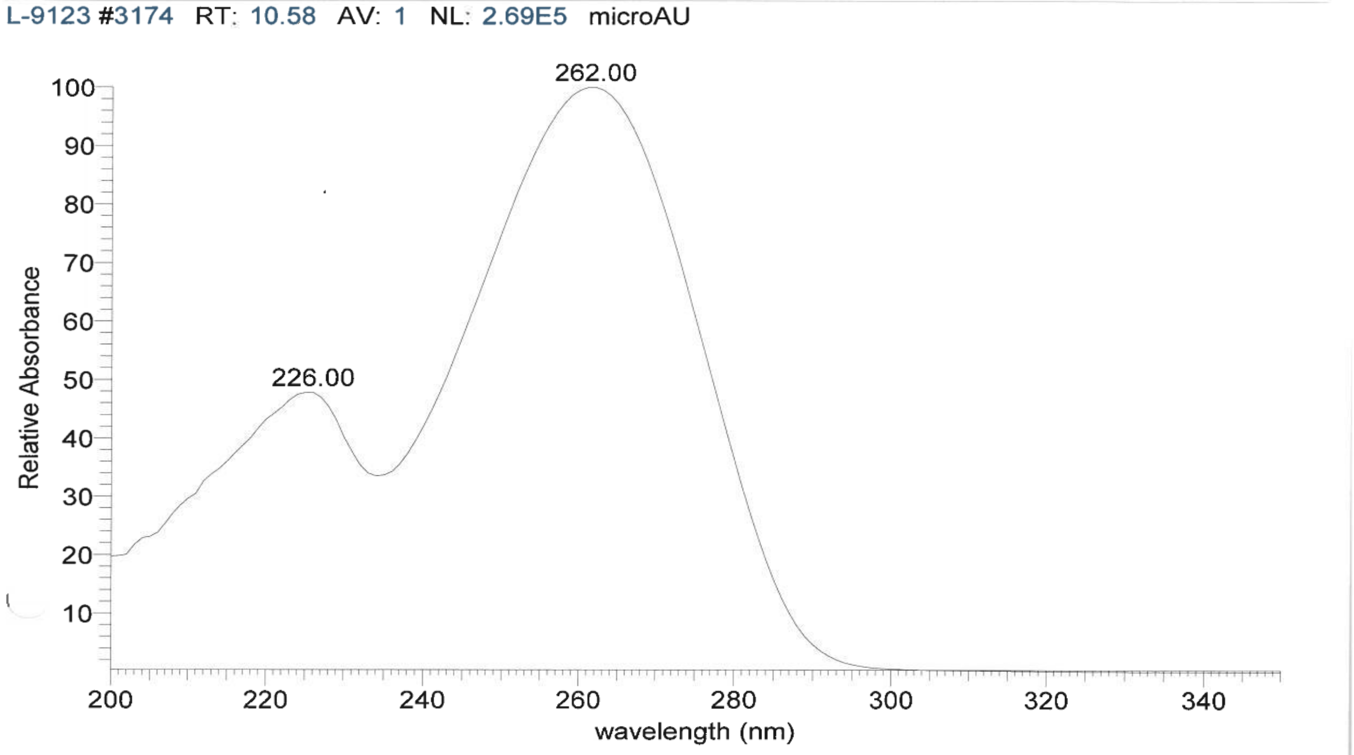

Quad-22mer-5FU

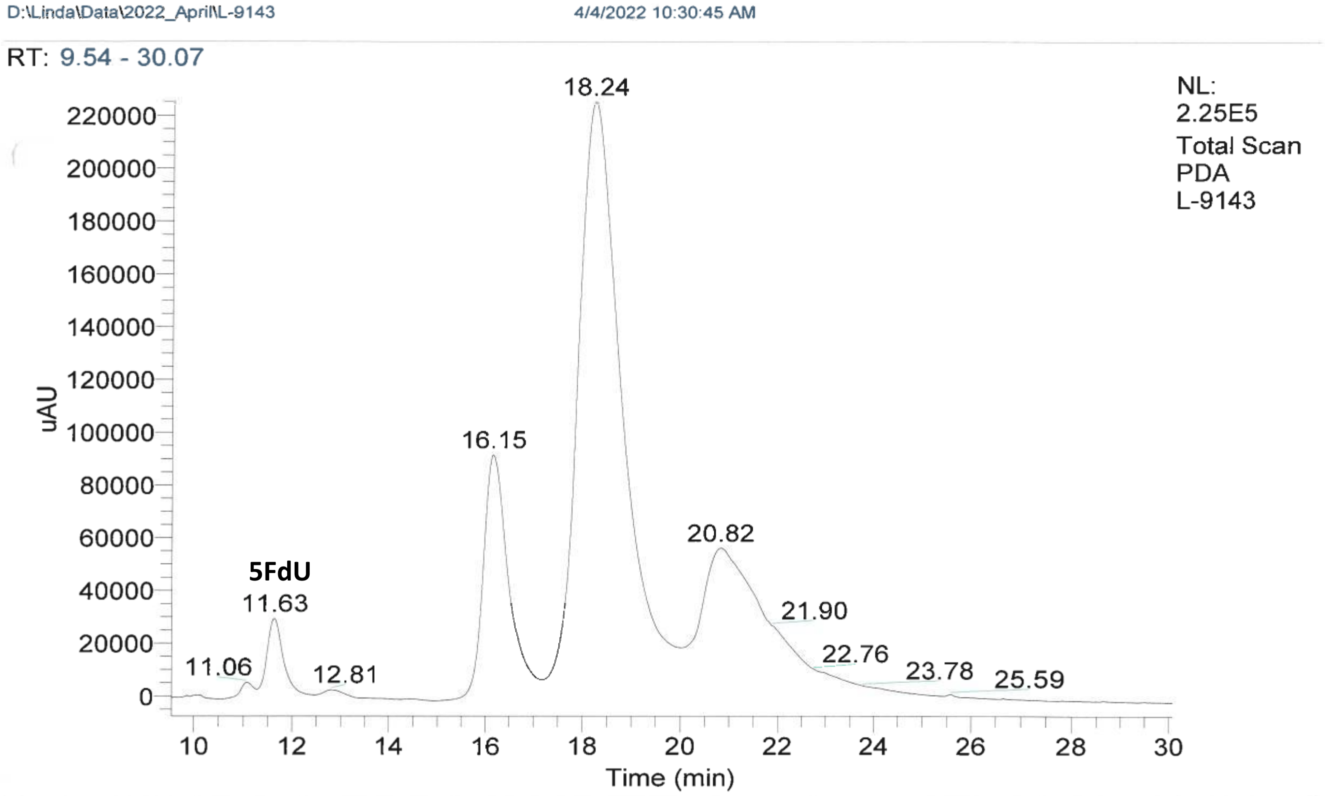

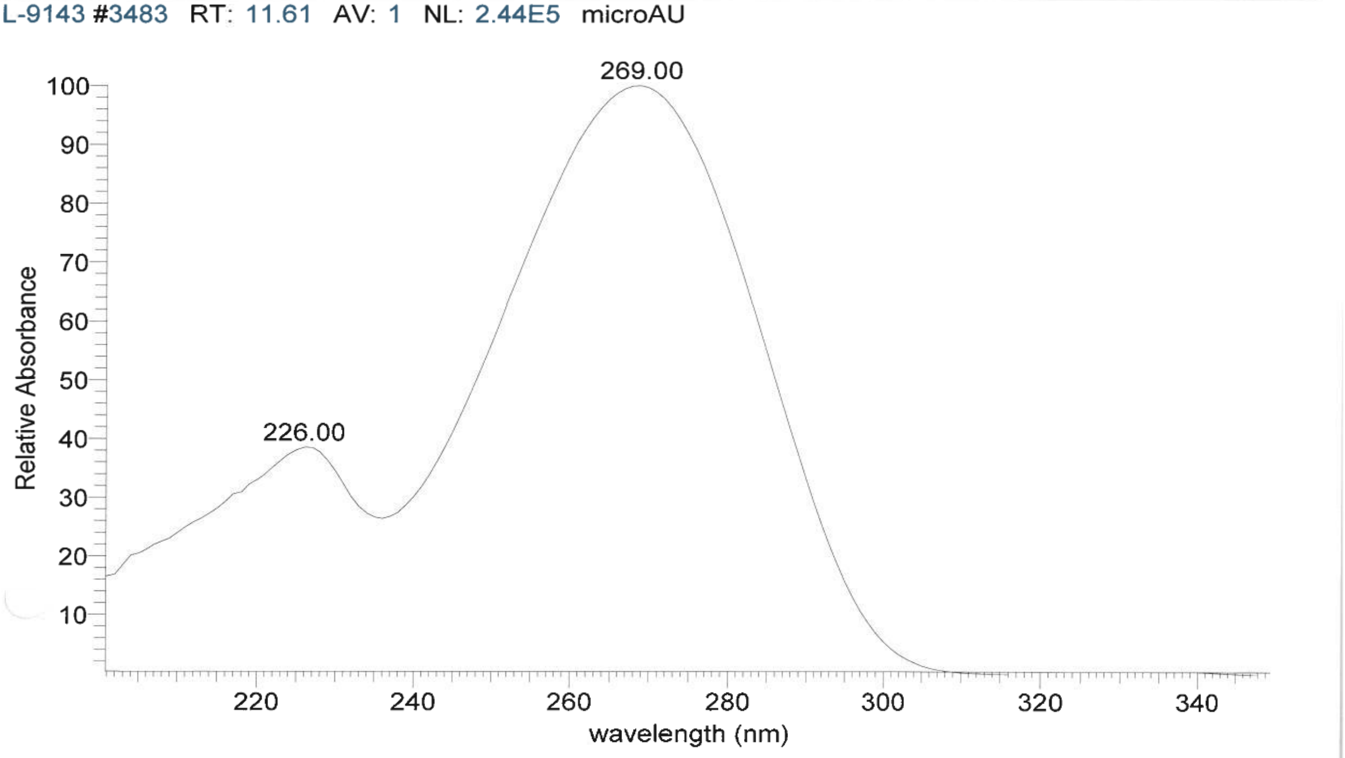

Quad-22mer-5hmU

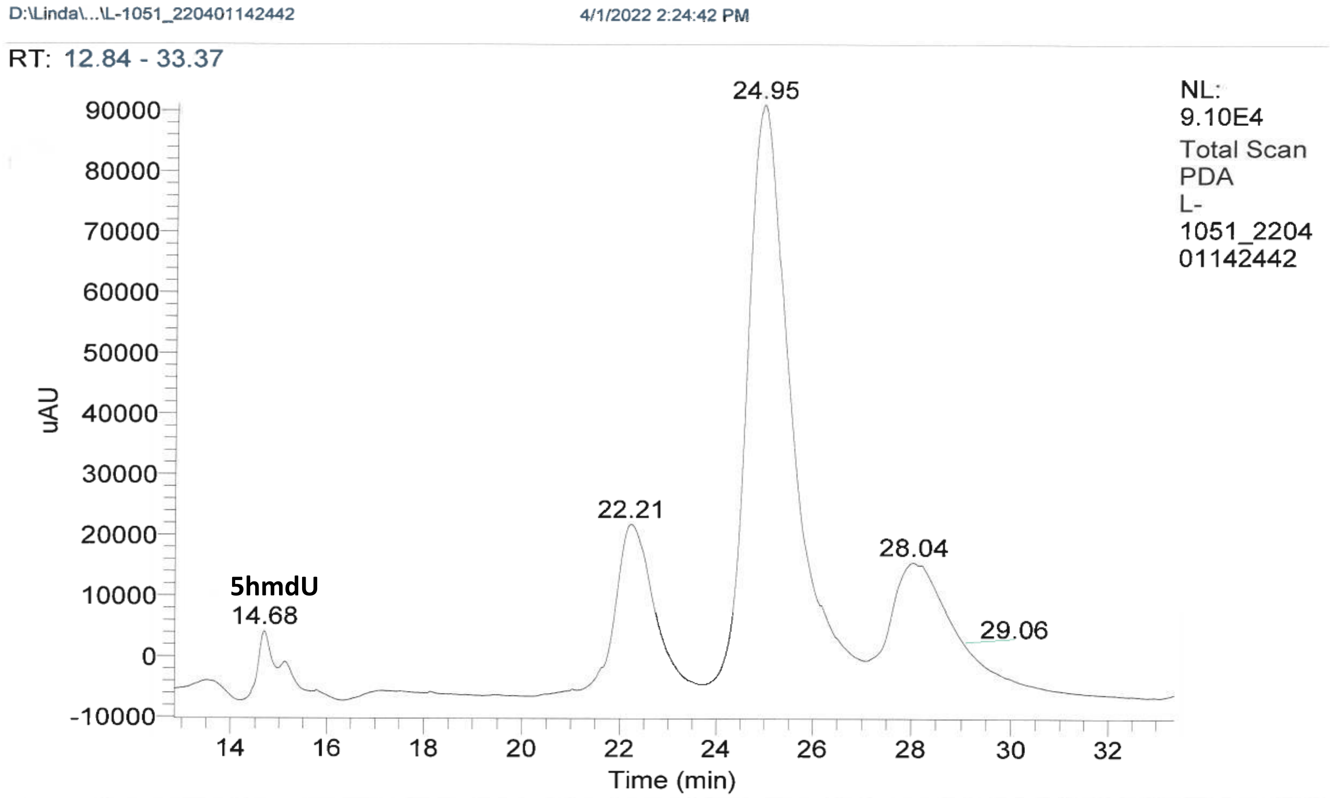

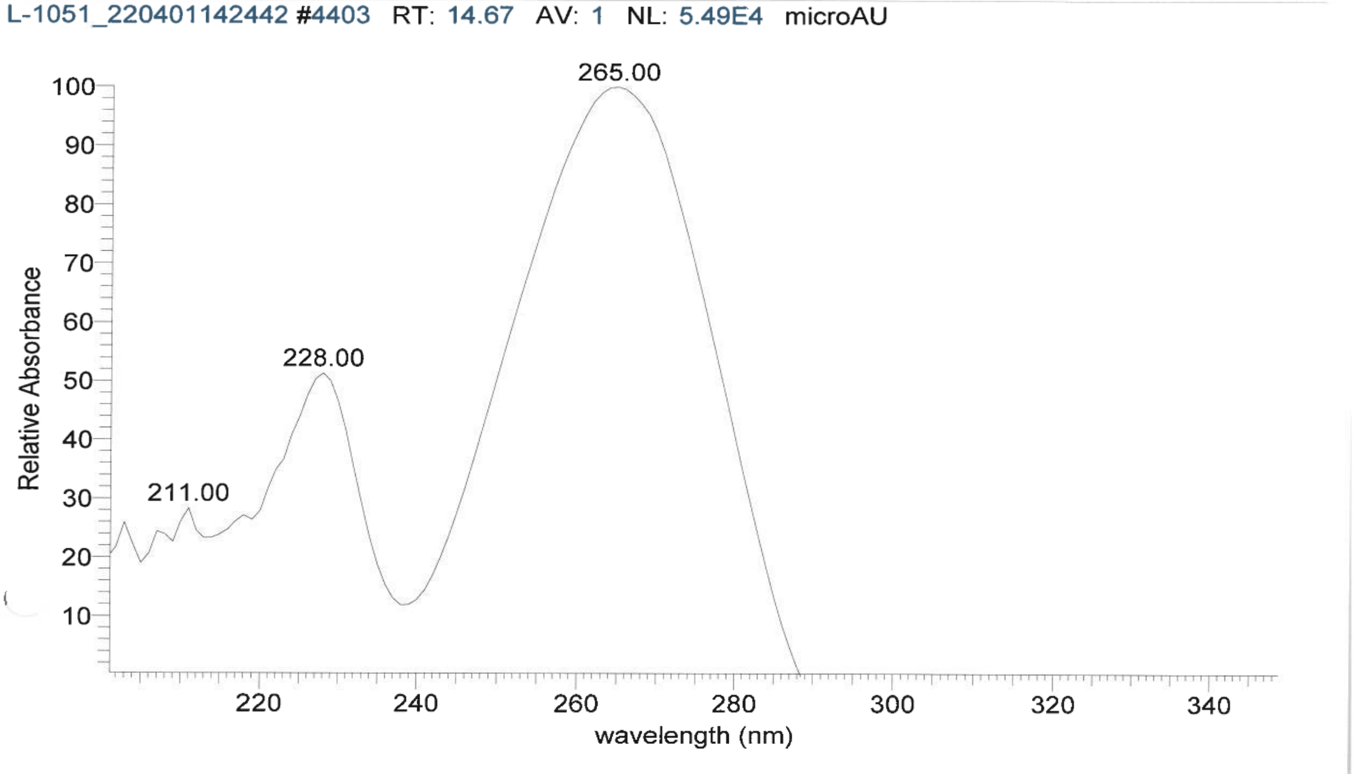

Quad-22mer-U-FAM

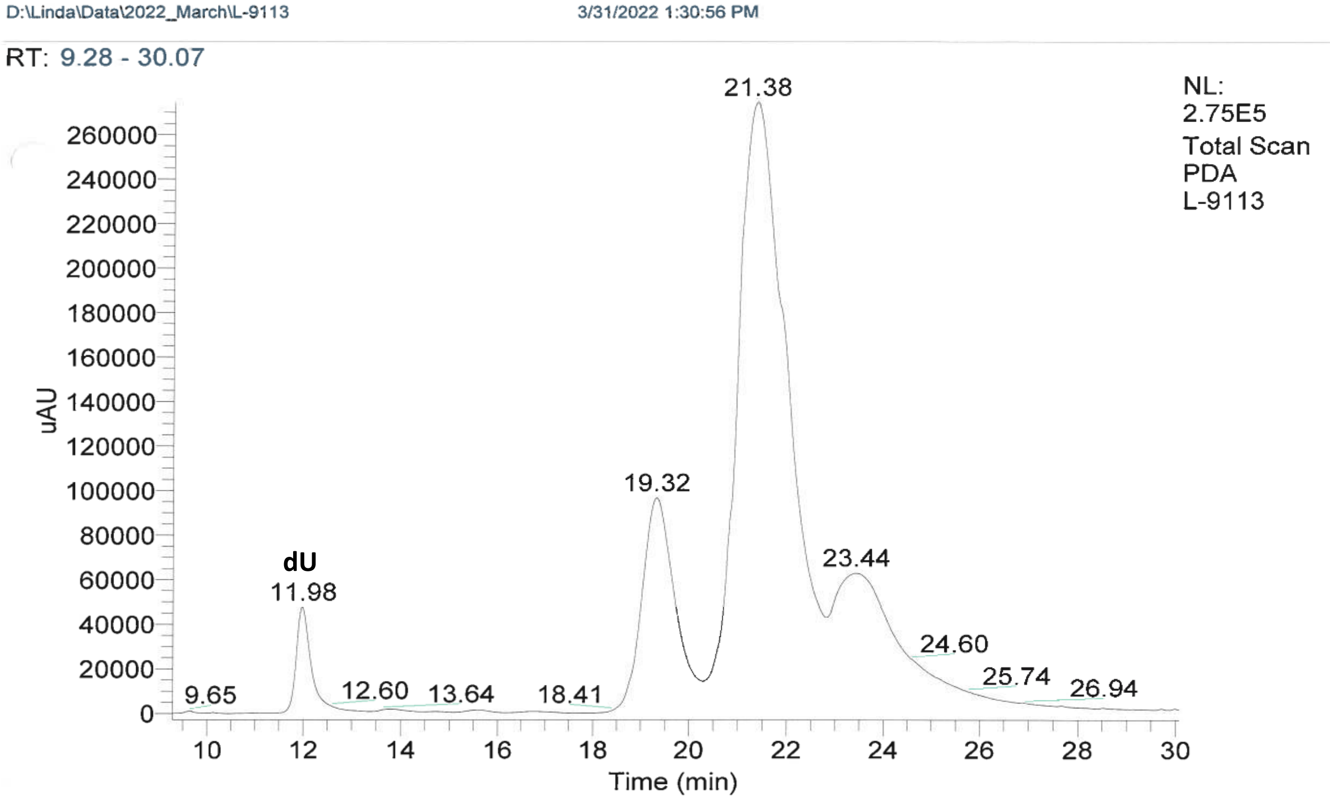

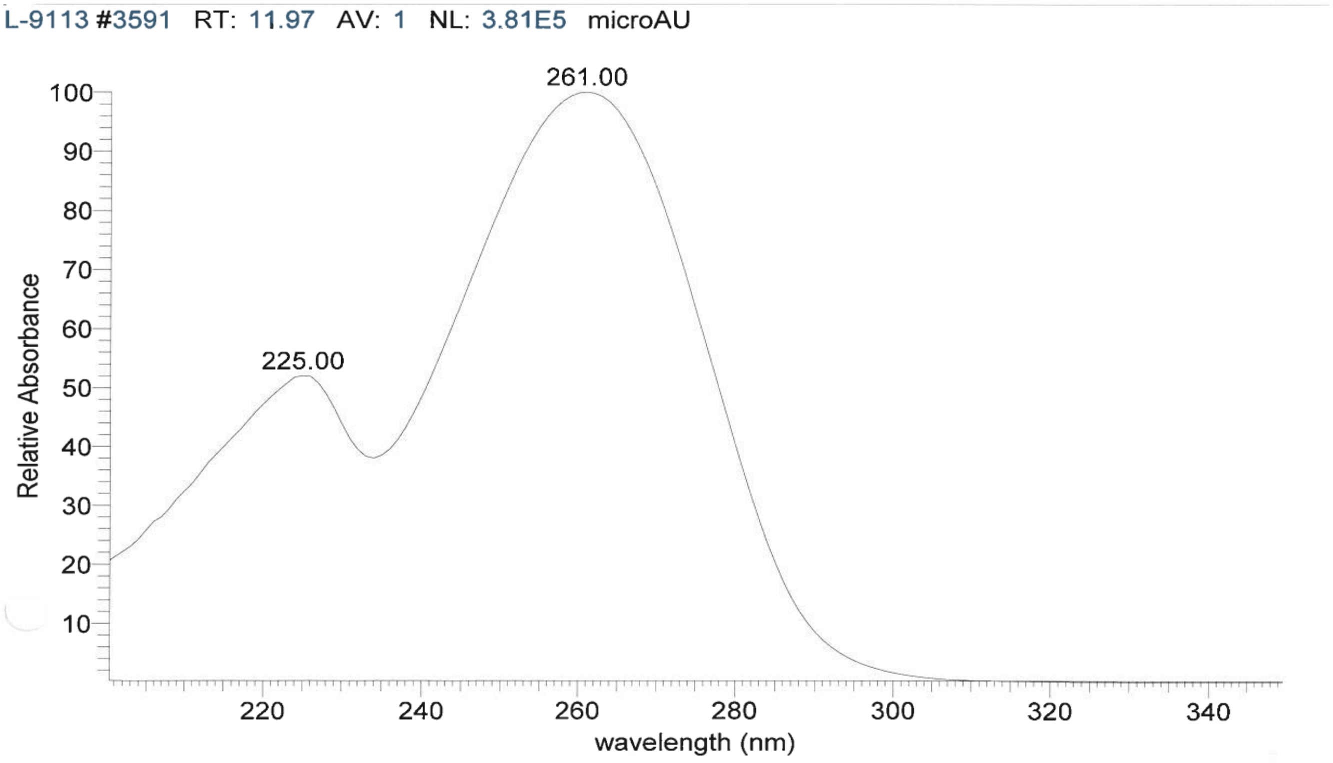

Quad-22mer-5FU-FAM

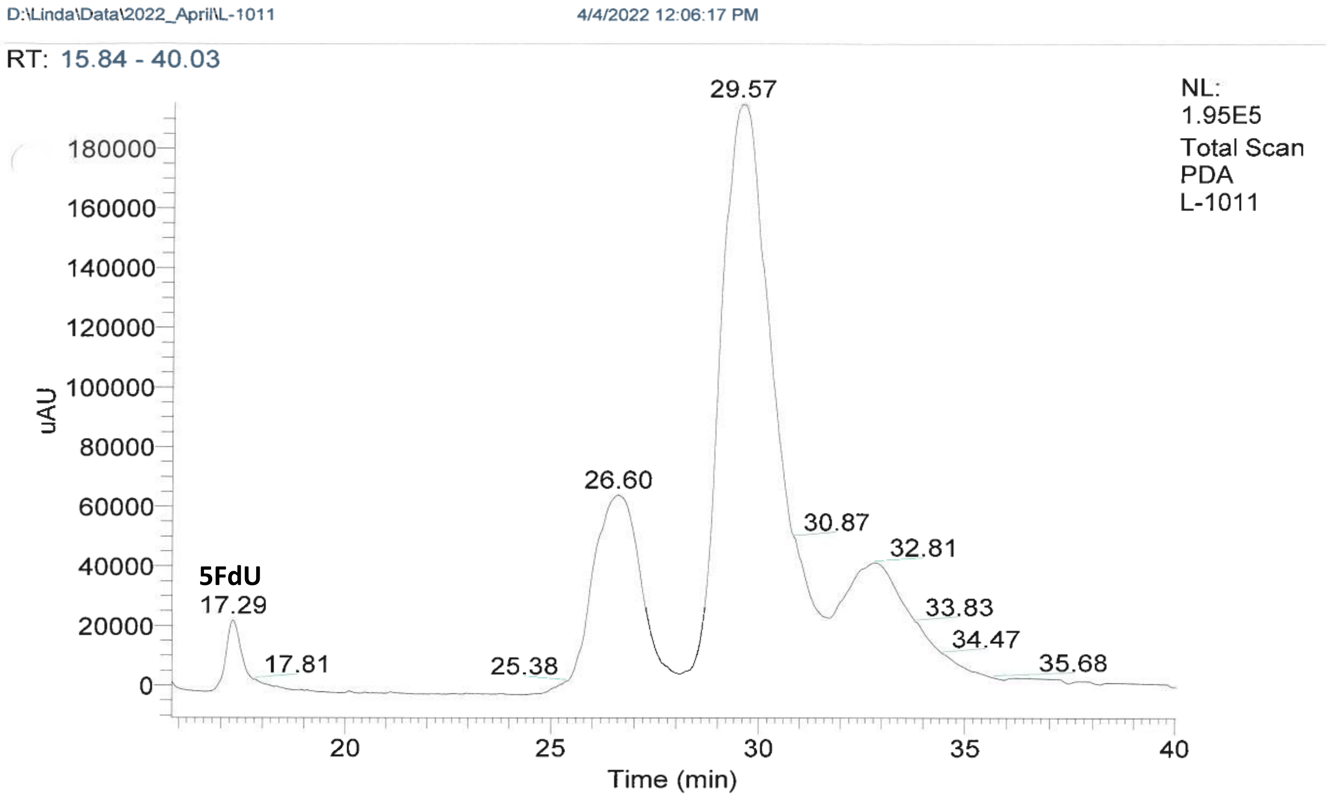

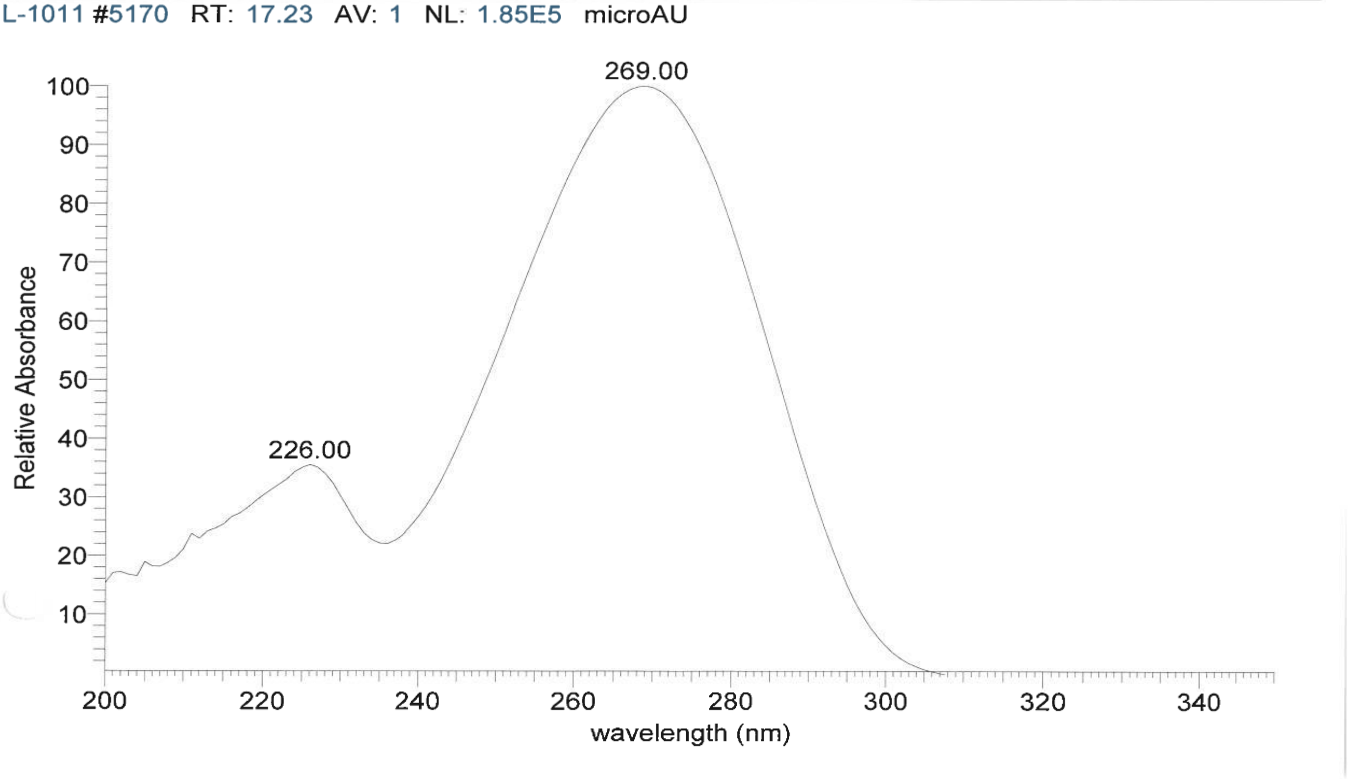

Quad-22mer-5hmU-FAM

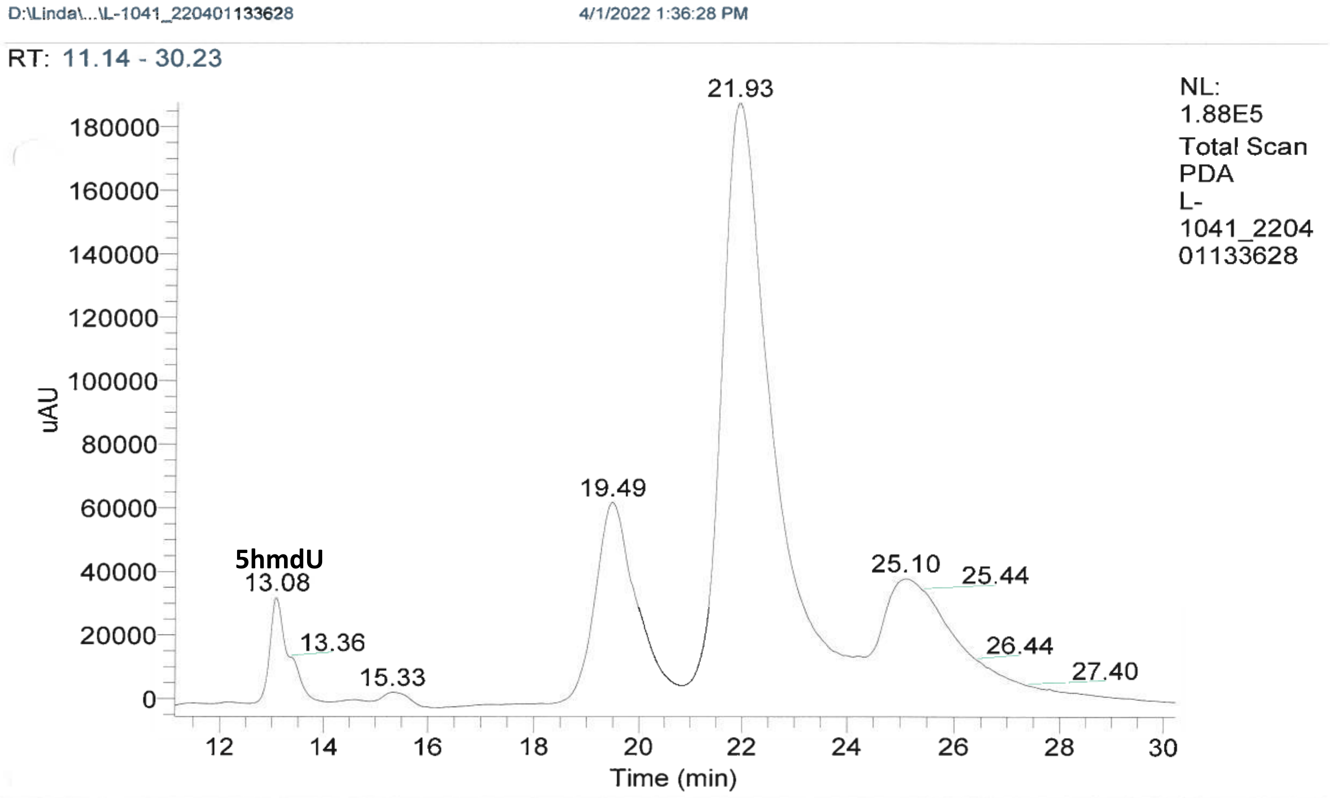

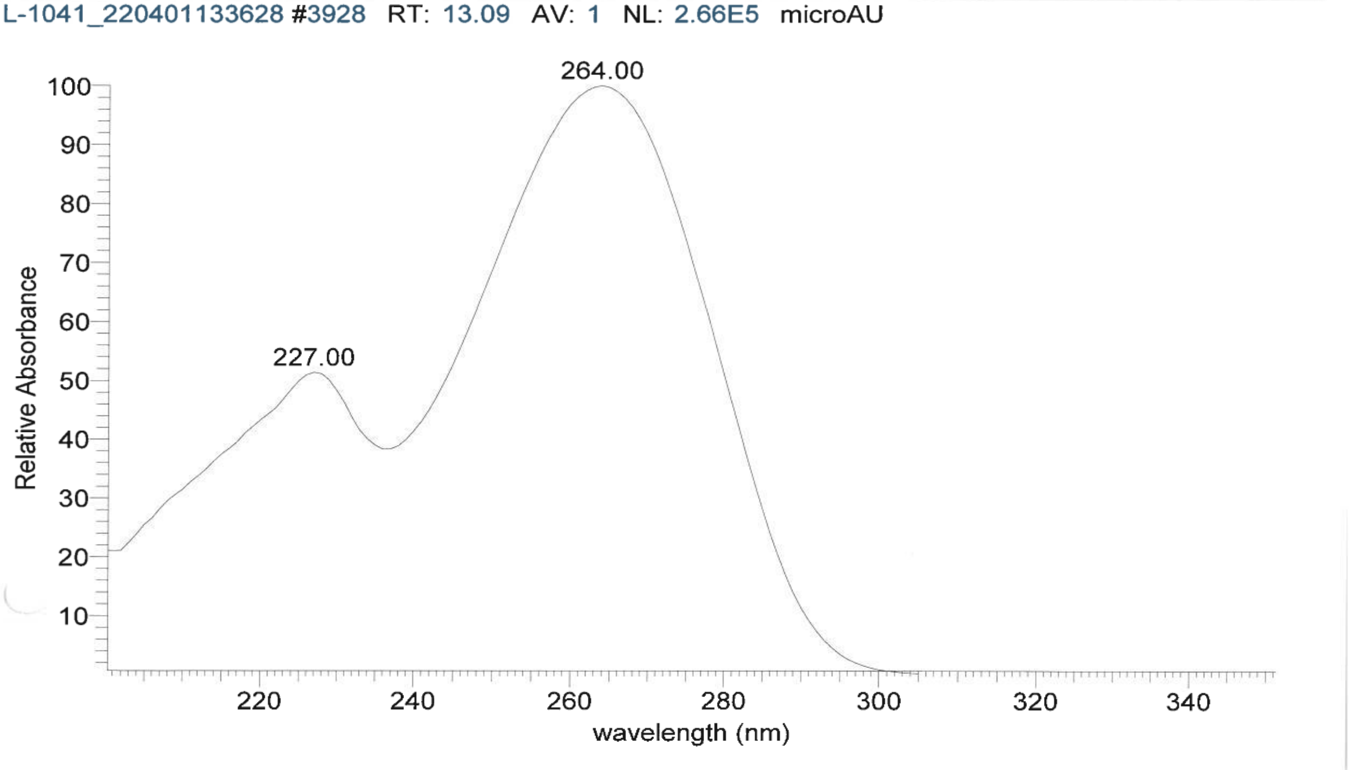

Quad-22mer-U-FAM-BHQ1

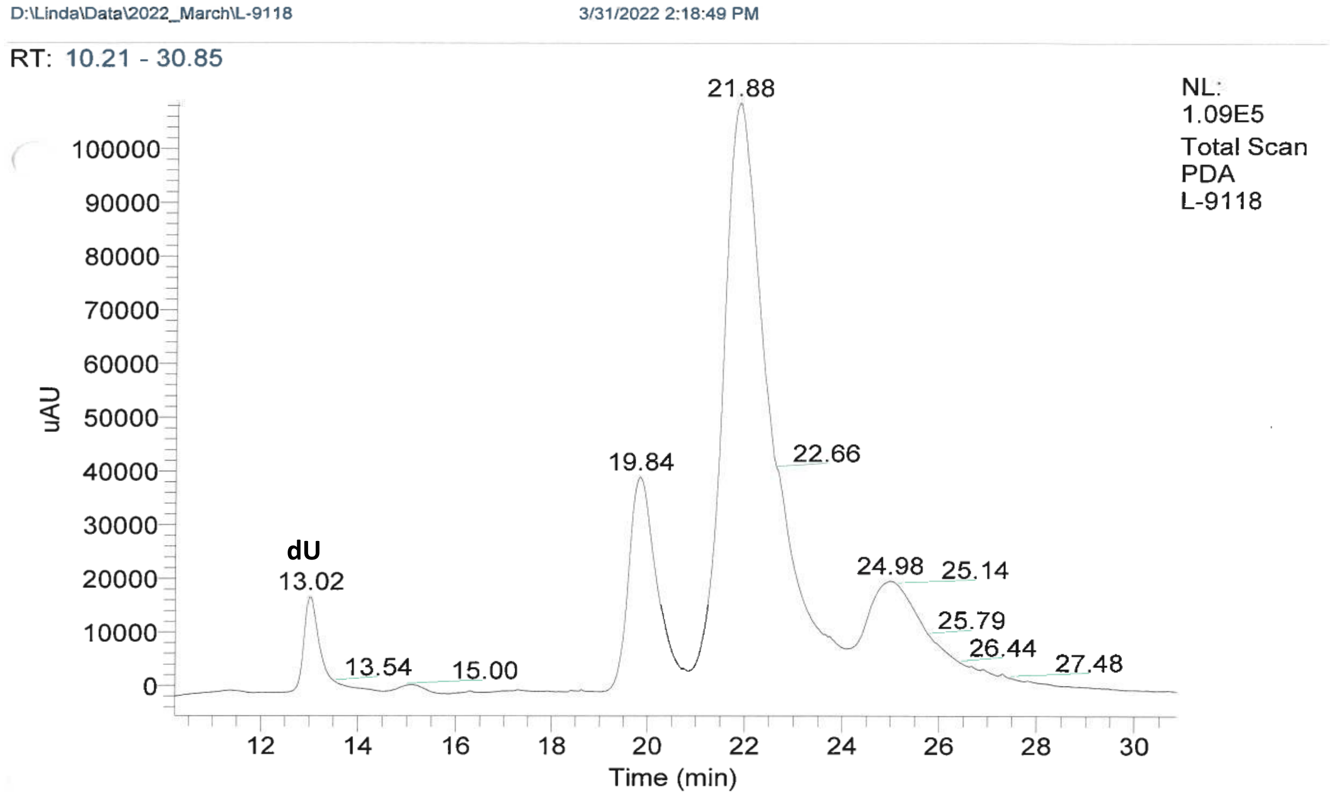

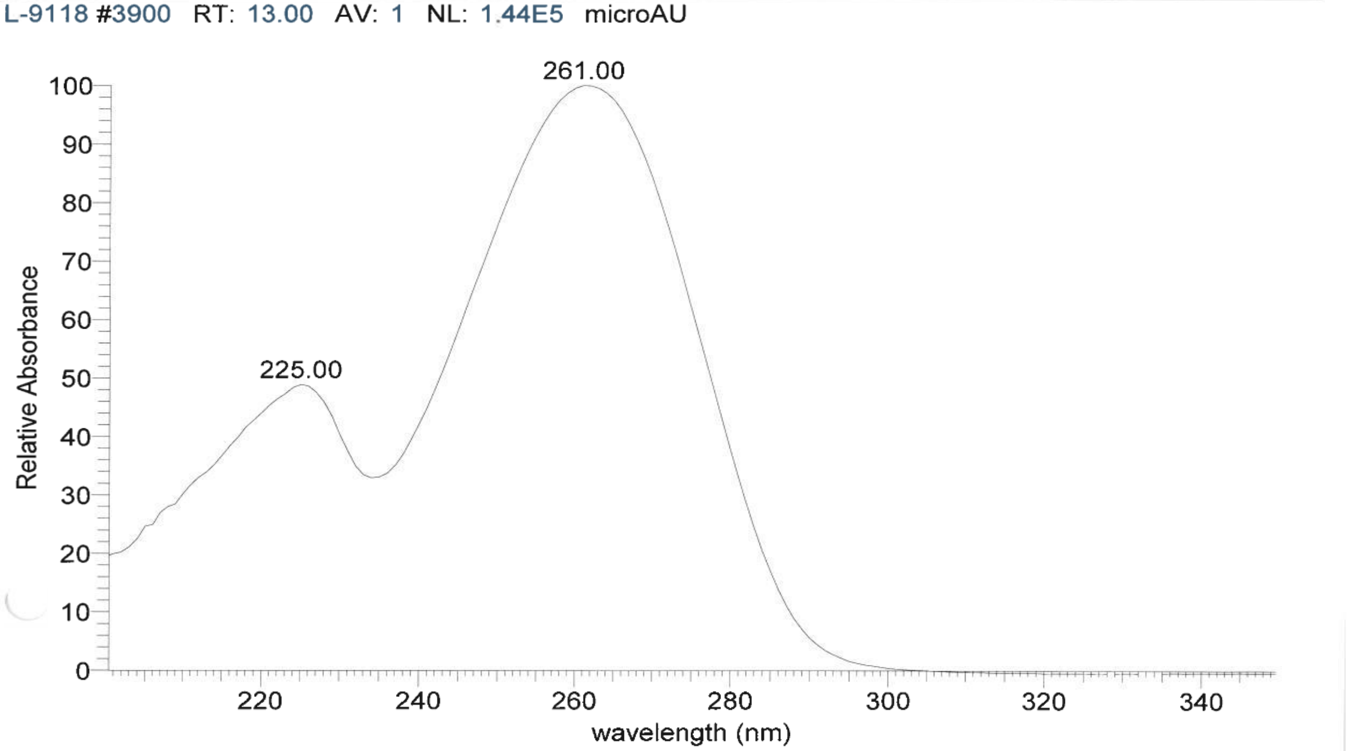

Quad-22mer-5FU-FAM-BHQ1

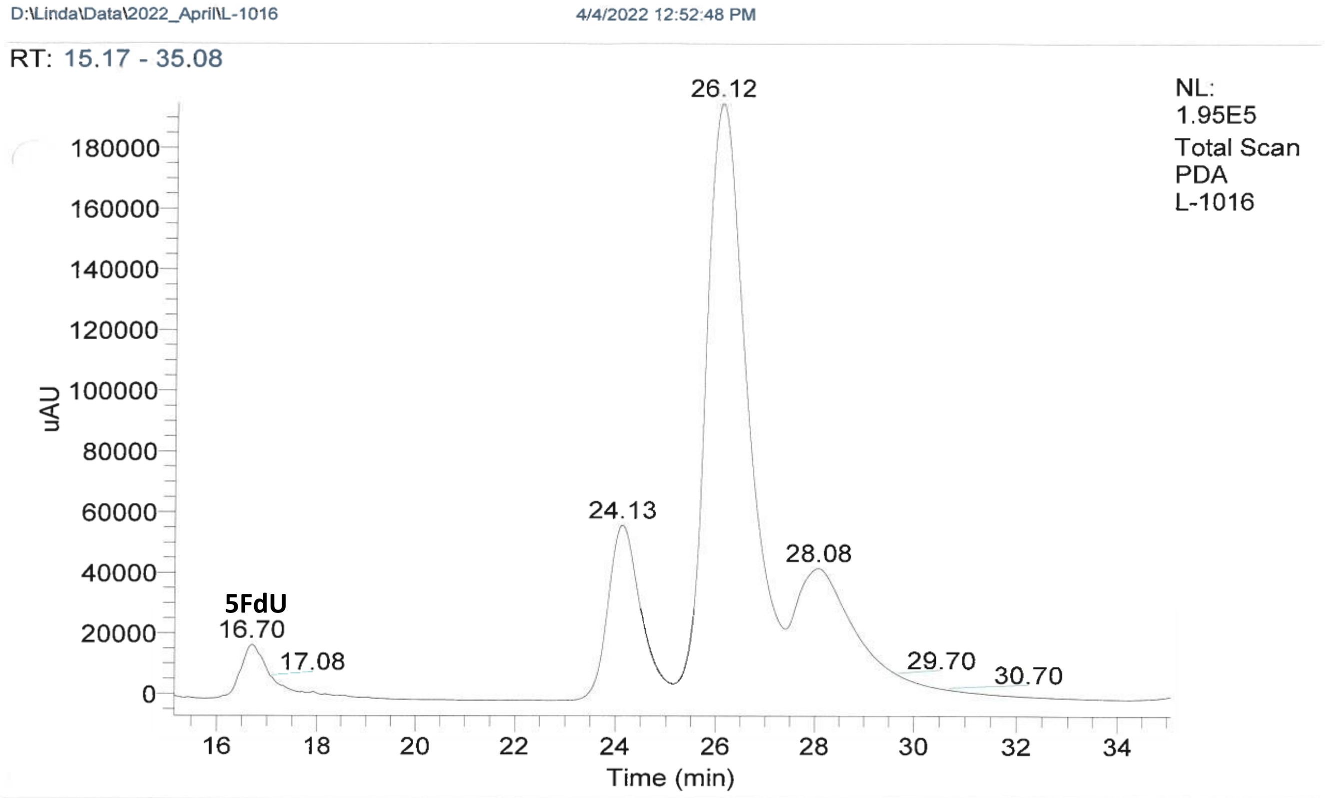

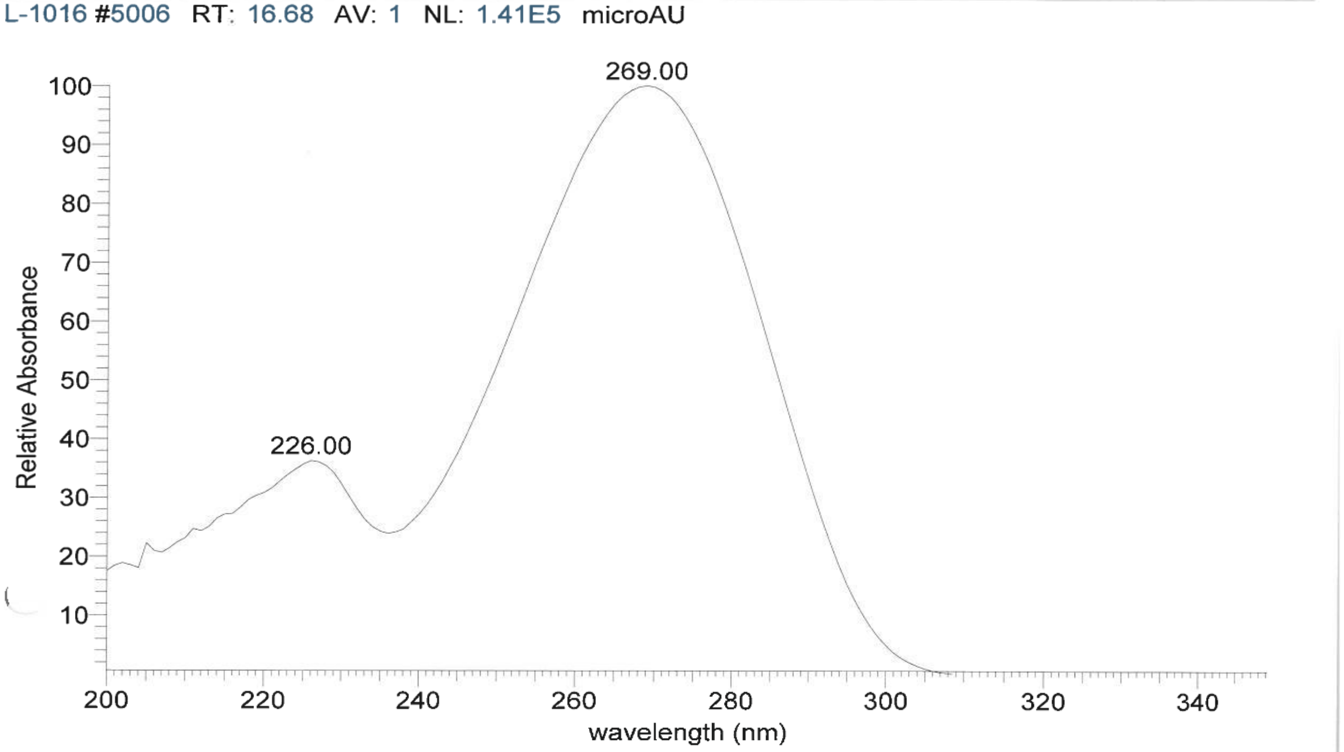

Quad-22mer-5hmU-FAM-BHQ1

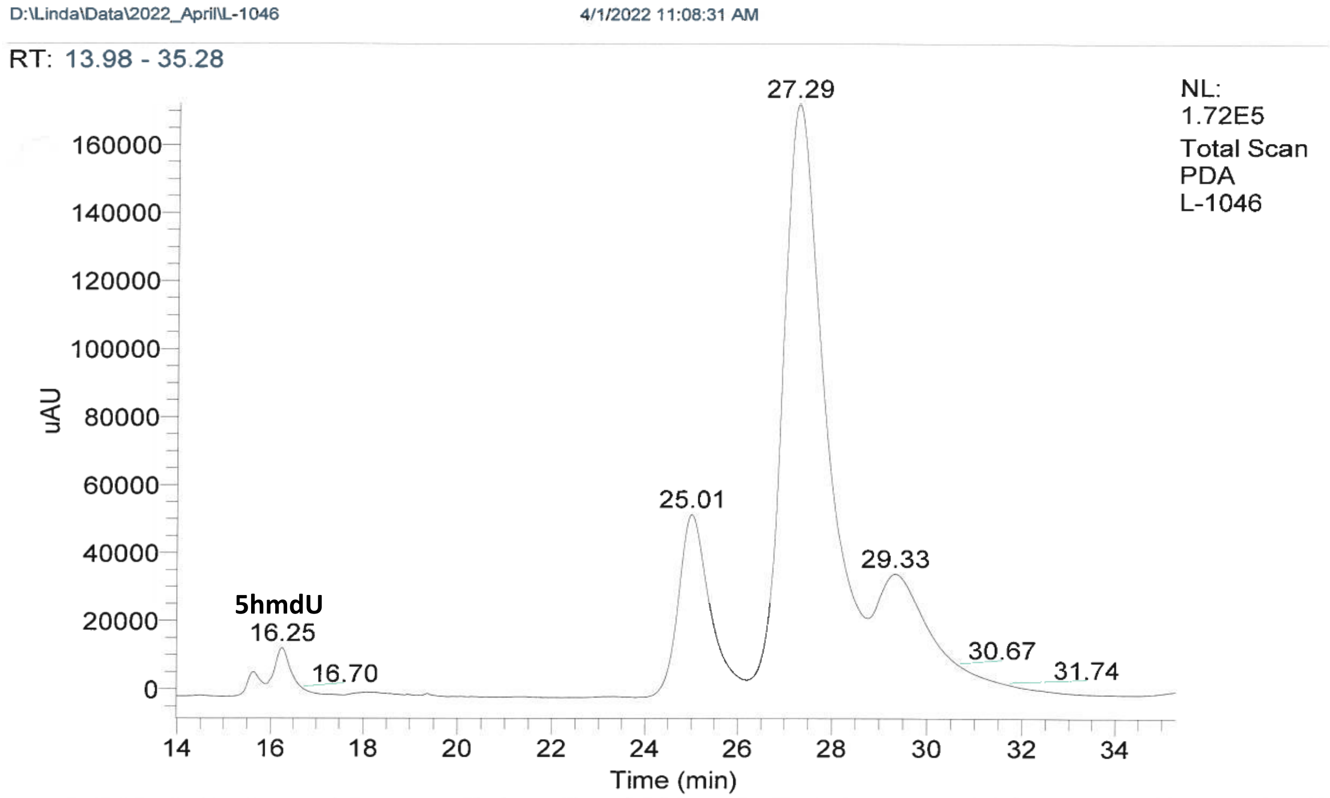

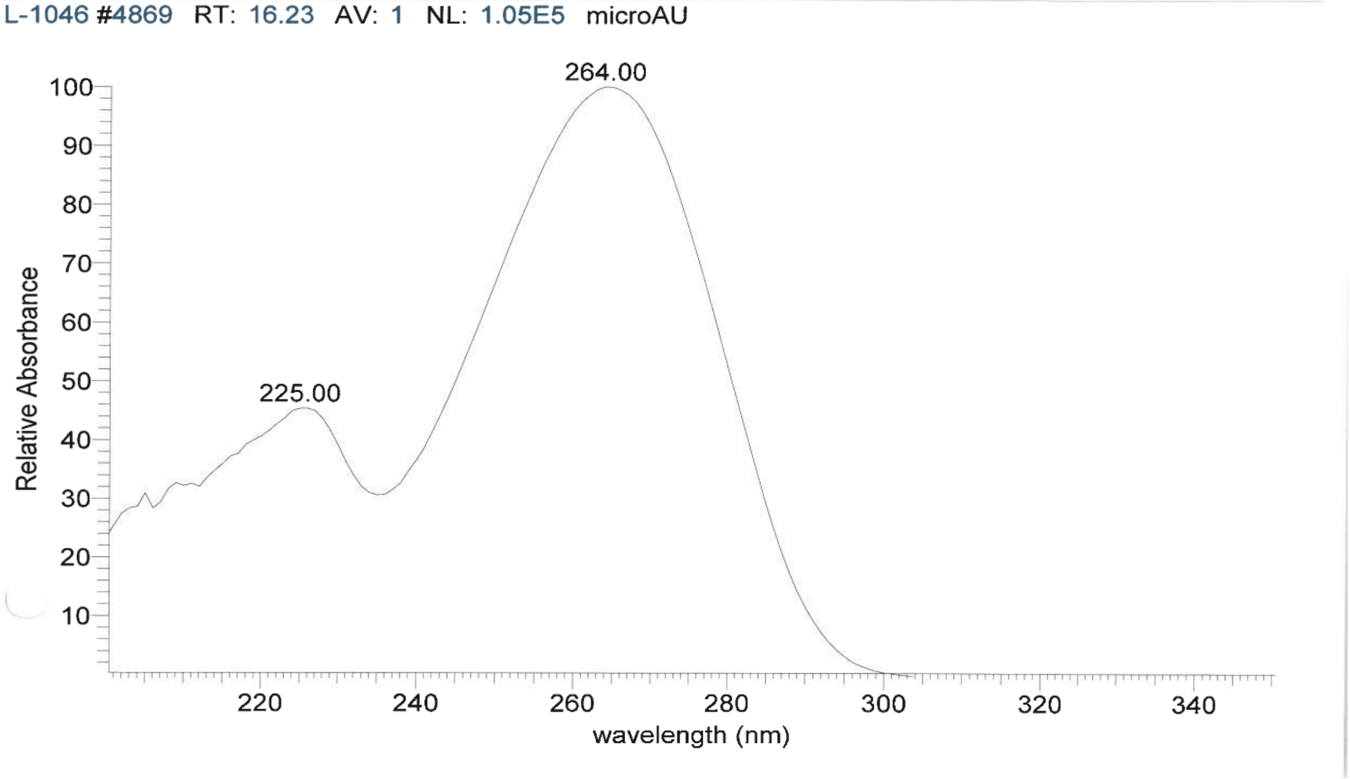

Quad-22mer-U-Comp

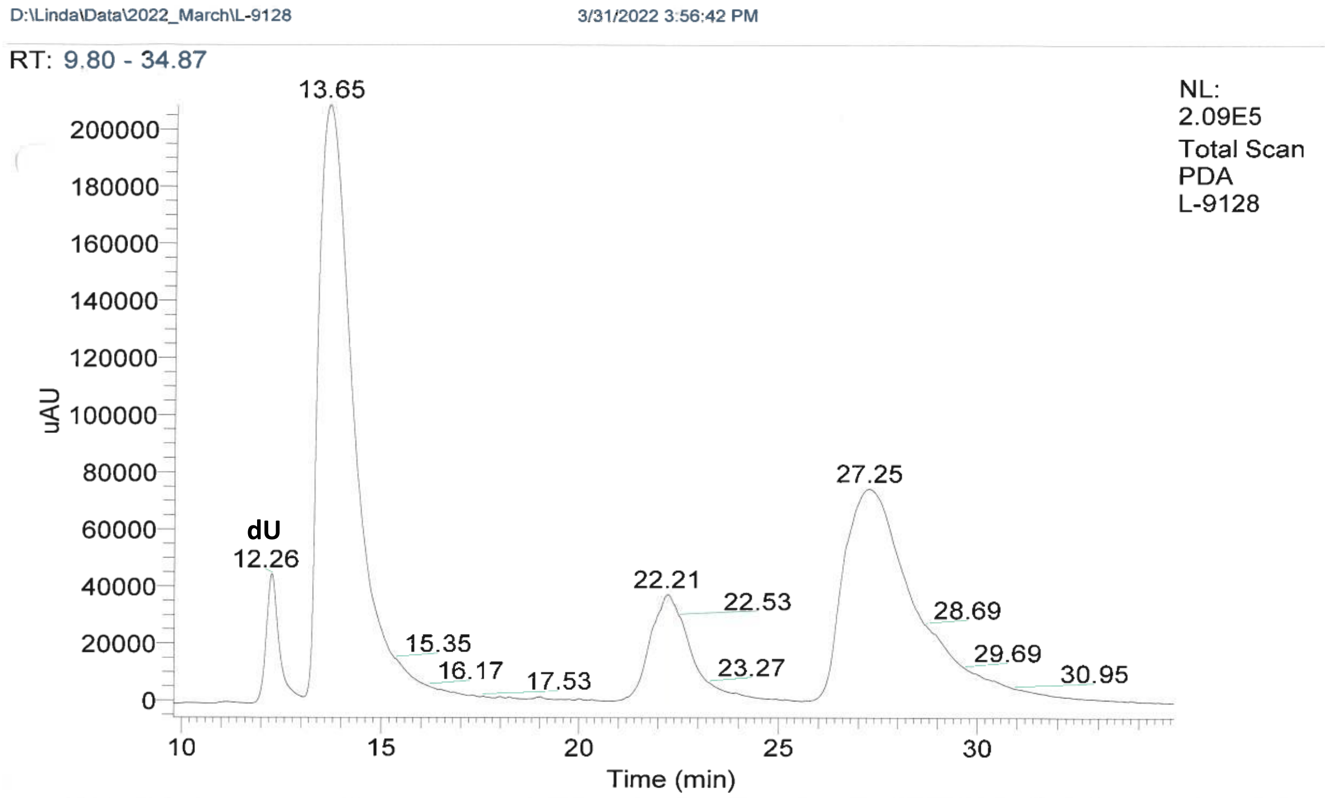

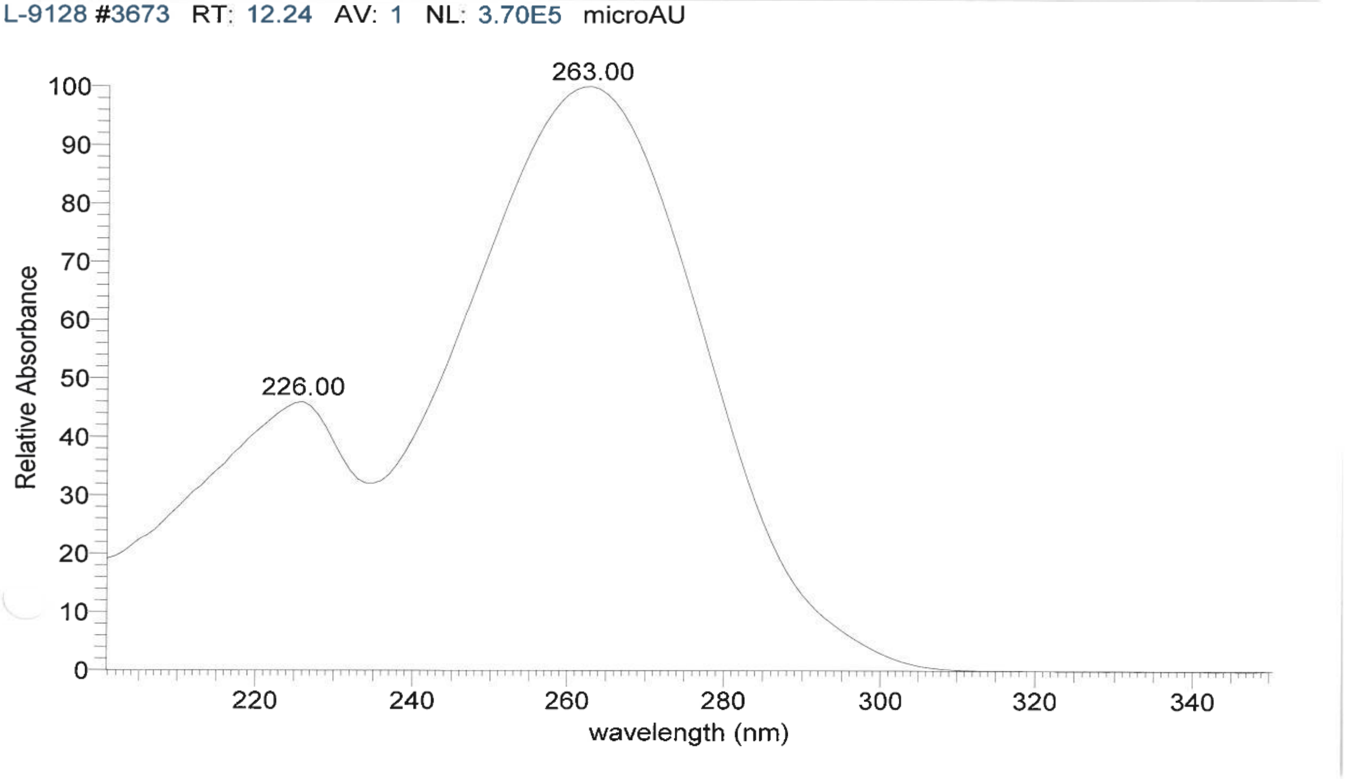

Quad-22mer-U-Comp-Cy5

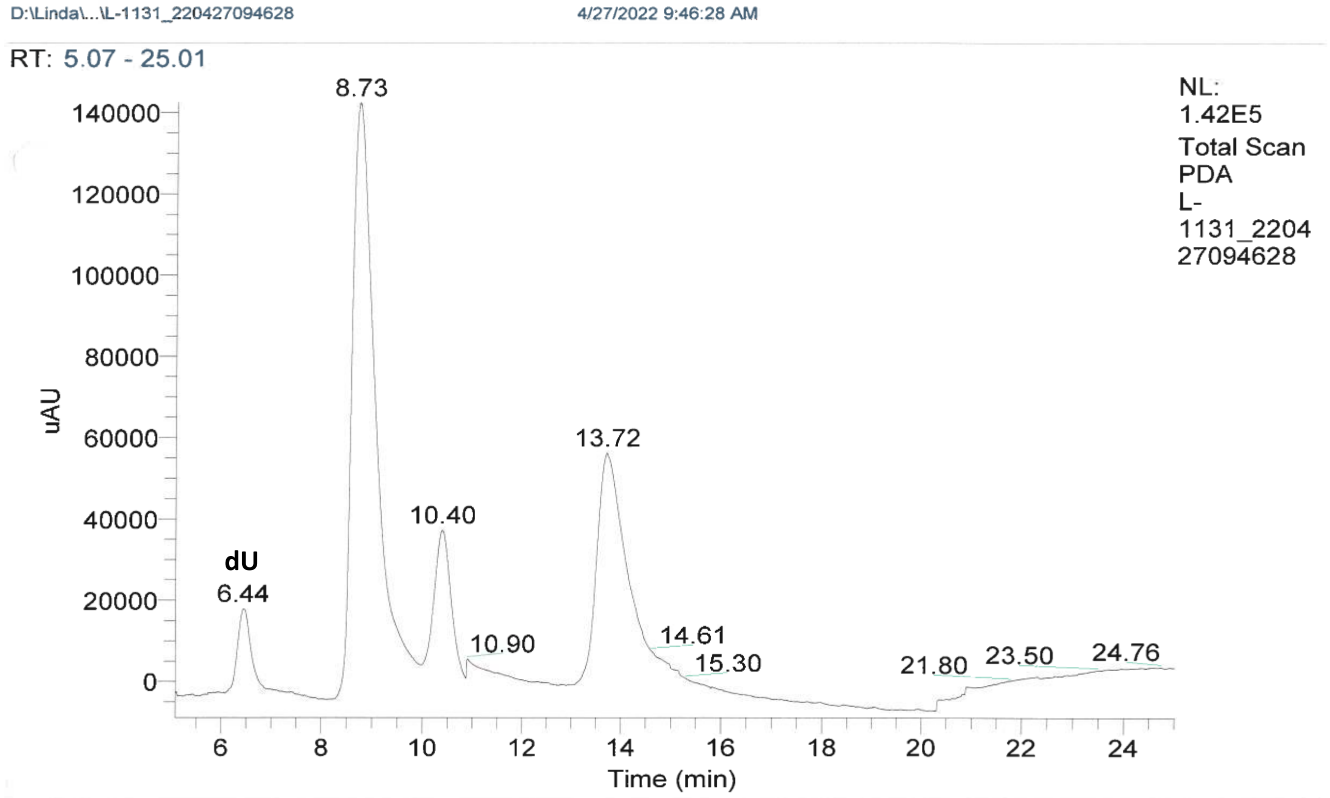

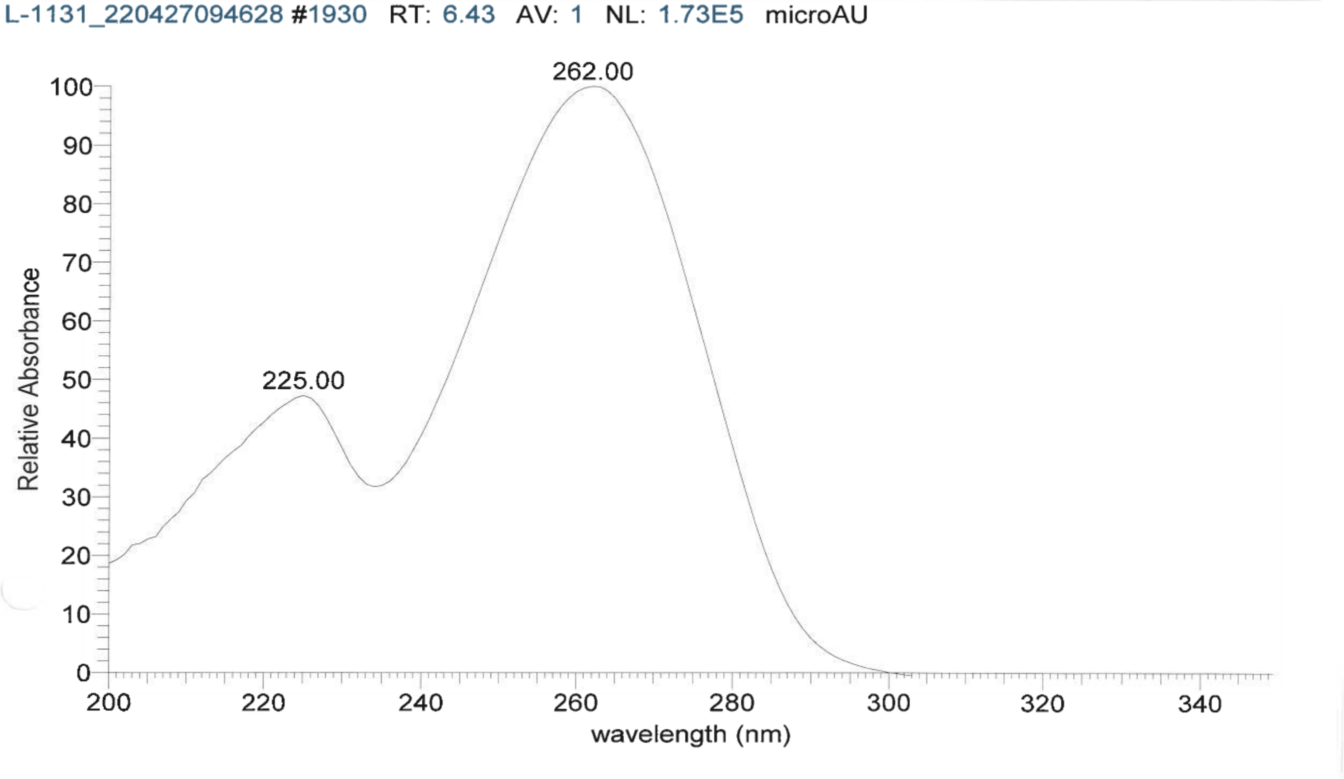

22mer-nonquad_5hmU

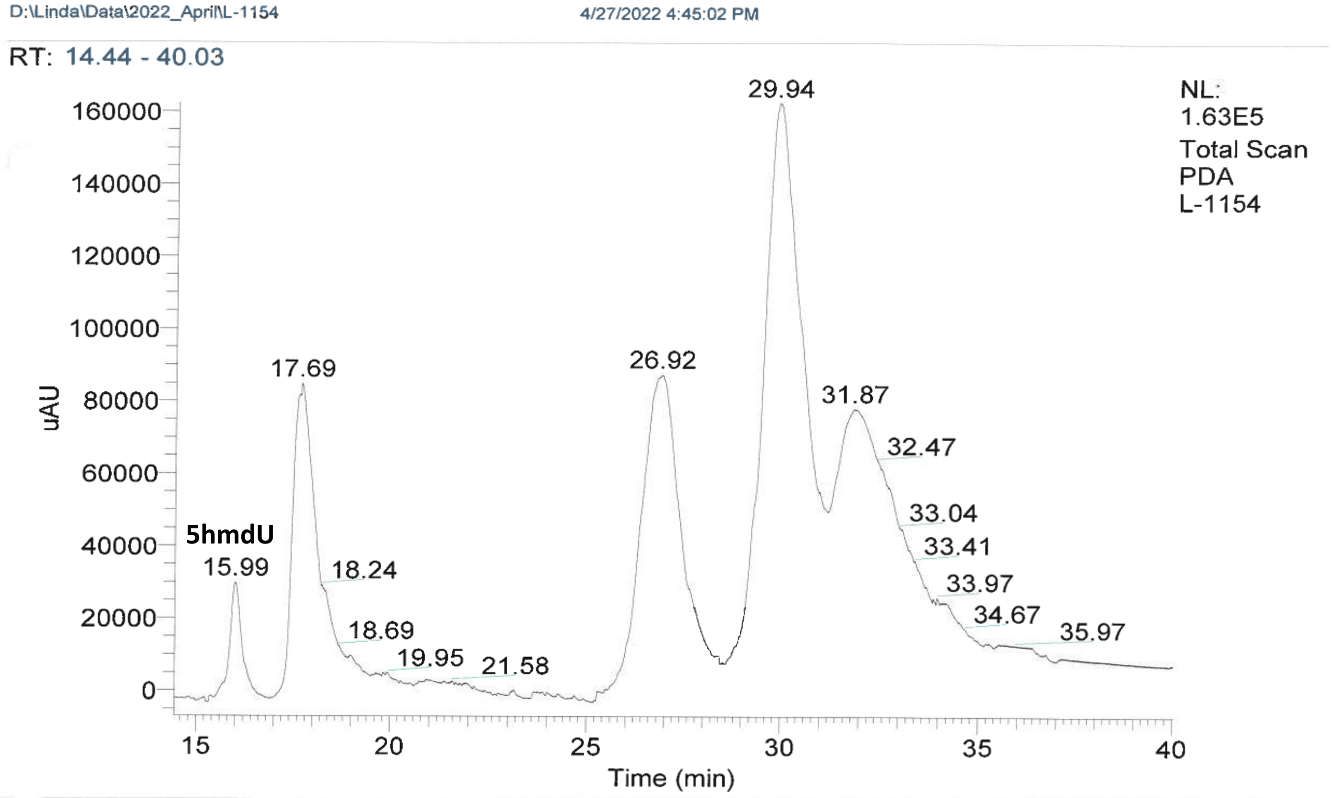

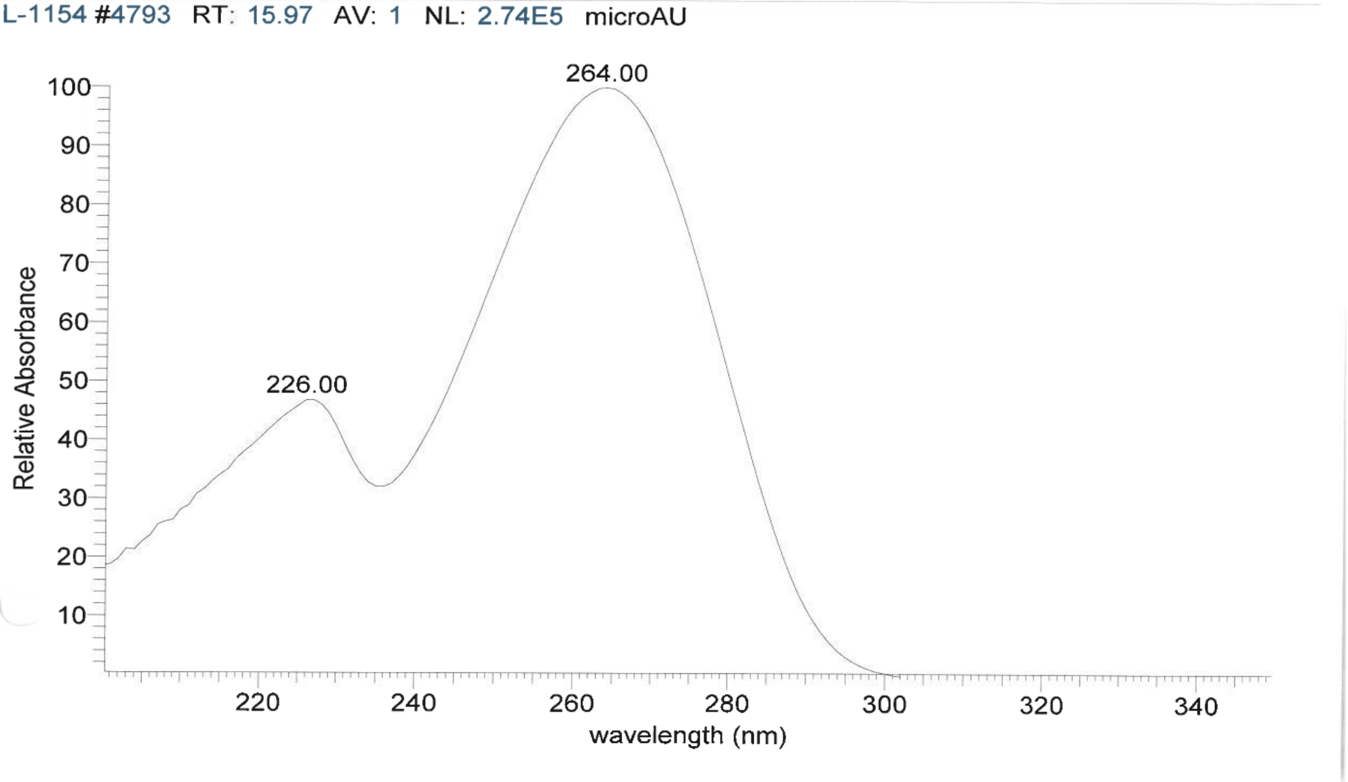

22mer-nonquad_U

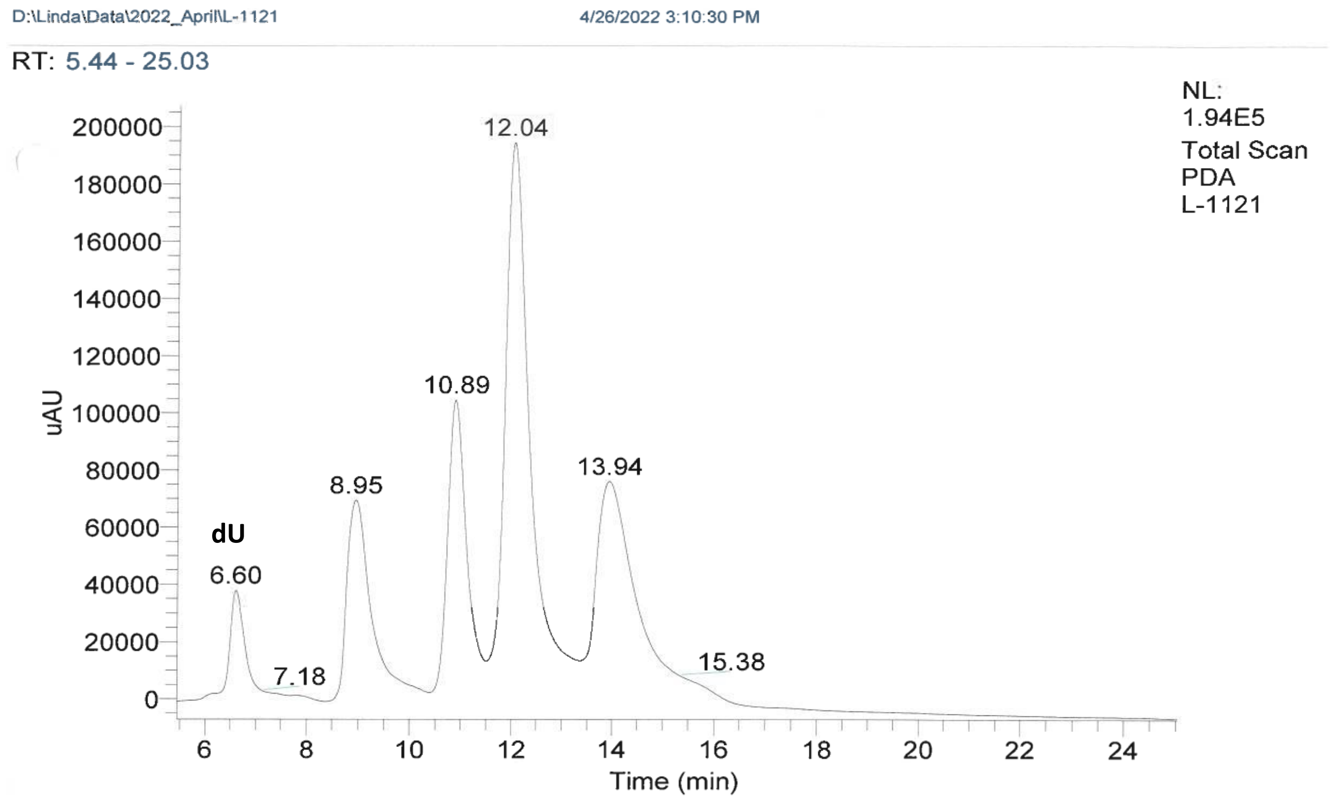

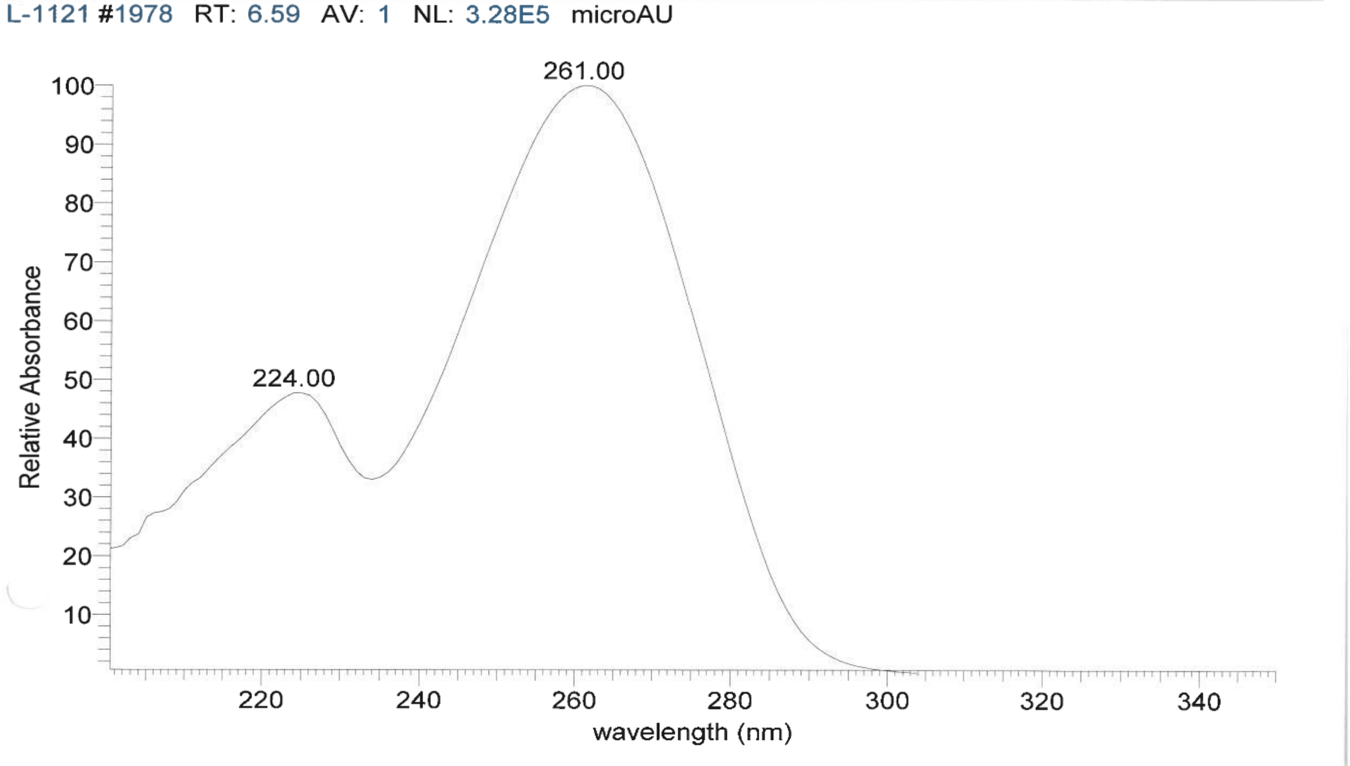

H. GC/MS results for oligonucleotides and methods

Table 5: Acid Hydrolysis and GC-MS Analysis of Oligonucleotides

Oligo	Modified Base	Retention Time (min.)	Silylated Formula	Silylated Mass (amu)	M-57 Ion (amu)	Data File (D:\Maggie\Data\Linda)
Quad-22mer-U	U	6.85	$C_{16}H_{32}N_2O_2Si_2$	340	283	\03092022\L-9123_scan_01.D
Quad-22mer-5FU	5FU	6.79	$C_{16}H_{31}FN_2O_2Si_2$	358	301	\03092022\L-9143_scan_01.D
Quad-22mer-5hmU[1]	5hmU	8.98	$C_{23}H_{48}N_2O_3Si_3$	484	427	\03092022\L-1051_scan_01.D
Quad-22mer-U-FAM	U	6.85	$C_{16}H_{32}N_2O_2Si_2$	340	283	\03092022\L-9113_scan_01.D
Quad-22mer-5FU-FAM	5FU	6.79	$C_{16}H_{31}FN_2O_2Si_2$	358	301	\03092022\L-1011_scan_01.D
Quad-22mer-5hmU-FAM[1]	5hmU	8.98	$C_{23}H_{48}N_2O_3Si_3$	484	427	\03092022\L-1041_scan_01.D
Quad-22mer-U-FAM-BHQ1	U	6.85	$C_{16}H_{32}N_2O_2Si_2$	340	283	\03092022\L-9118_scan_01.D
Quad-22mer-5FU-FAM-BHQ1	5FU	6.79	$C_{16}H_{31}FN_2O_2Si_2$	358	301	\03222022\L-1016_scan_01.D
Quad-22mer-5hmU-FAM-BHQ1[1]	5hmU	8.98	$C_{23}H_{48}N_2O_3Si_3$	484	427	\03092022\L-1046_scan_01.D
Quad-22mer-U-Comp	U	6.85	$C_{16}H_{32}N_2O_2Si_2$	340	283	\03092022\L-9128_scan_01.D
Quad-22mer-U-Comp-Cy5	U	6.85	$C_{16}H_{32}N_2O_2Si_2$	340	283	\04222022\L-1131_scan_01.D
22mer-nonquad_5hmU[1]	5hmU	8.98	$C_{23}H_{48}N_2O_3Si_3$	484	427	\04222022\L-1154_scan_01.D
22mer-nonquad_U	U	6.85	$C_{16}H_{32}N_2O_2Si_2$	340	283	\04222022\L-1121_scan_01.D

[1]Water step needed to remove formate ester formed in hydrolysis.

GC/MS: Formic acid hydrolysis was done on oligonucleotide (0.1 OD) with 88% formic acid (100 µL) at 140°C for 40 minutes in sealed GC vial. After cooling, the acid was removed under reduced pressure and the resulting bases were dissolved in anhydrous acetonitrile (20 µL) and N-(t-butyldimethylsilyl)-N-methyltrifluoroacetamide (MTBSTFA) with 1% tert-butyldimethylchlorosilane (TBDMCS) (20 µL) (ThermoFisher Scientific TS48927). The sealed vial was heated at 140°C for 40 minutes. The samples were cooled before being injected on an Agilent 7890A GC with Agilent J&W DB-5MS + DG column (30 m x 0.25 mm id, film thickness 0.25 µm) using helium carrier gas at 1 mL/min constant flow. The GC oven temperature was held at 100 °C for 2 minutes, ramped to 260 °C at 30 °C /minute then held for 10 minutes. The GC was directly coupled to an Agilent 5975C Mass Selective Detector with Triple-Axis detector. Each sample (0.5µL) was injected and run in SCAN mode.

Water step: Water (100 µL) was added after removing the formic acid and the sealed vial was heated at 140°C for 40 minutes then dried under reduced pressure. Derivatization was done as described above.

I. GC/MS spectra of hydrolyzed and derivatized oligonucleotides

Quad-22mer-U

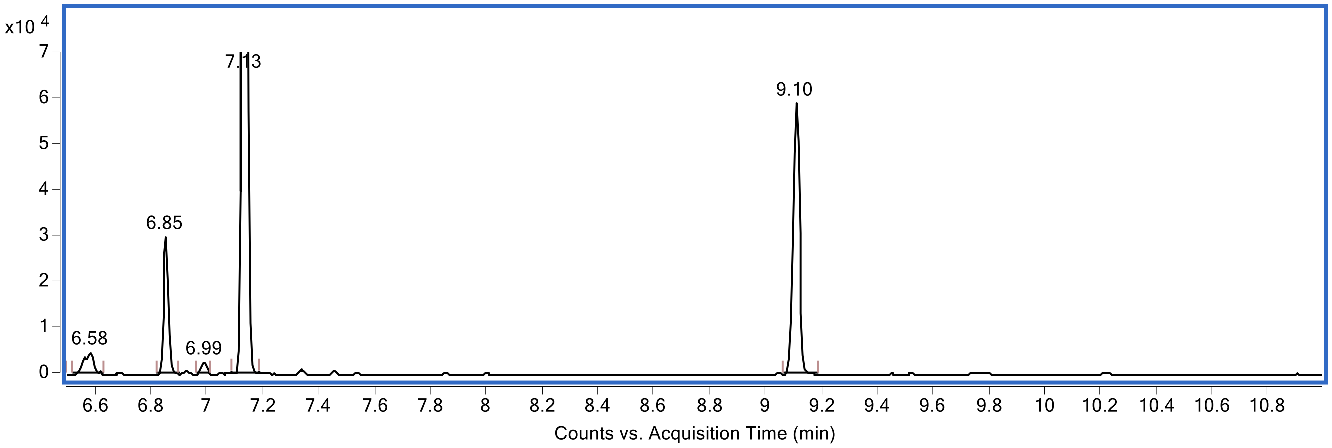

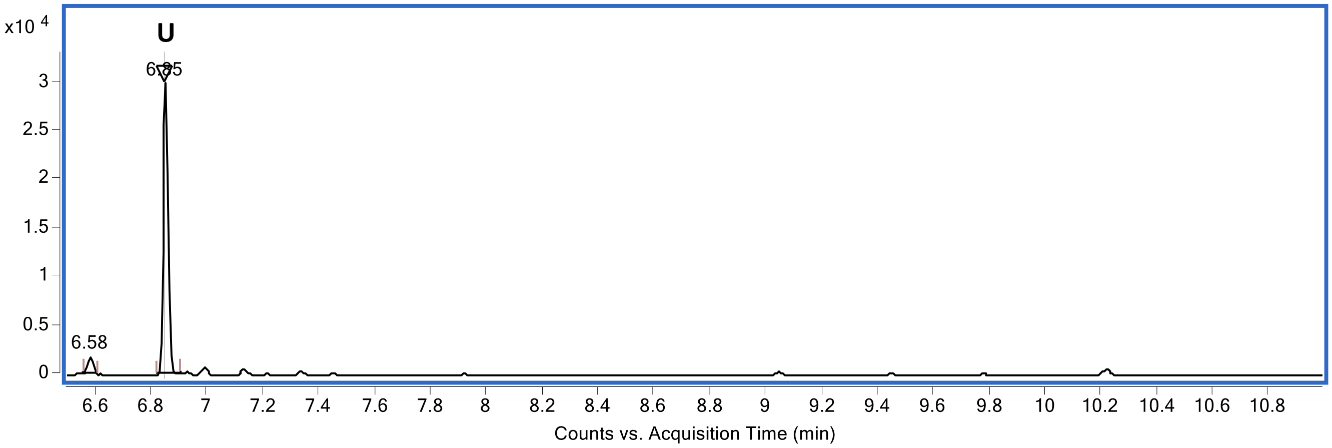

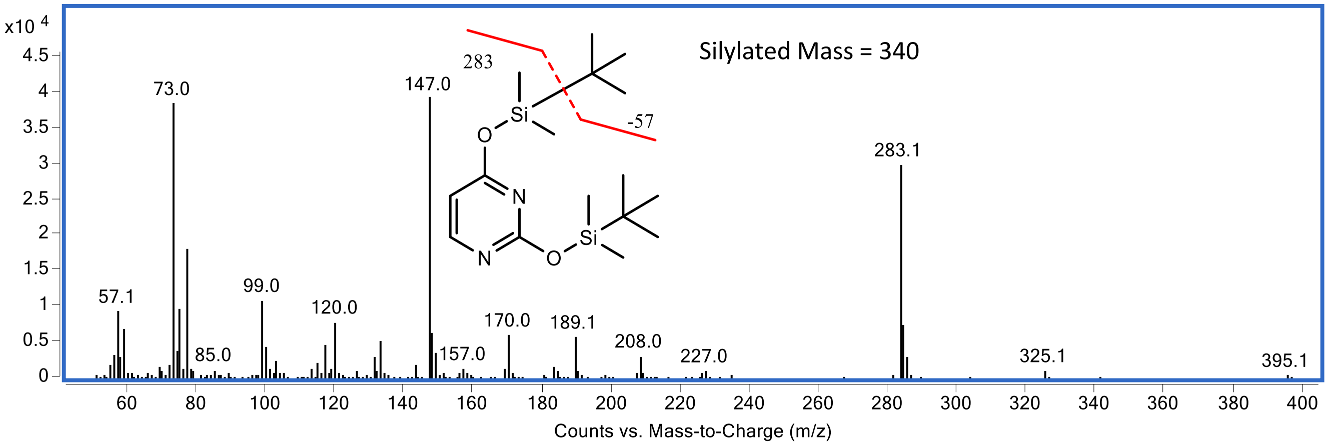

Quad-22mer-5FU

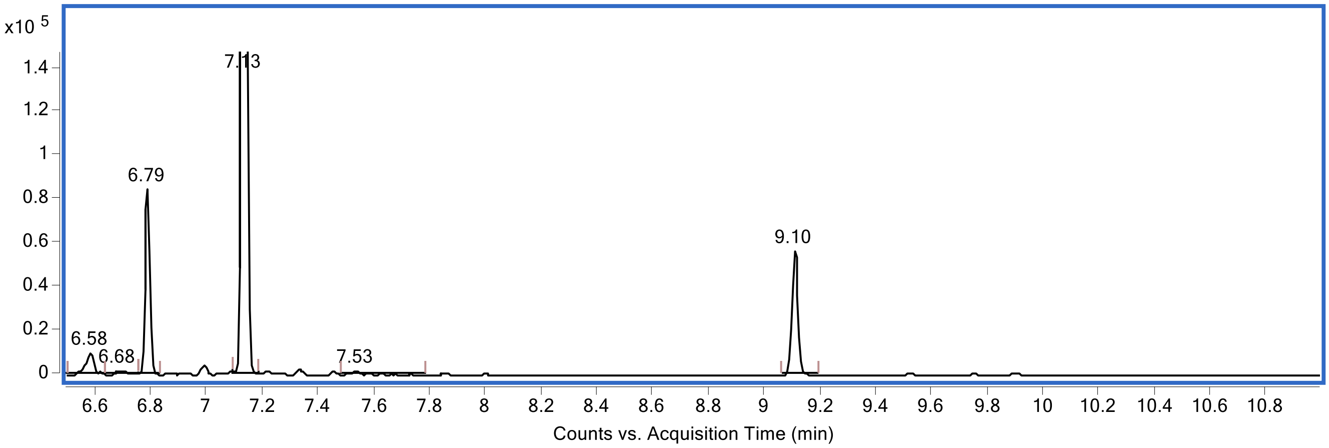

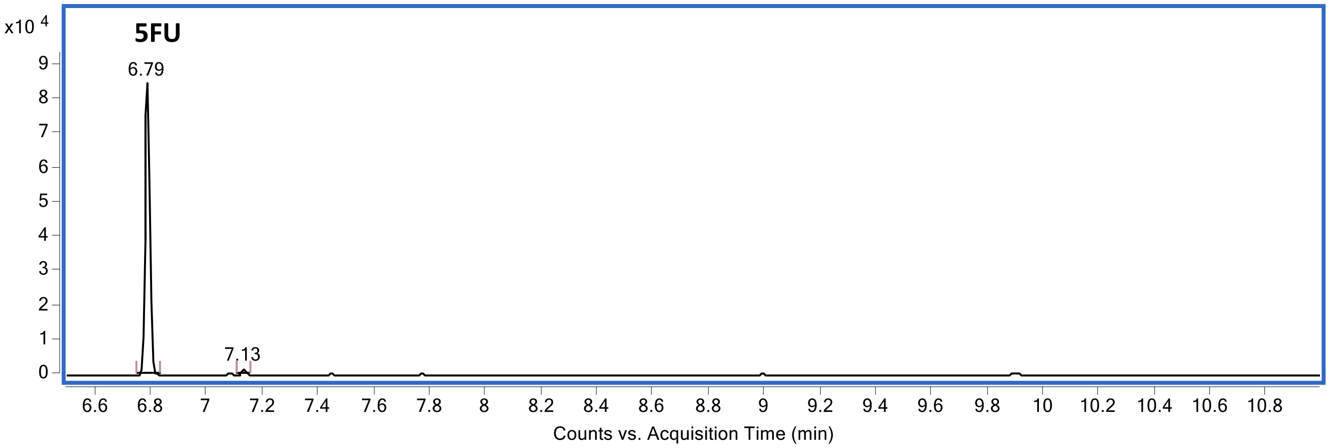

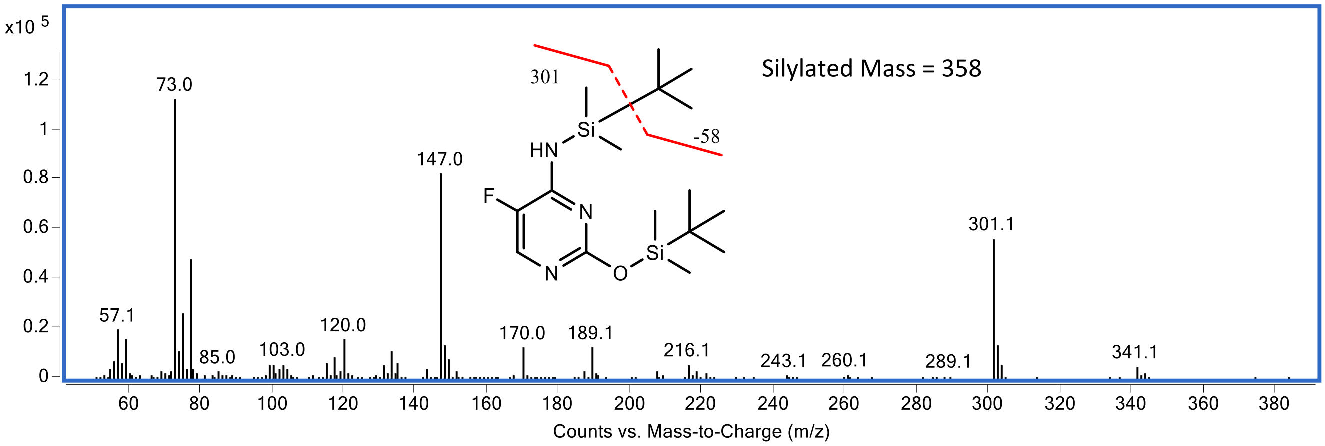

Quad-22mer-5hmU

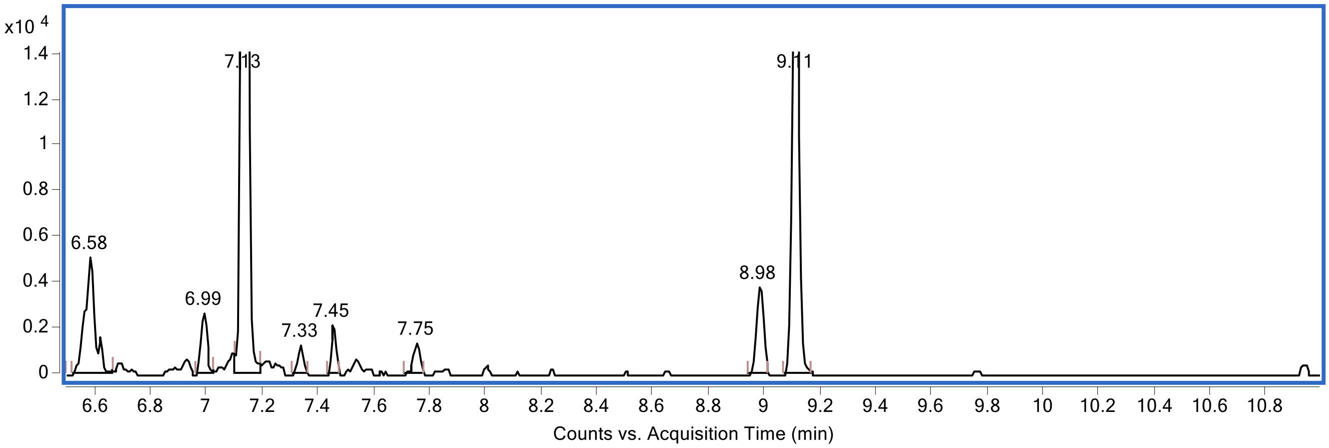

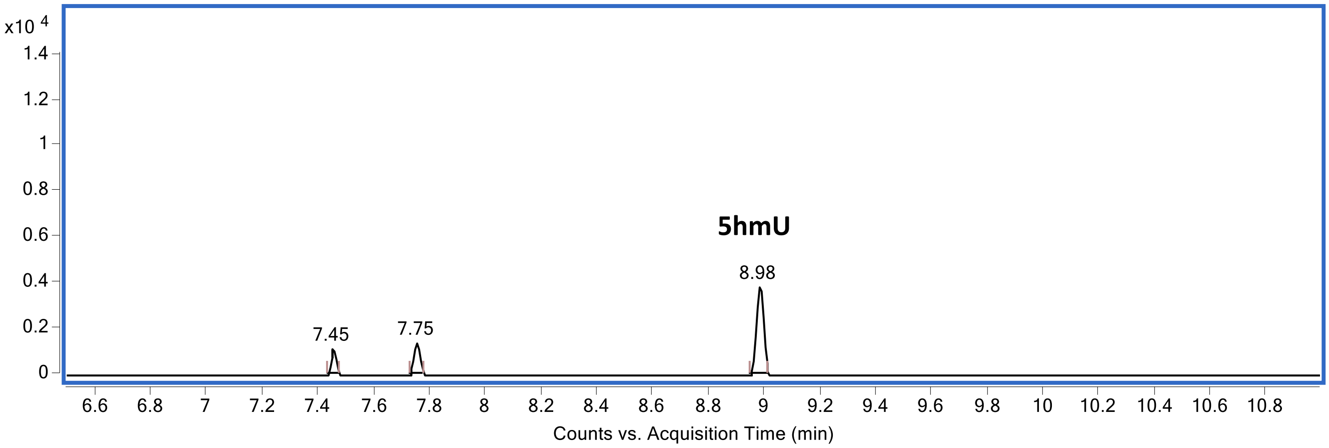

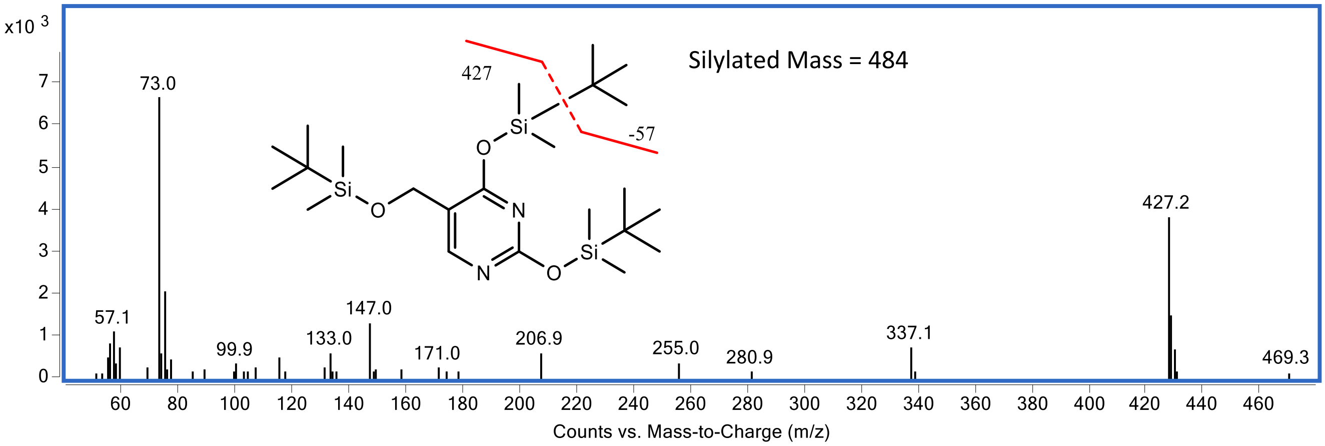

Quad-22mer-U-FAM

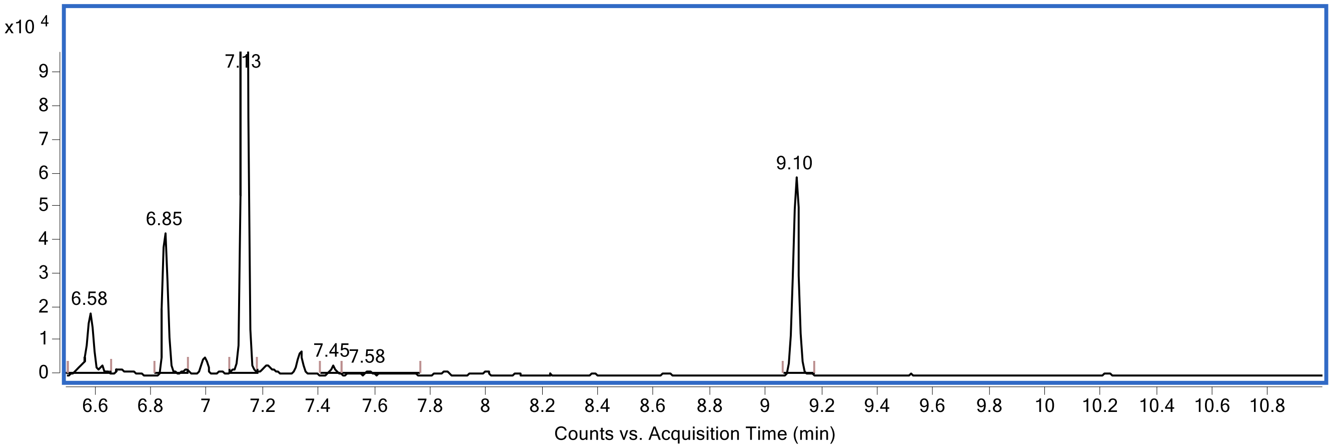

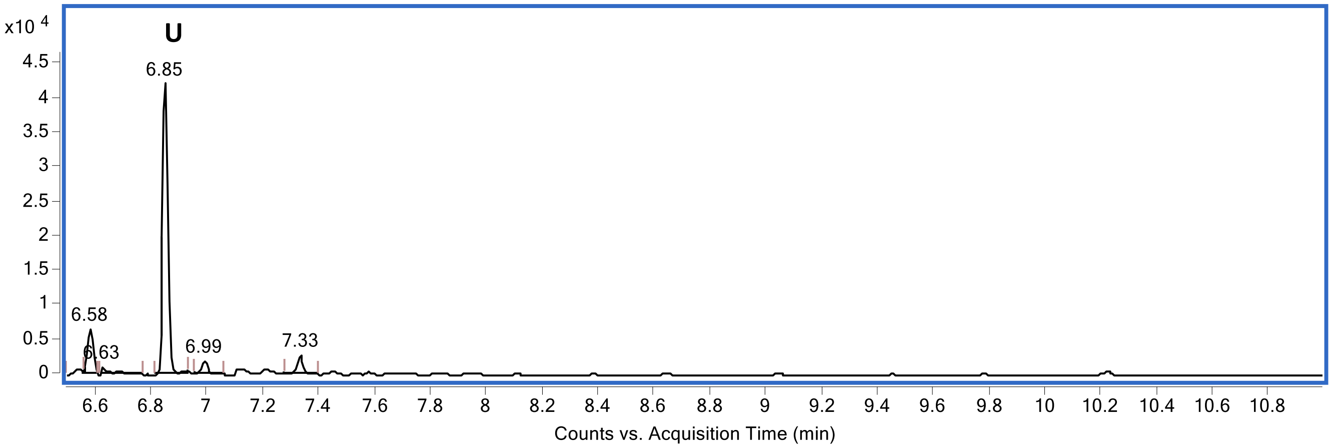

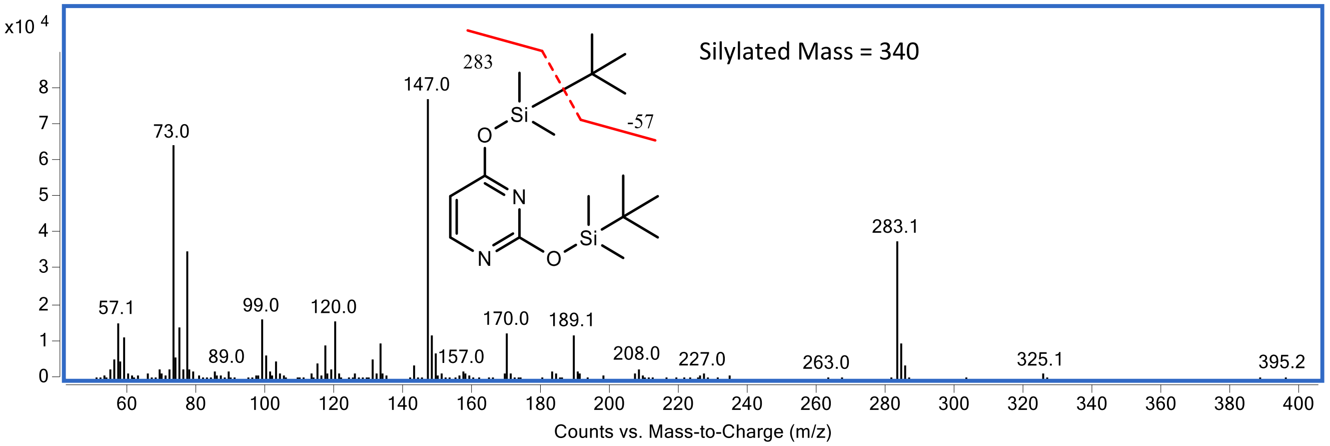

Quad-22mer-5FU-FAM

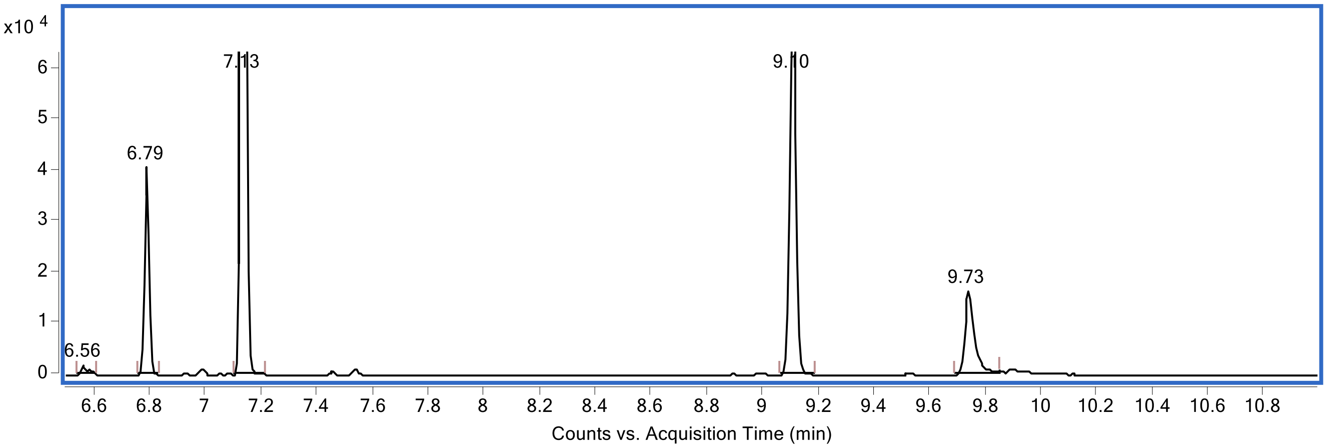

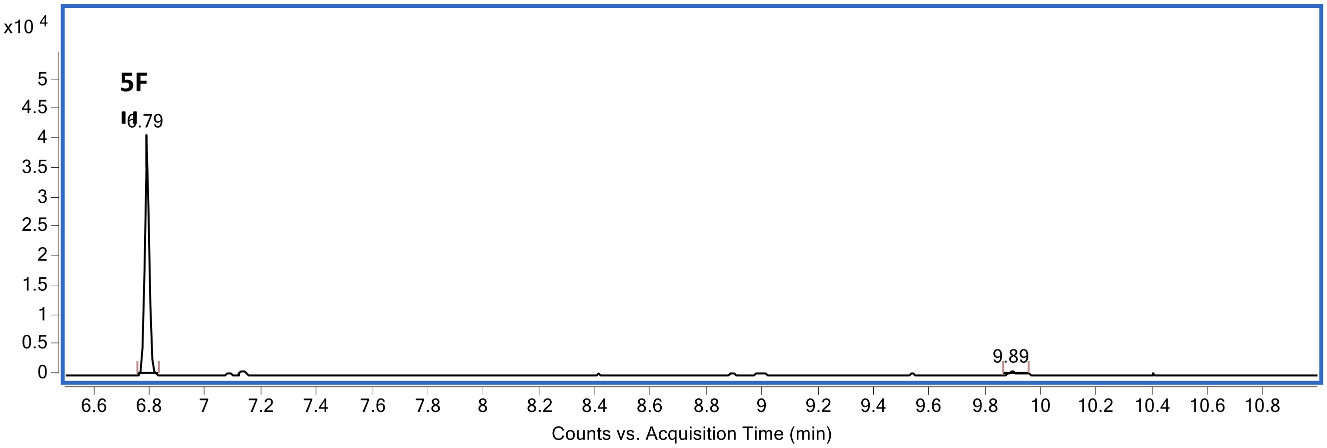

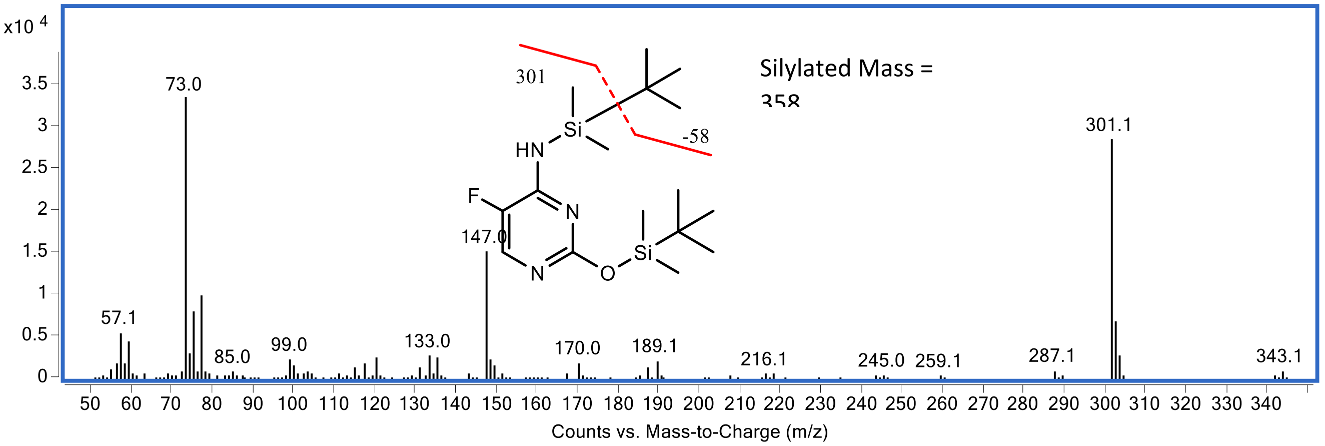

Quad-22mer-5hmU-FAM

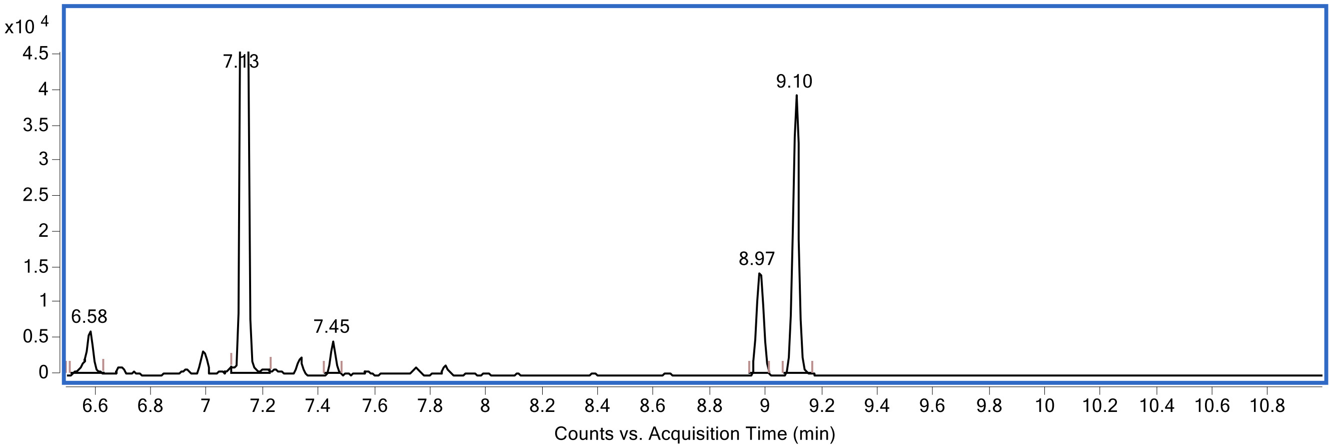

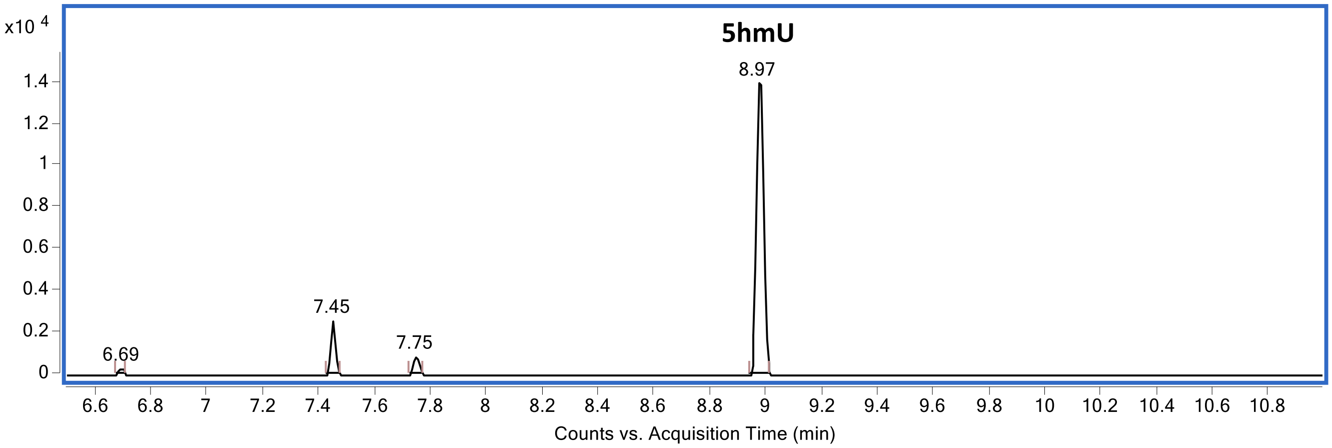

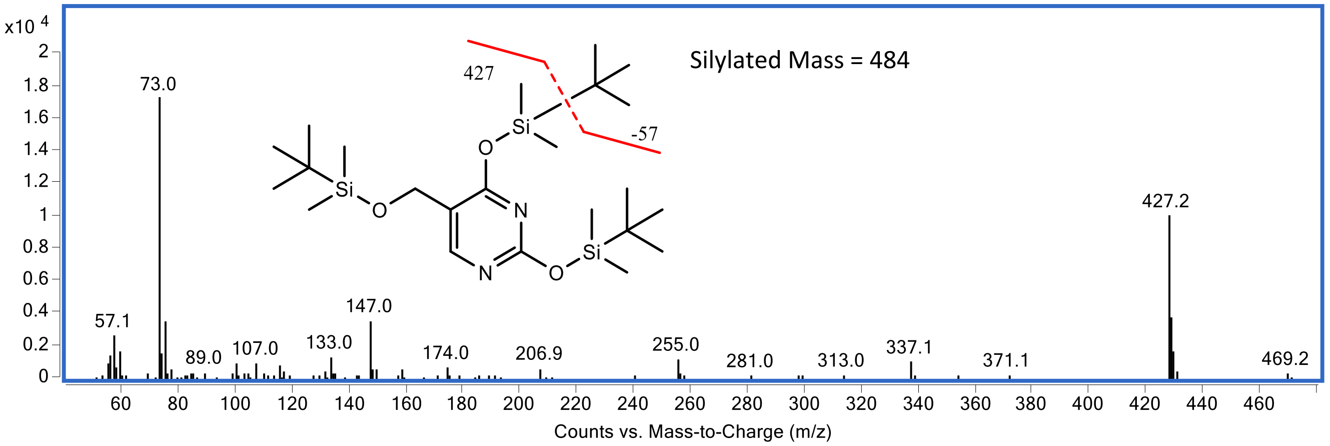

Quad-22mer-U-FAM-BHQ1

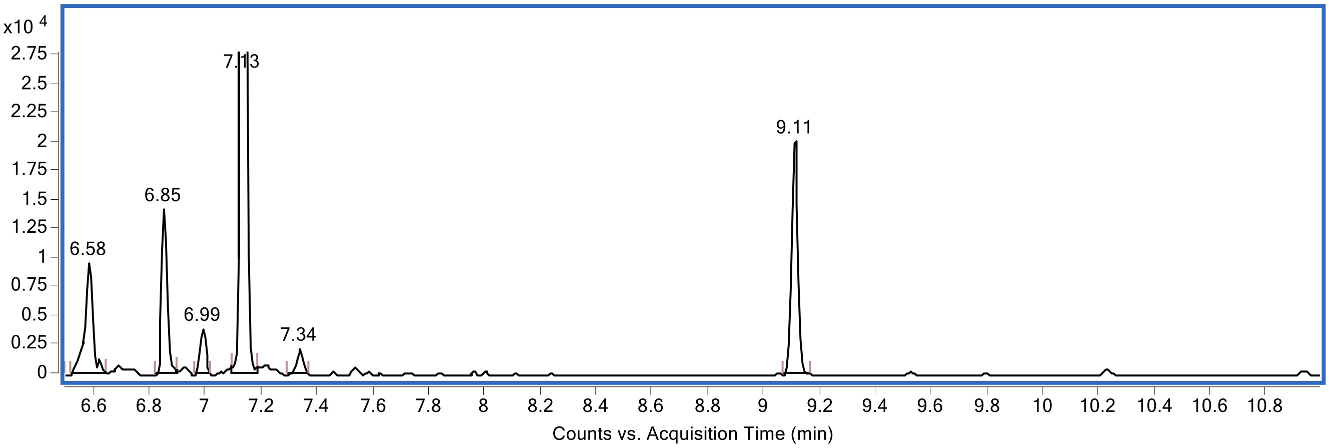

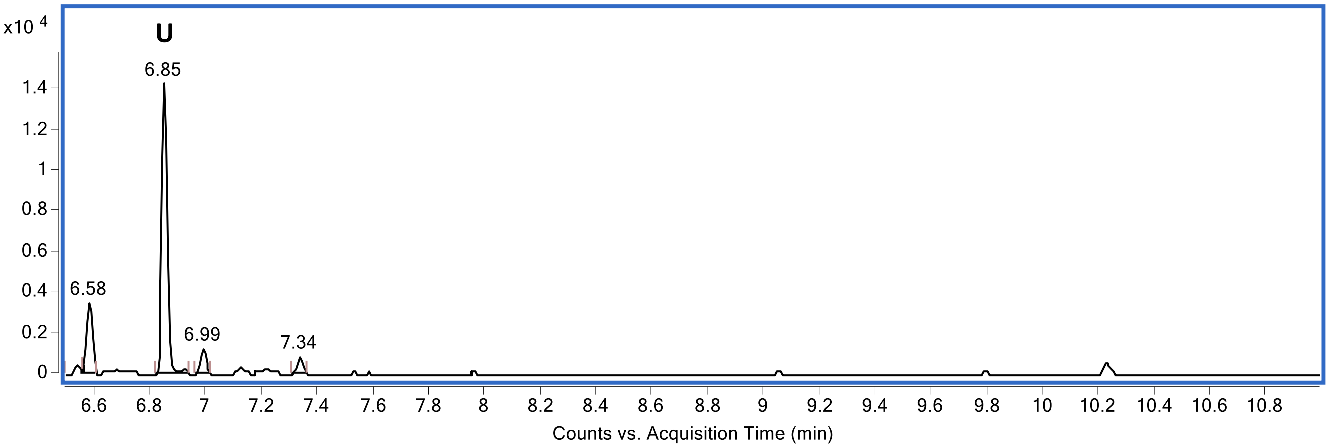

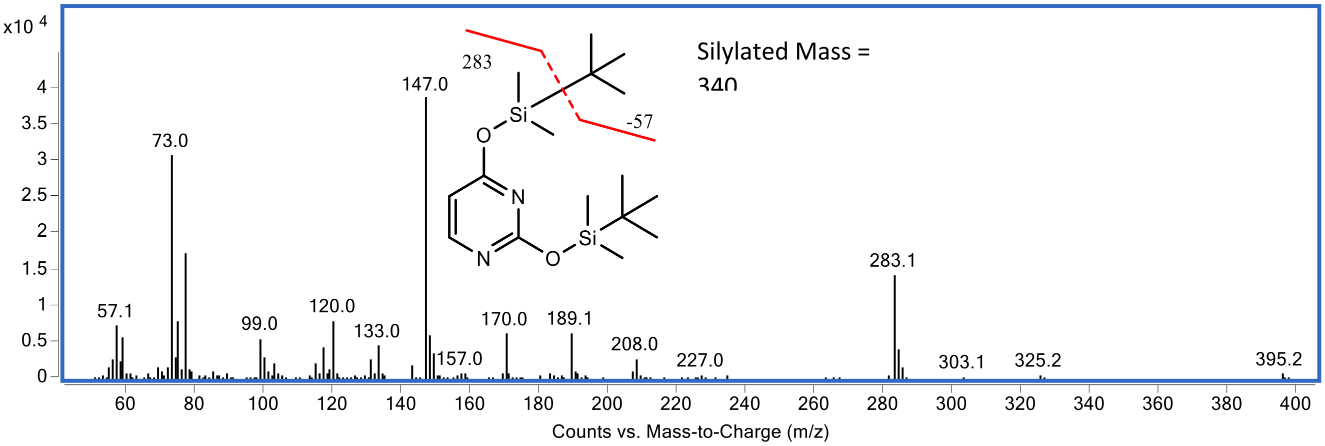

Quad-22mer-5FU-FAM-BHQ1

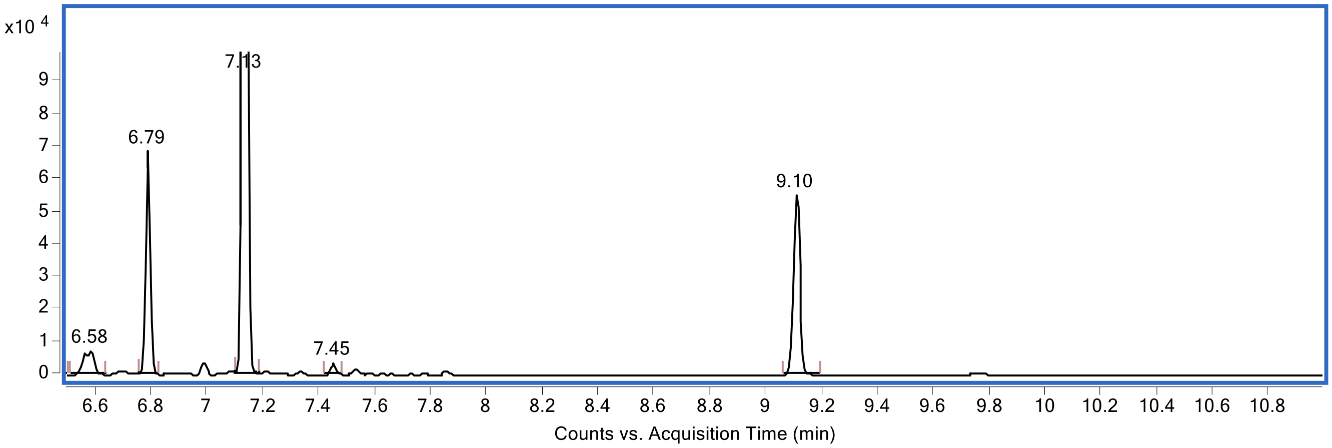

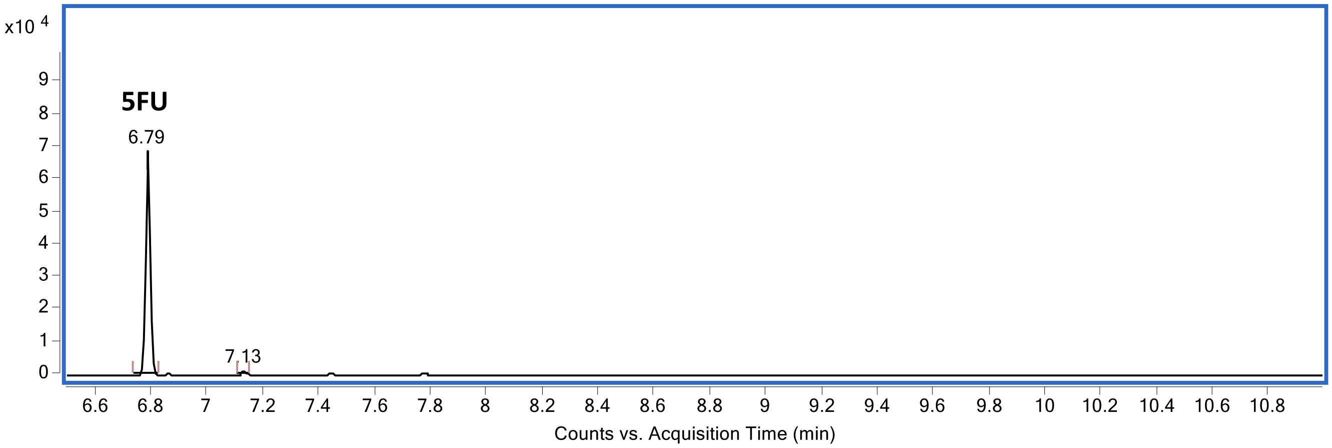

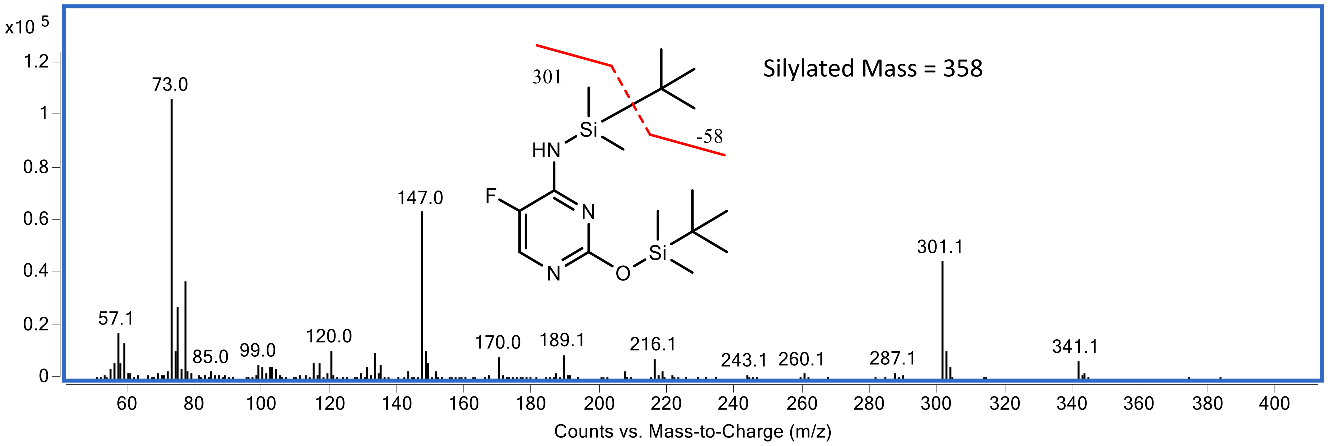

Quad-22mer-5hmU-FAM-BHQ1

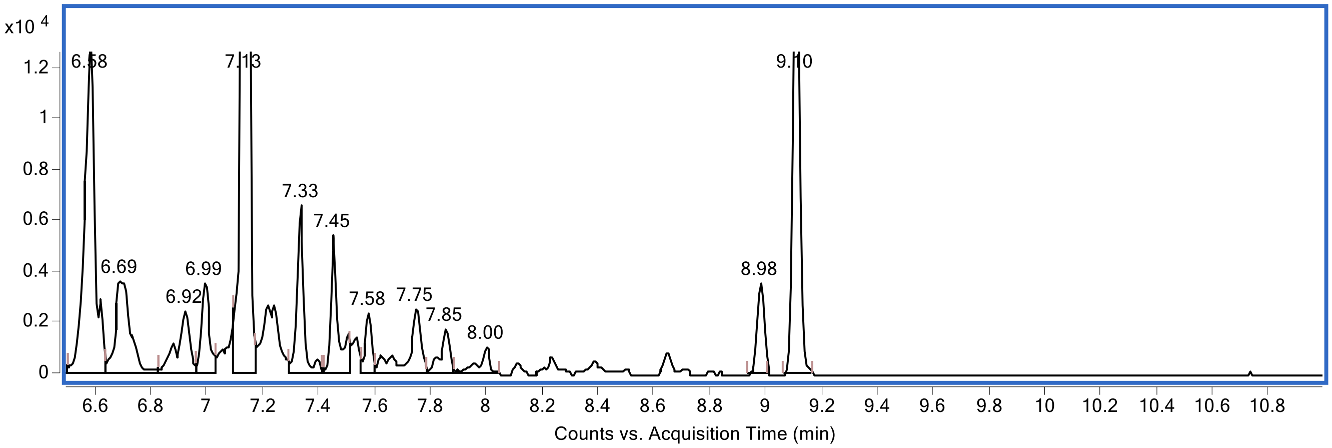

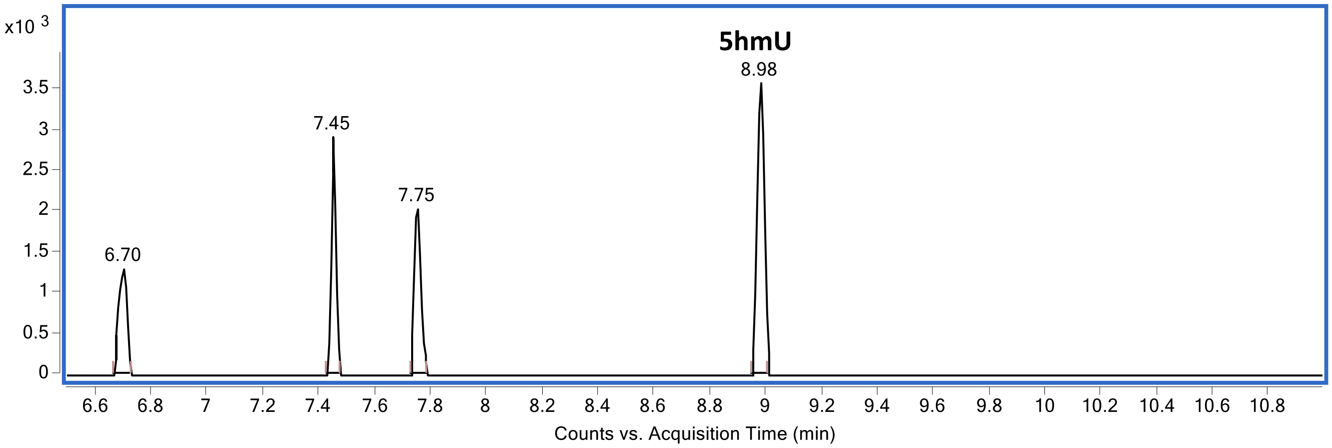

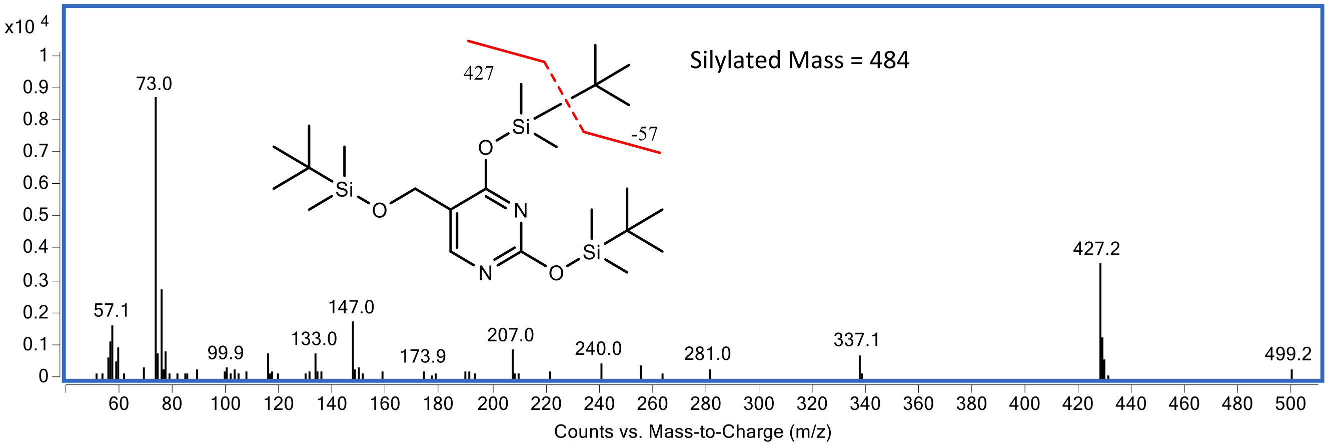

Quad-22mer-U-Comp

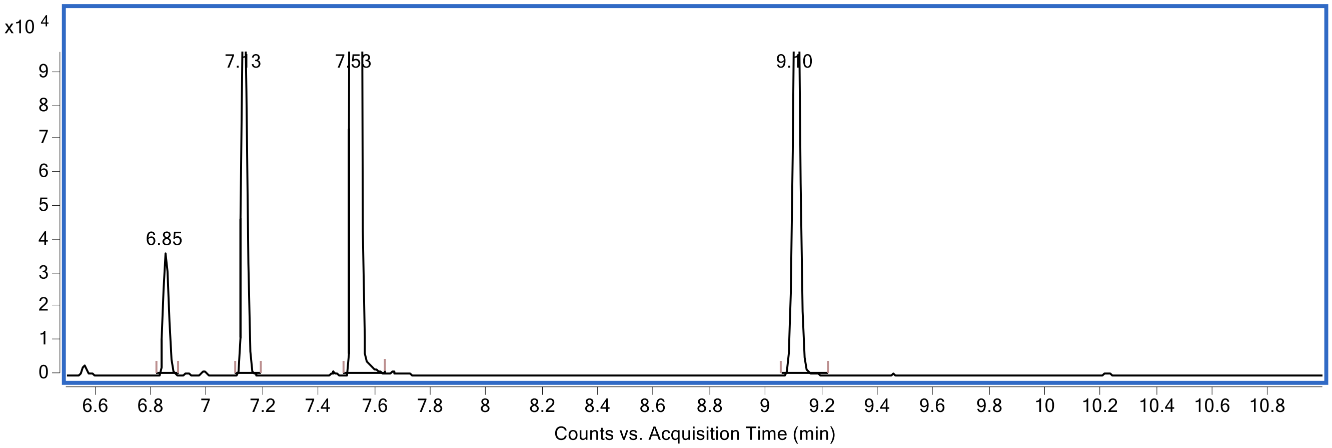

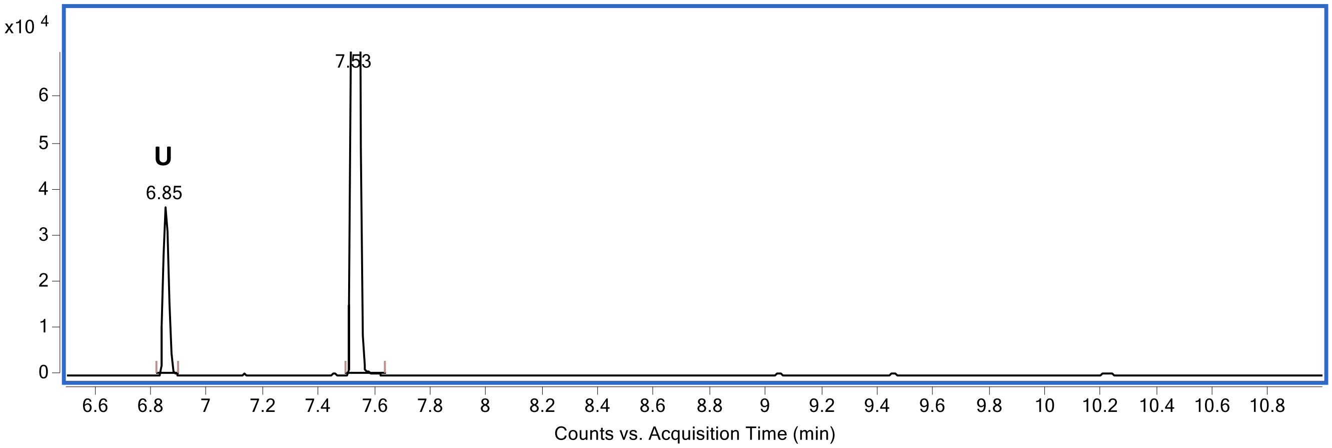

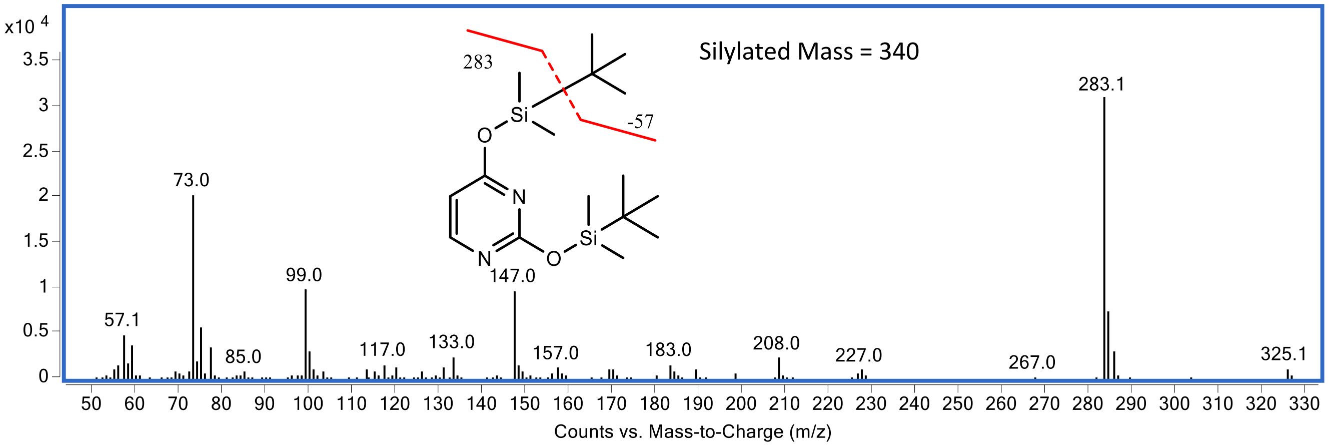

Quad-22mer-U-Comp-Cy5

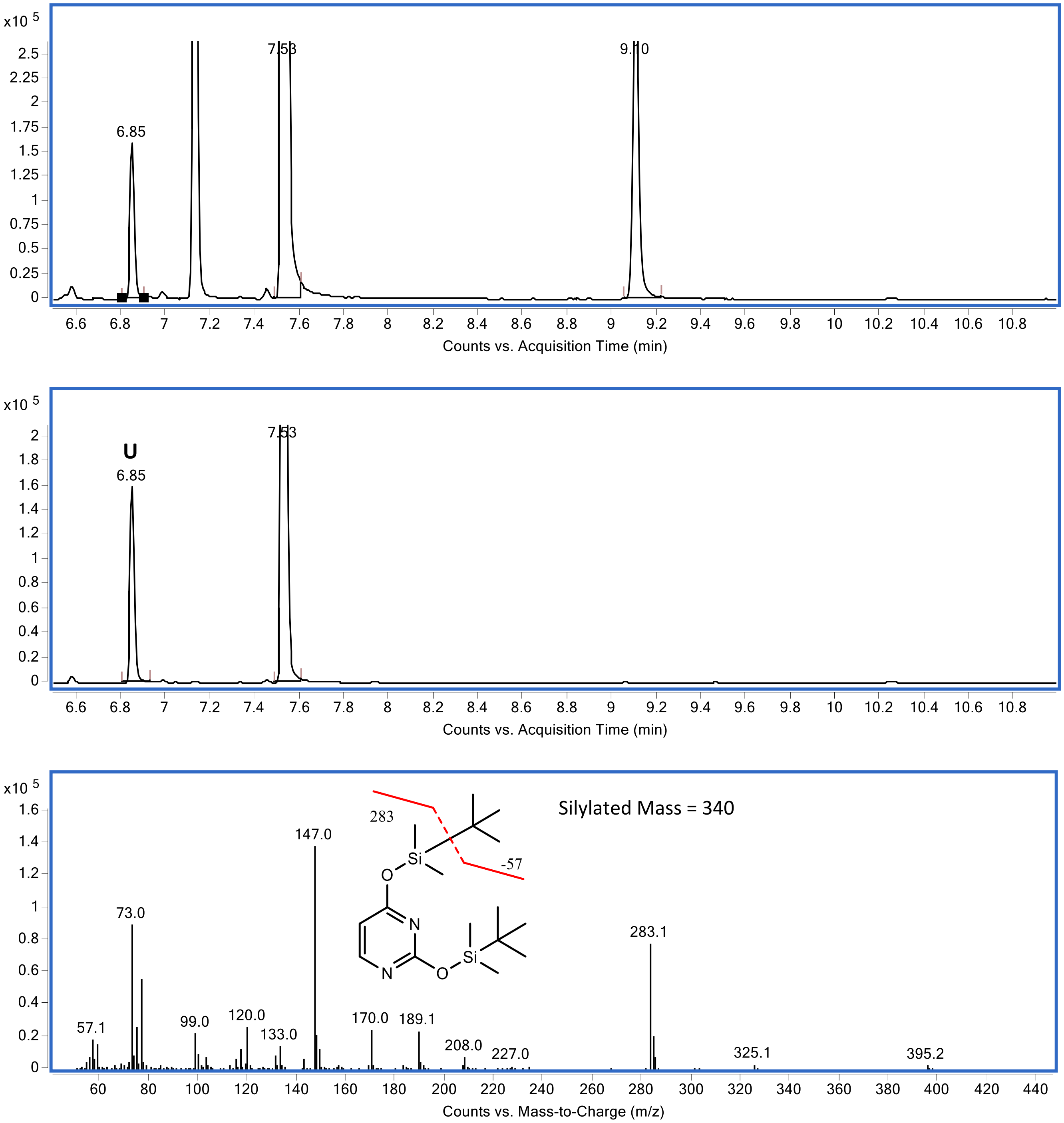

22mer-nonquad_5hmU

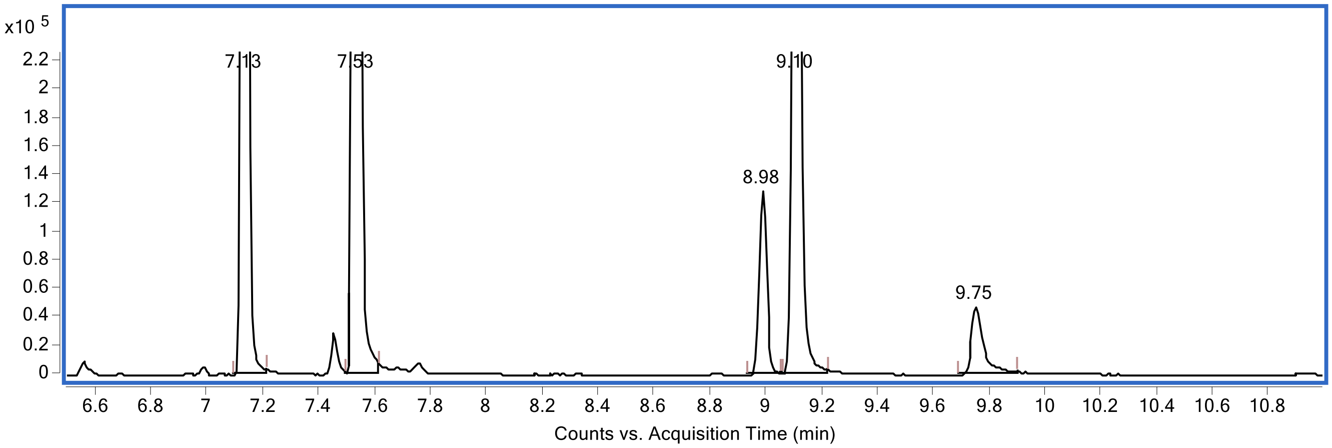

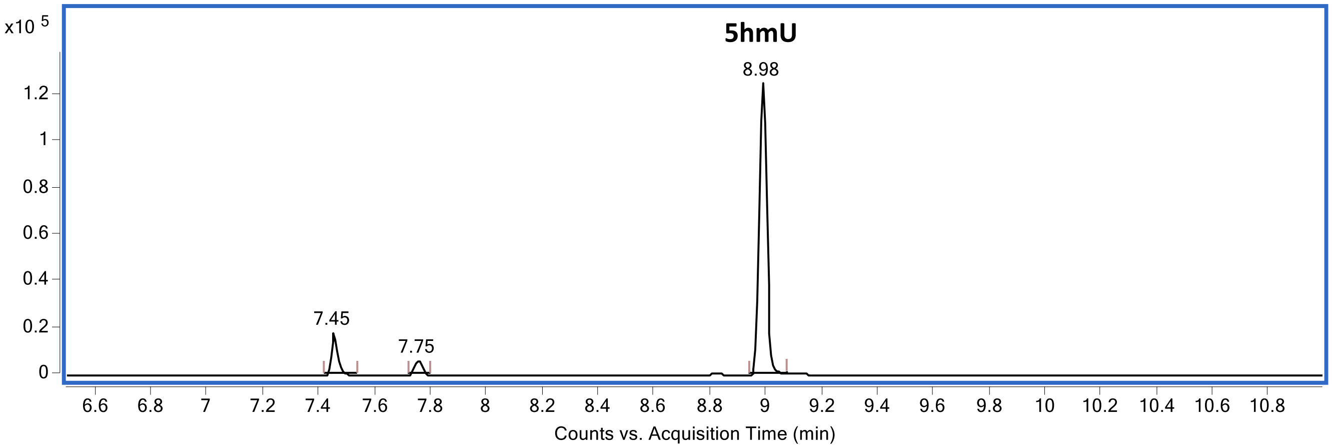

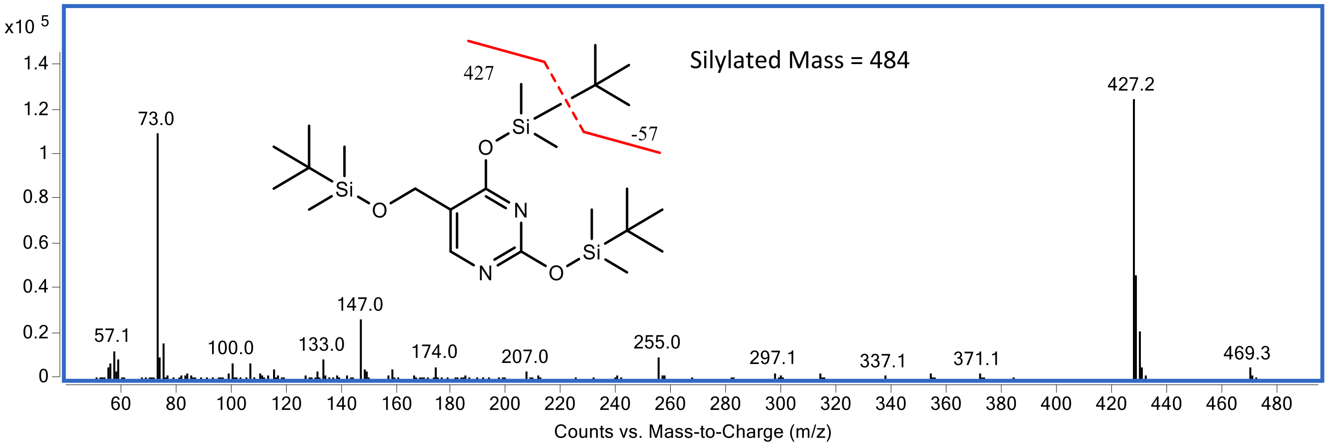

22mer-nonquad_U

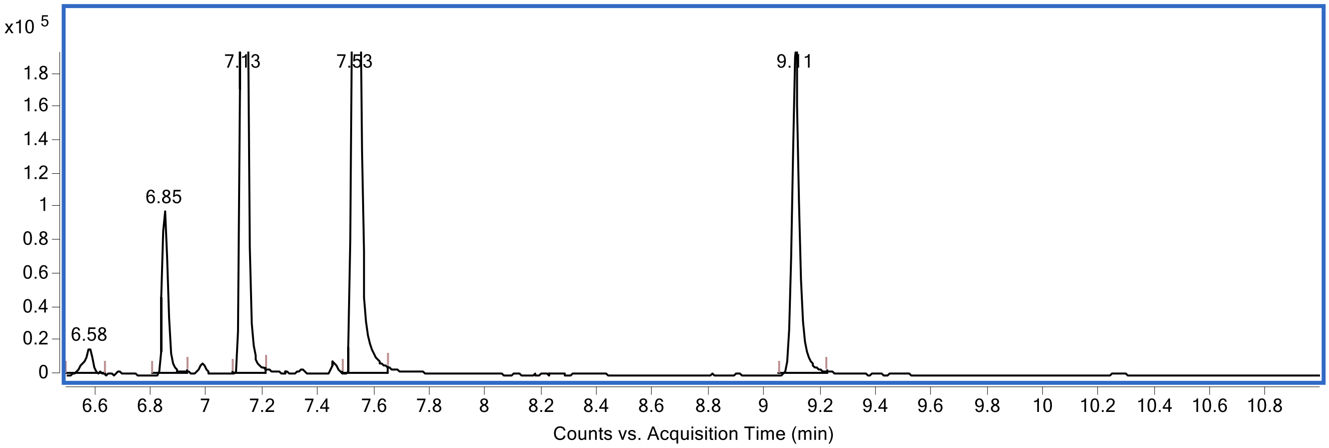

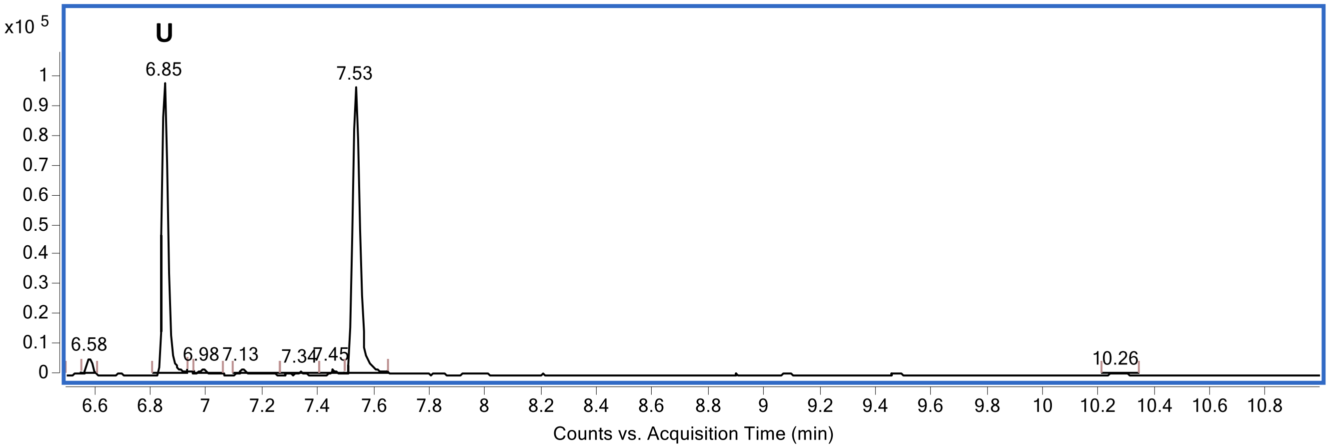

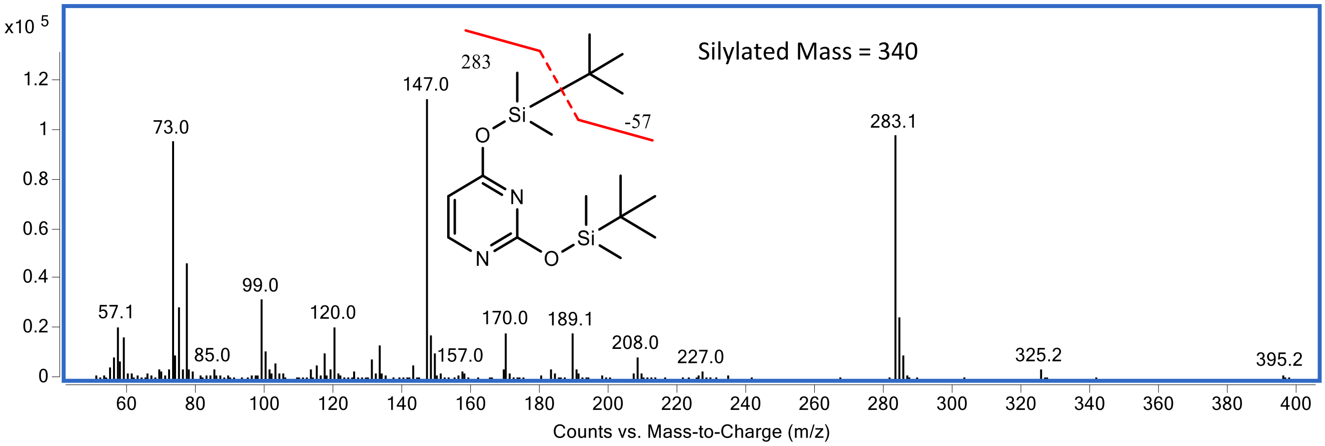

Supplement: Supplementary file 1 [file molecules-28-00970-s001.zip › Supplementary Appendix_2023-01-14.pdf]
