# Supplementary material for: DNA Base Excision Repair Intermediates Influence Duplex–Quadruplex Equilibrium
Source: Molecules. 2023 Jan 18;28(3):970. doi: 10.3390/molecules28030970 (PMC9920732; doi:10.3390/molecules28030970)
Supplement: Supplementary file 1 [file molecules-28-00970-s001.zip › Supporting Information_2023-01-15.pdf]

DNA Base Excision Repair Intermediates Influence Duplex—Quadruplex Equilibrium

Mark L. Sowers [1,2], James W. Conrad[1], Bruce Chang-Gu[1,2], Ellie Cherryhomes[1], Linda C. Hackfeld[1], and Lawrence C. Sowers[1,3,&]

[1]Department of Pharmacology and Toxicology, 301 University Boulevard, Galveston, TX 77555, USA
[2]MD-PhD Combined Degree Program, 301 University Boulevard, Galveston, TX 77555, USA
[3]Department of Internal Medicine, University of Texas Medical Branch, 301 University Boulevard, Galveston, TX 77555, USA

[&]Address correspondence to Lawrence Sowers, e-mail lasowers@UTMB.edu, phone 409-772-9678.

Supporting Information

Table S1. DNA glycosylase and APE1 rate constants for Figure 3.

Figure S1. Unedited gel scans of UDG time course with U:A substrate in Fig. 3A.
Figure S2. Unedited gel scans of UDG time course with Tel22-U substrate in Fig. 3A.
Figure S3. Unedited gel scans of UDG time course with NQ-U substrate in Fig. 3A.
Figure S4. Unedited gel scans of UDG time course with 5FU:A substrate in Fig. 3B.
Figure S5. Unedited gel scans of UDG time course with Tel22-5FU substrate in Fig. 3B.
Figure S6. Unedited gel scans of UDG time course with NQ-5FU substrate in Fig. 3B.
Figure S7. Unedited gel scans of hSMUG1 time course with substrate U:A in Fig. 3C.
Figure S8. Unedited gel scans of hSMUG1 time course with substrate Tel22-U in Fig. 3C.
Figure S9. Unedited gel scans of hSMUG1 time course with substrate NQ-U in Fig. 3C.
Figure S10. Unedited gel scans of hSMUG1 time course with substrate 5hmU:A in Fig. 3D.
Figure S11. Unedited gel scans of hSMUG1 time course with substrate Tel22-5hmU in Fig. 3D.
Figure S12. Unedited gel scans of hSMUG1 time course with substrate NQ-5hmU in Fig. 3D.
Figure S13. Unedited gel scans of APE 1 time course with substrate THF:A in Fig. 3E.
Figure S14. Unedited gel scans of APE 1 time course with substrate Tel22-THF in Fig. 3E.
Figure S15. Unedited gel scans of APE 1 time course with substrate NQ-THF in Fig. 3E.
Figure S16. Unedited gel scans of the β-elimination time course with UDG treated substrate Tel22-U in Fig. 3F.
Figure S17. Quadruplexes quench fluorescence compared to duplex.
Figure S18. Rate of gap formation in Figure 6 time course.

	**UDG**	**hSMUG1**	**APE 1**
Rate Constant	*k* (min^{-1})	*k* (min^{-1})	*k* (min^{-1})
U:A	0.2827	0.1288	
Gquad U	0.1518	0.08188	
Non-quad U	0.4972	0.8288	
5FU:A	0.1498		
Gquad 5FU	0.1050		
Non-quad 5FU	0.2935		
5hmU:A		0.07423	
Gquad 5hmU		0.02041	
Non-quad 5hmU		0.4537	
THF:A			0.6587
Gquad THF			NA
Non-quad THF			NA

Table S1. DNA glycosylase and APE1 rate constants for Figure 3. A single exponential fit was used with the y-intercept fixed to 0. Fitting was done in PRISM 9.4.1.

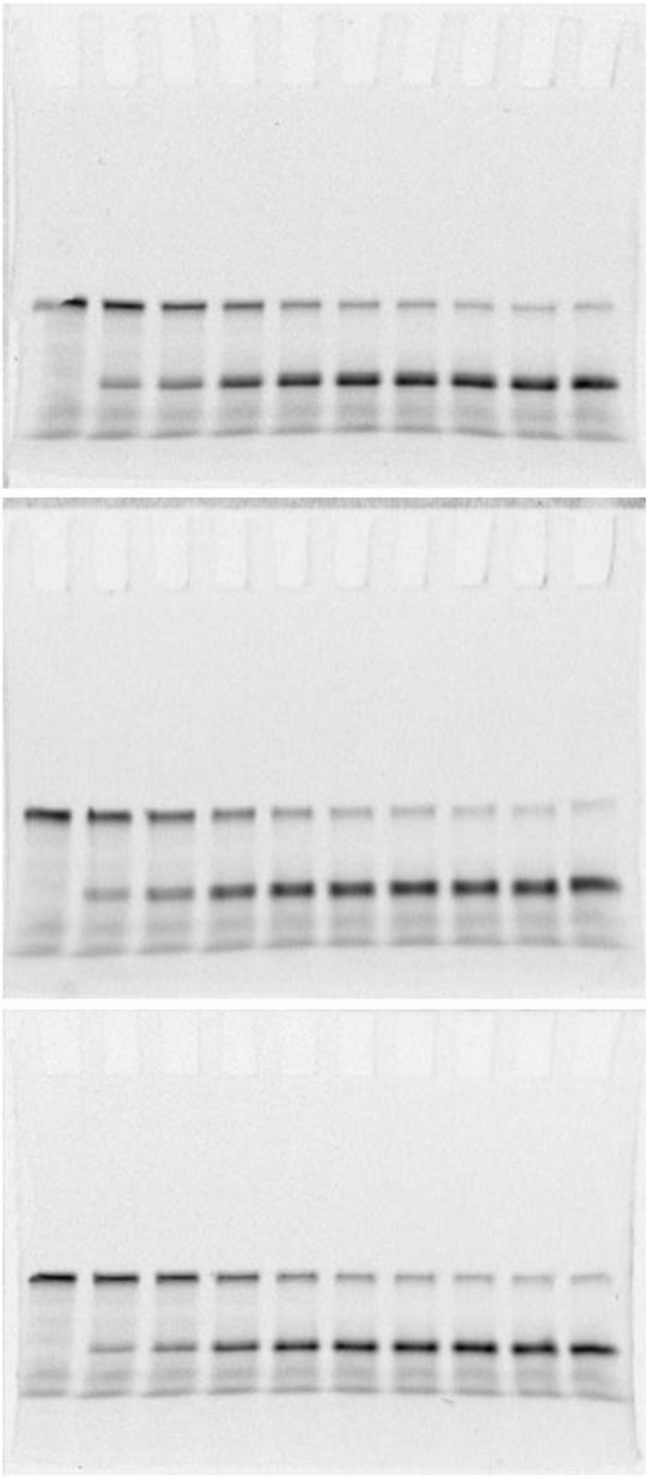

Figure S1. Unedited gel scans of UDG time course with U:A substrate in Fig. 3A.

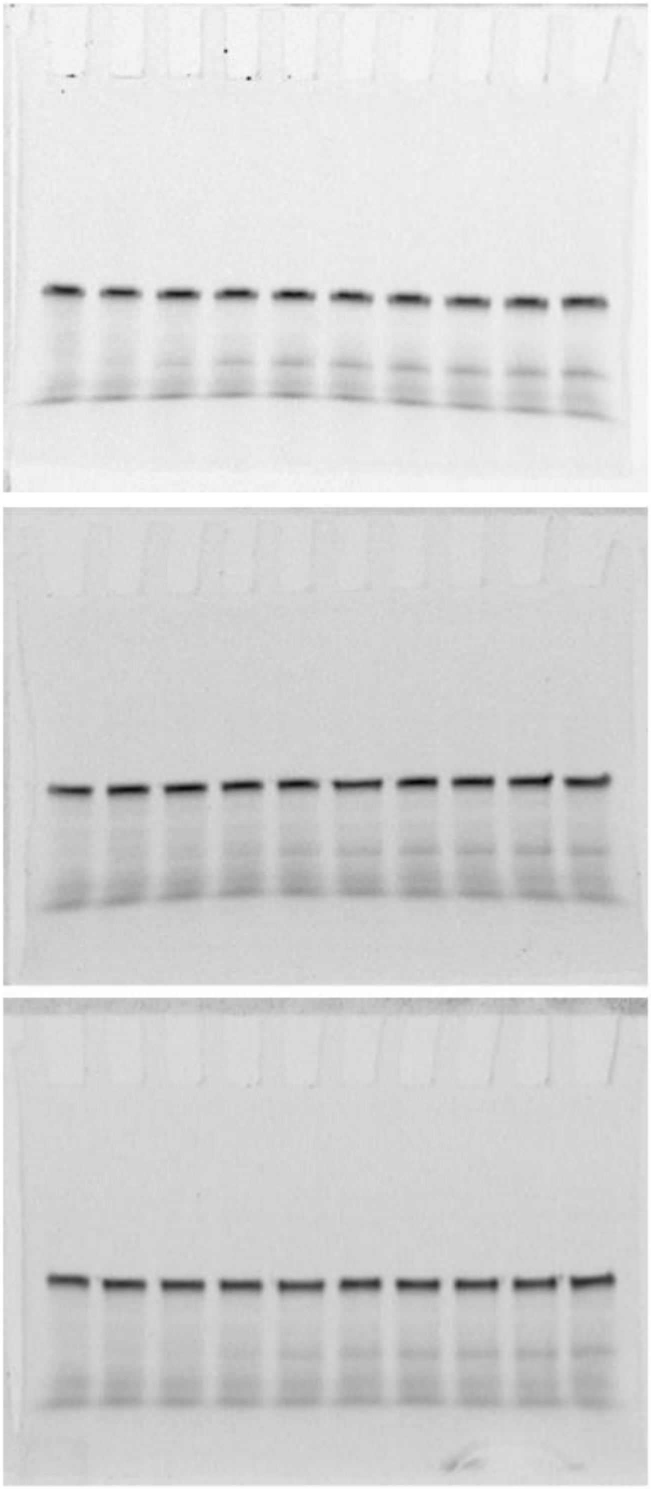

Figure S2. Unedited gel scans of UDG time course with Tel22-U substrate in Fig. 3A.

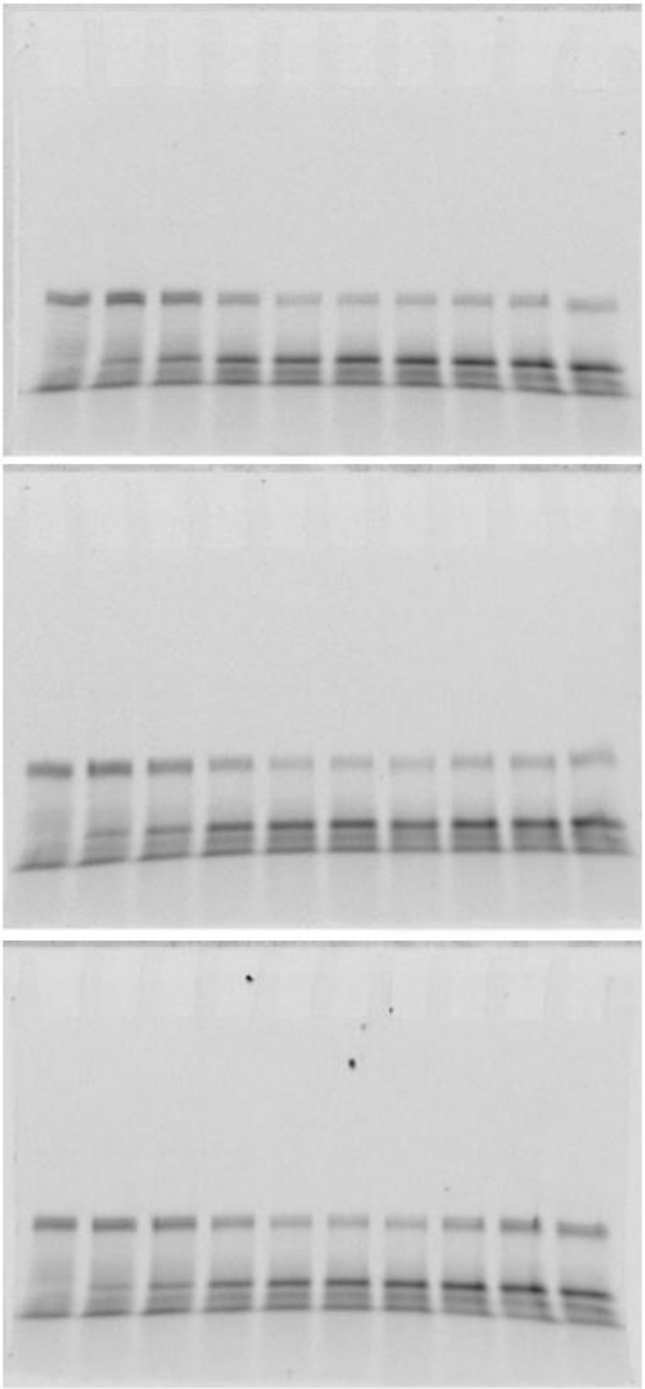

Figure S3. Unedited gel scans of UDG time course with NQ-U substrate in Fig. 3A.

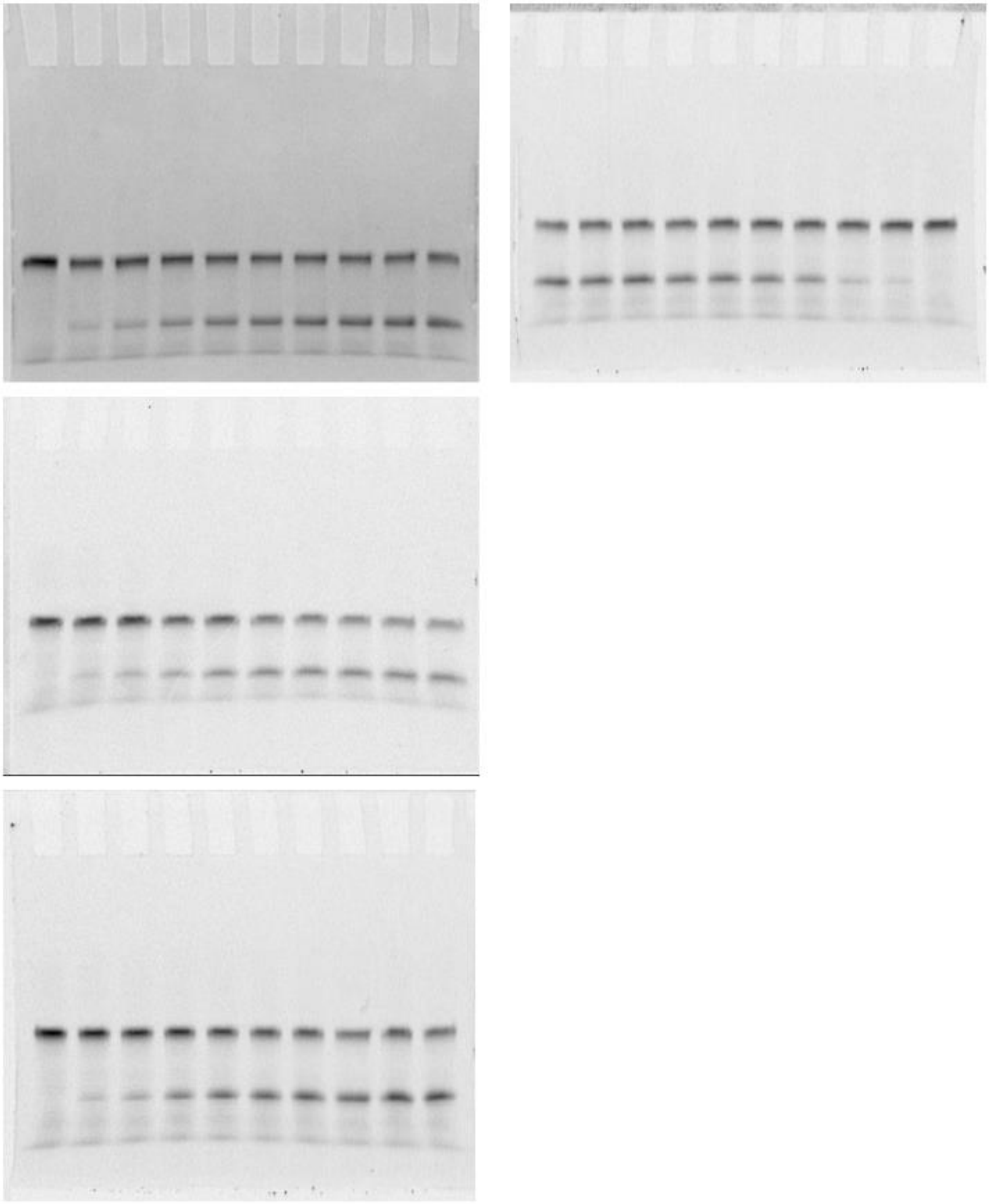

Figure S4. Unedited gel scans of UDG time course with 5FU:A substrate in Fig. 3B.

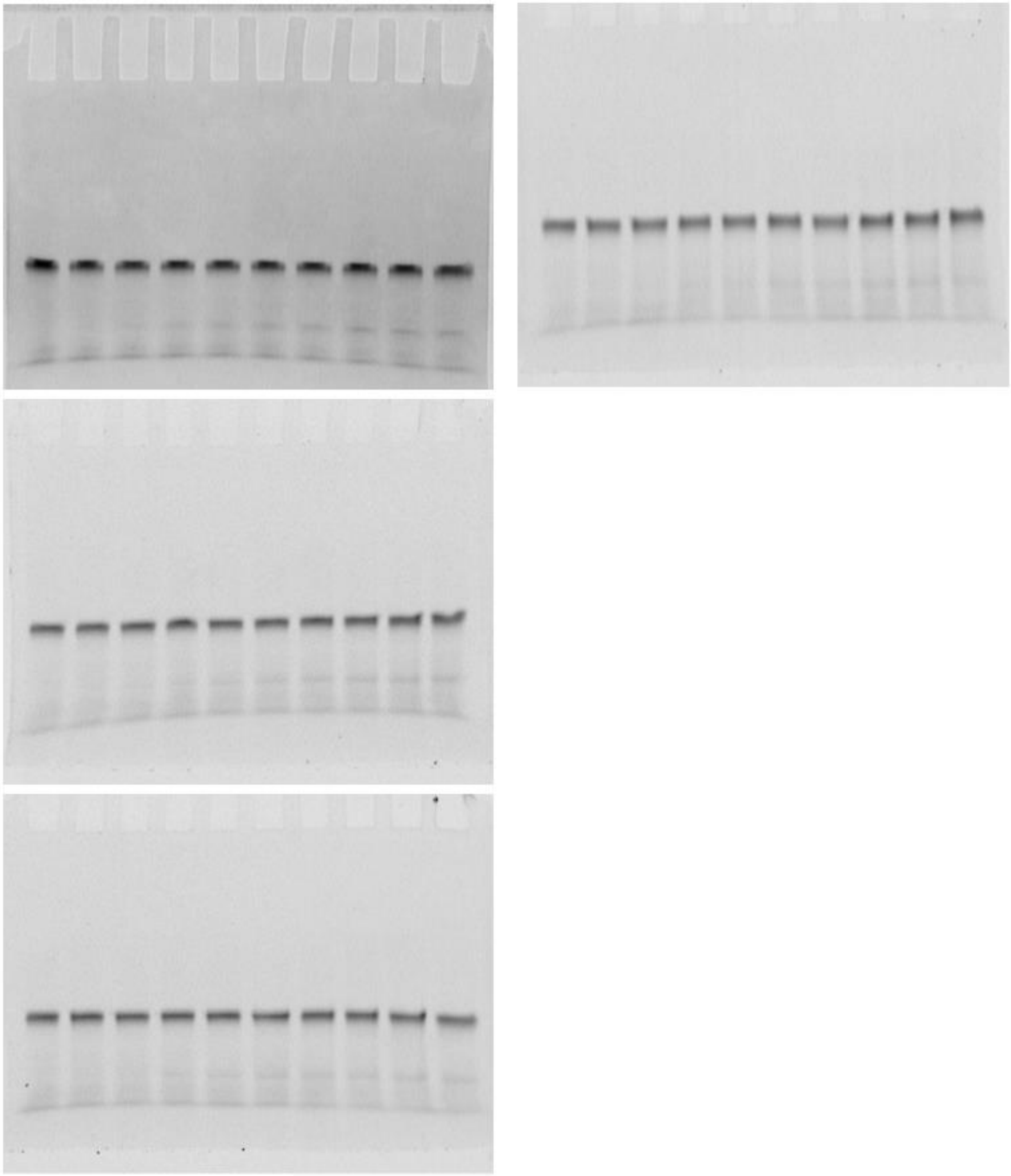

Figure S5. Unedited gel scans of UDG time course with Tel22-5FU substrate in Fig. 3B.

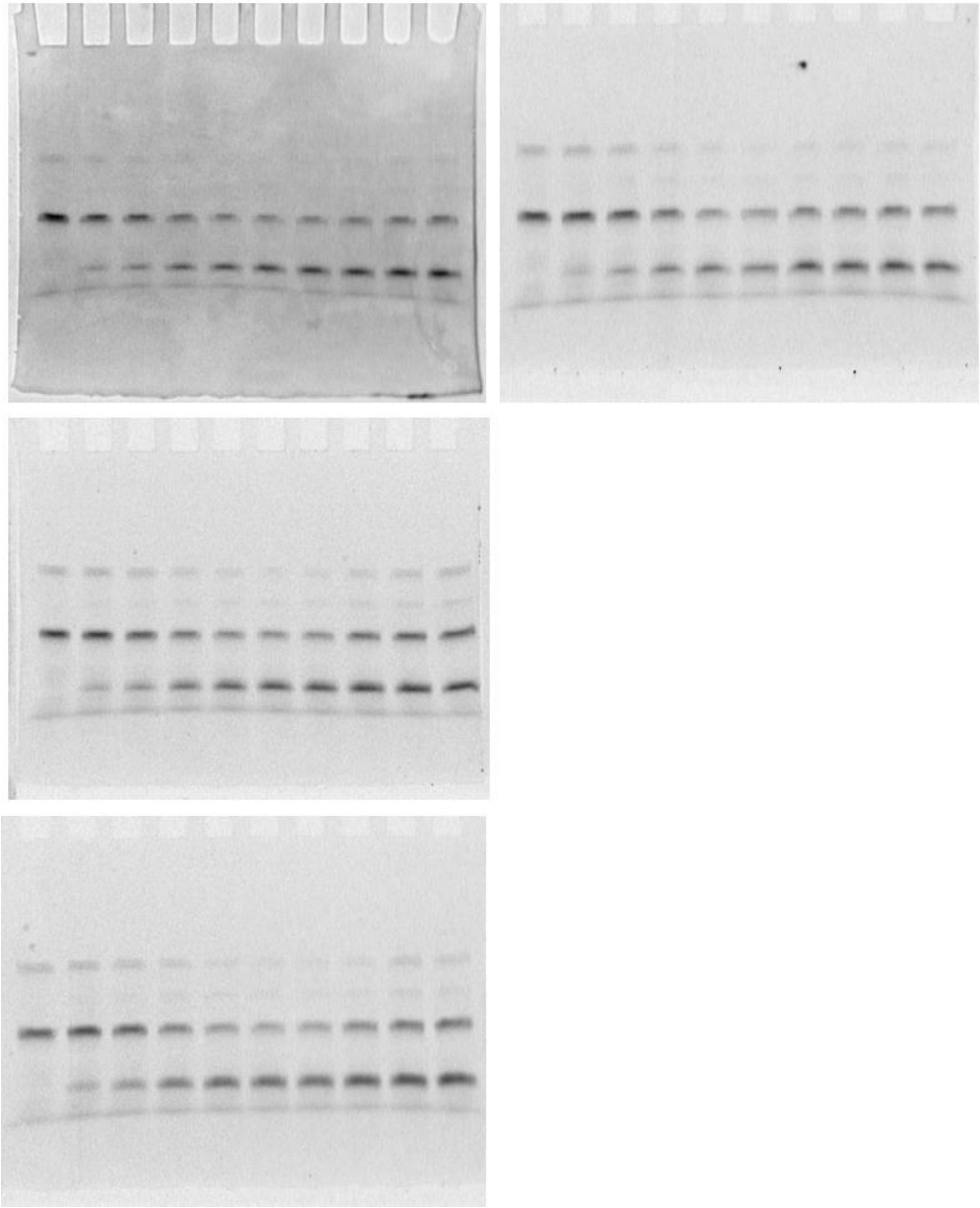

Figure S6. Unedited gel scans of UDG time course with NQ-5FU substrate in Fig. 3B.

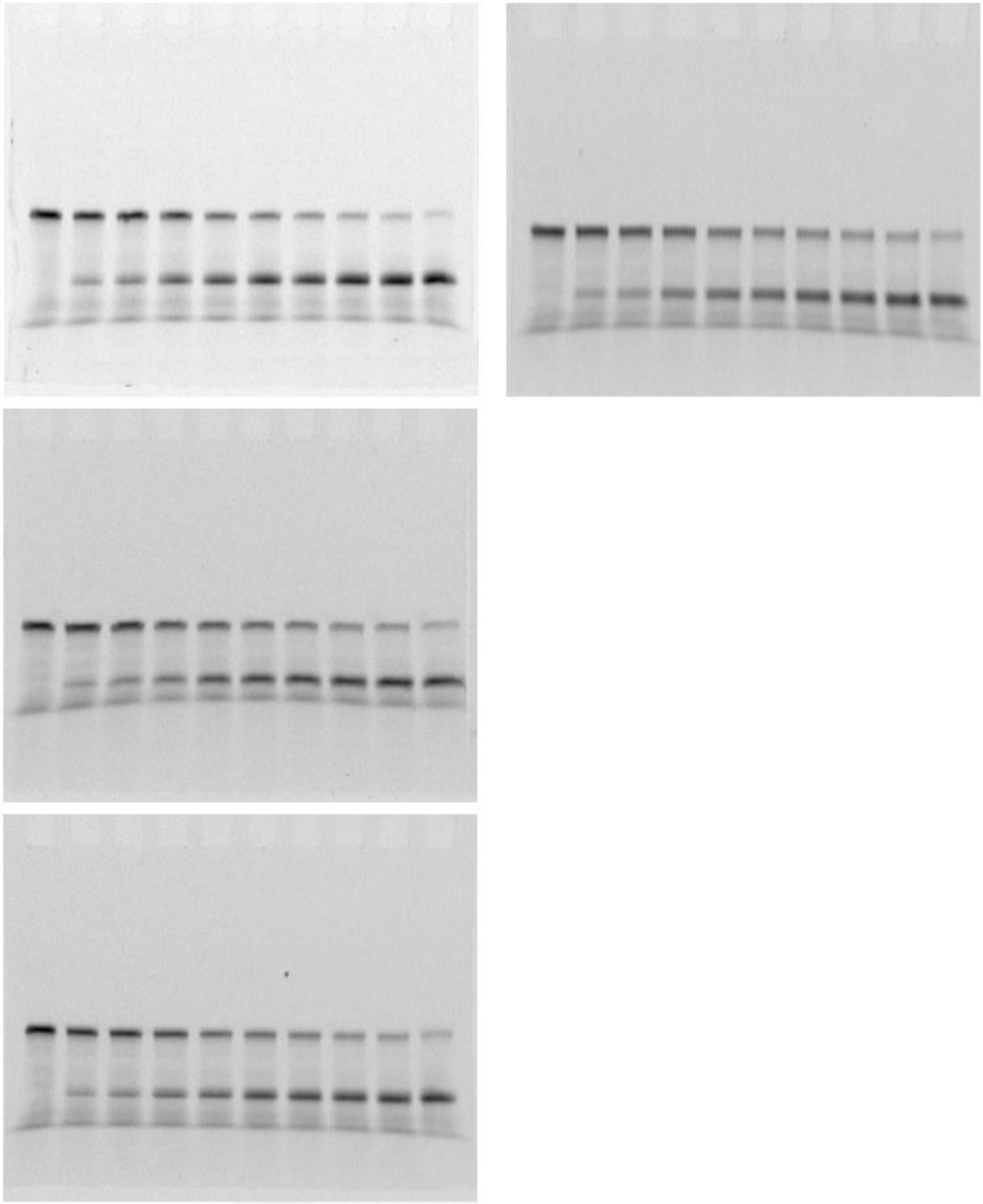

Figure S7. Unedited gel scans of hSMUG1 time course with substrate U:A in Fig. 3C.

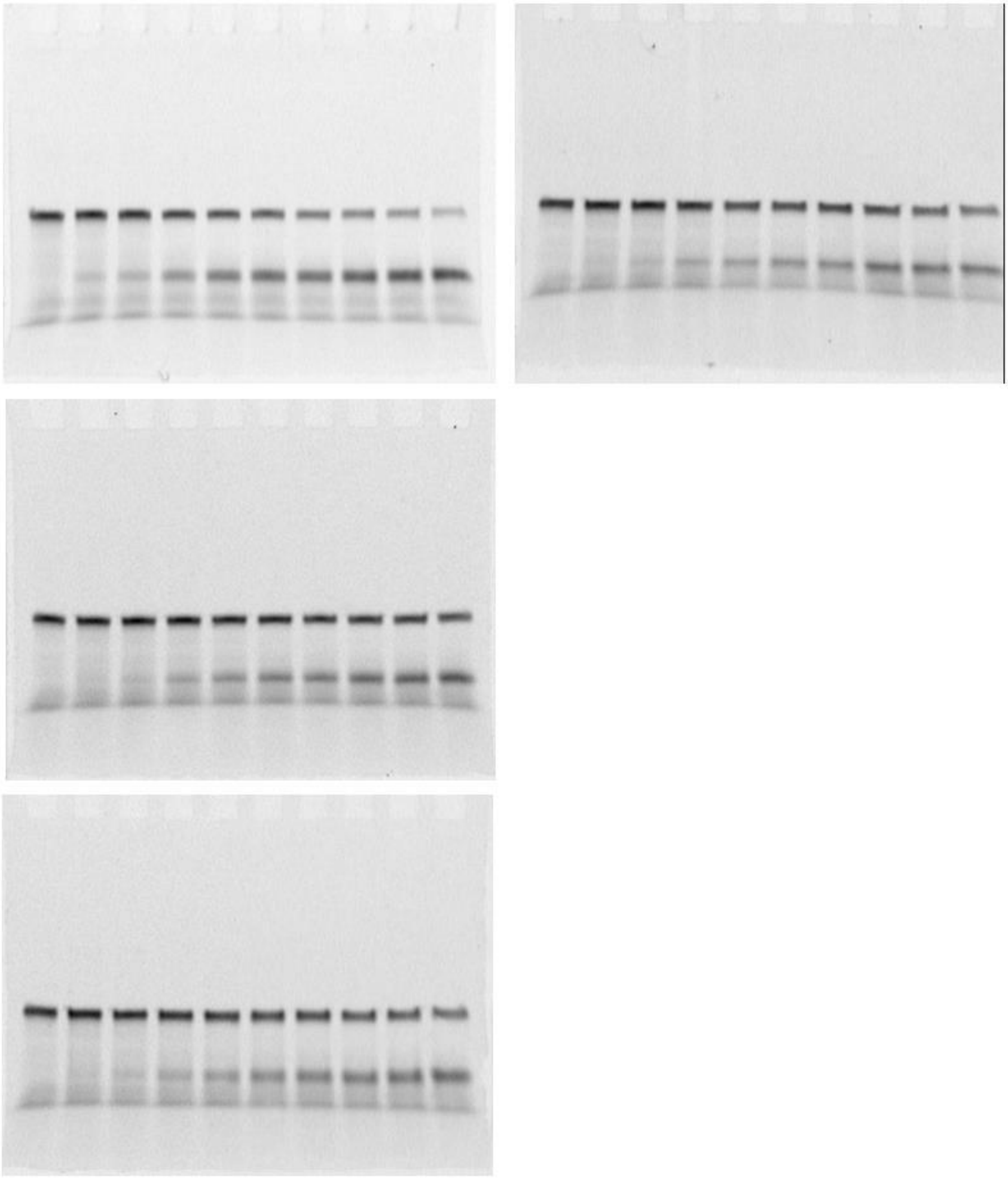

Figure S8. Unedited gel scans of hSMUG1 time course with substrate Tel22-U in Fig. 3C.

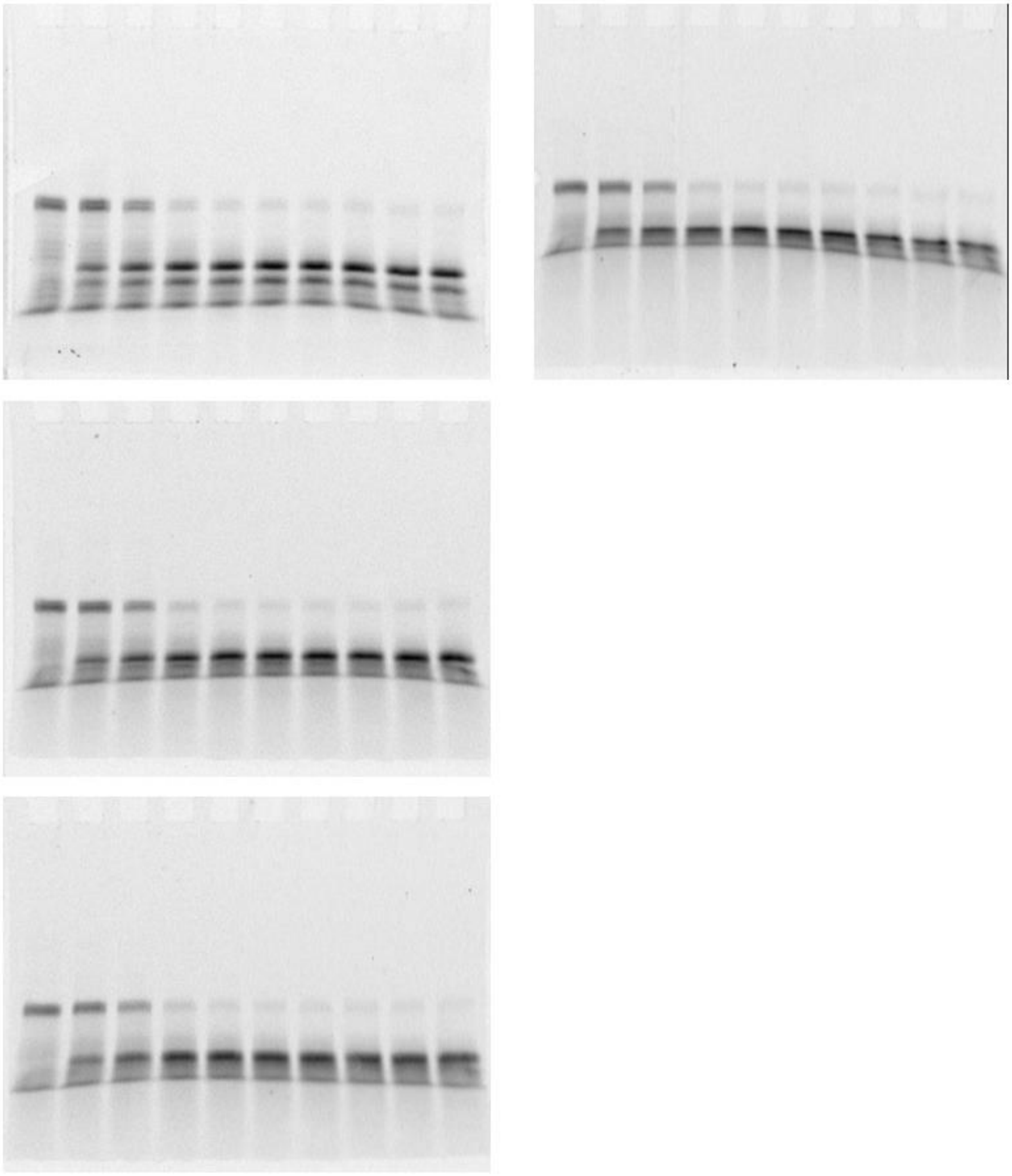

Figure S9. Unedited gel scans of hSMUG1 time course with substrate NQ-U in Fig. 3C.

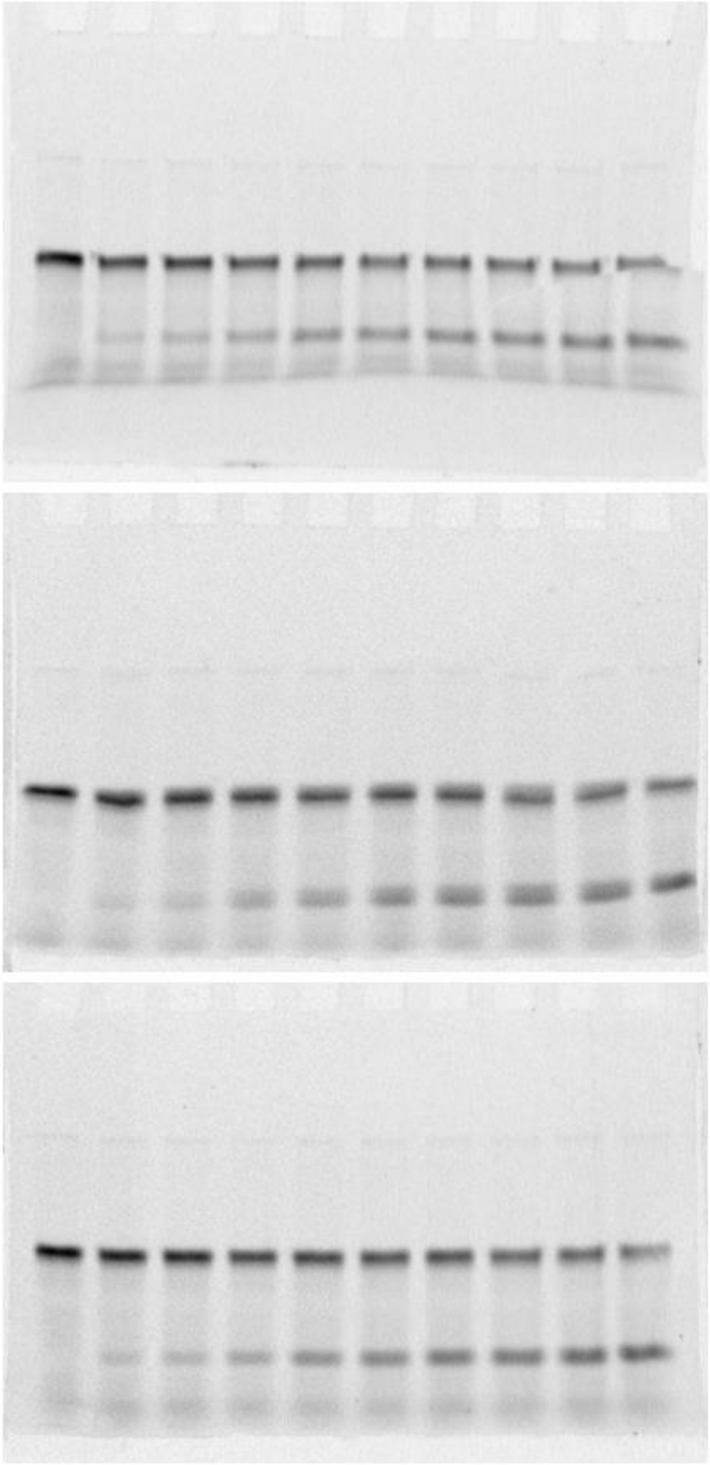

Figure S10. Unedited gel scans of hSMUG1 time course with substrate 5hmU:A in Fig. 3D.

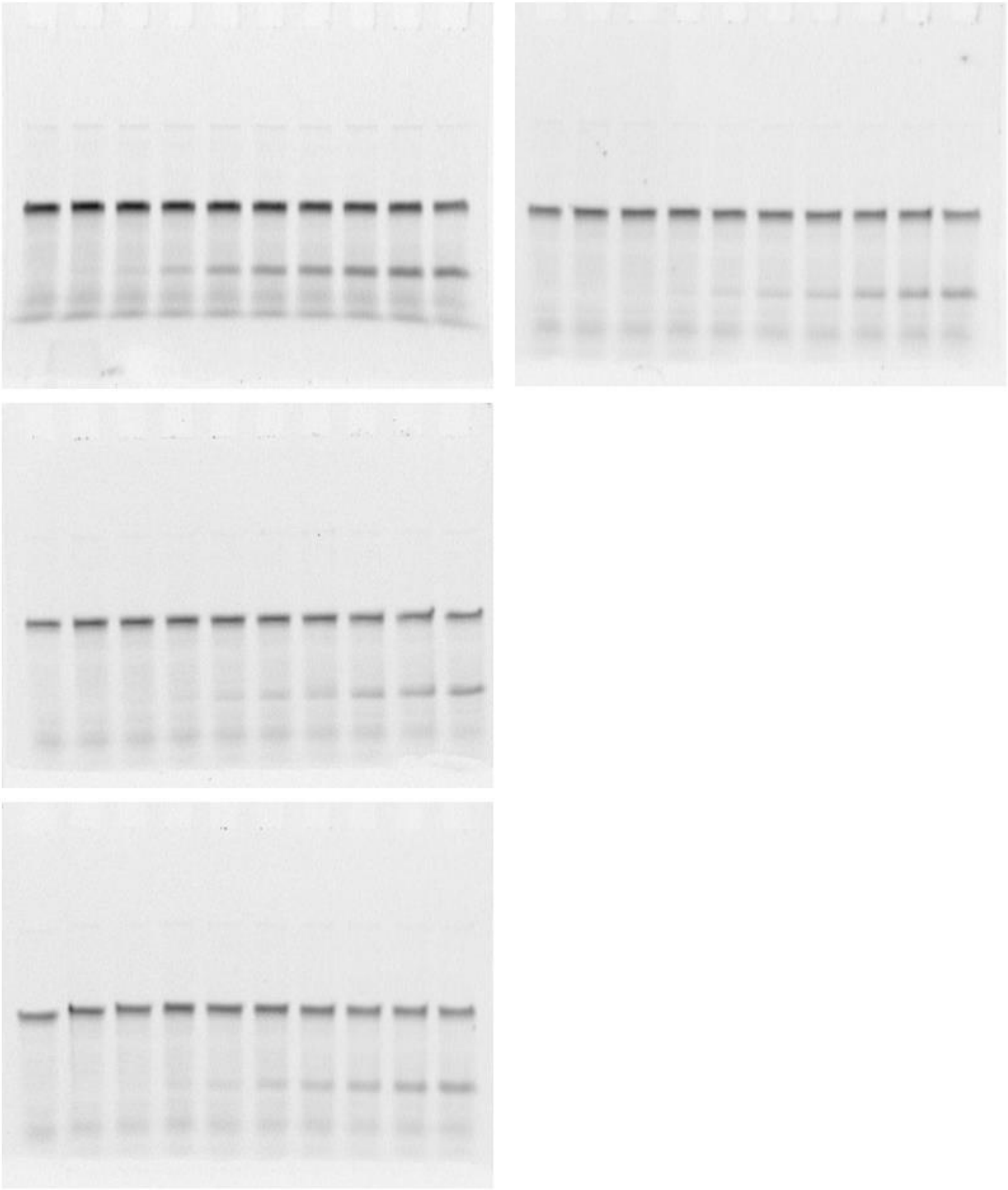

Figure S11. Unedited gel scans of hSMUG1 time course with substrate Tel22-5hmU in Fig. 3D.

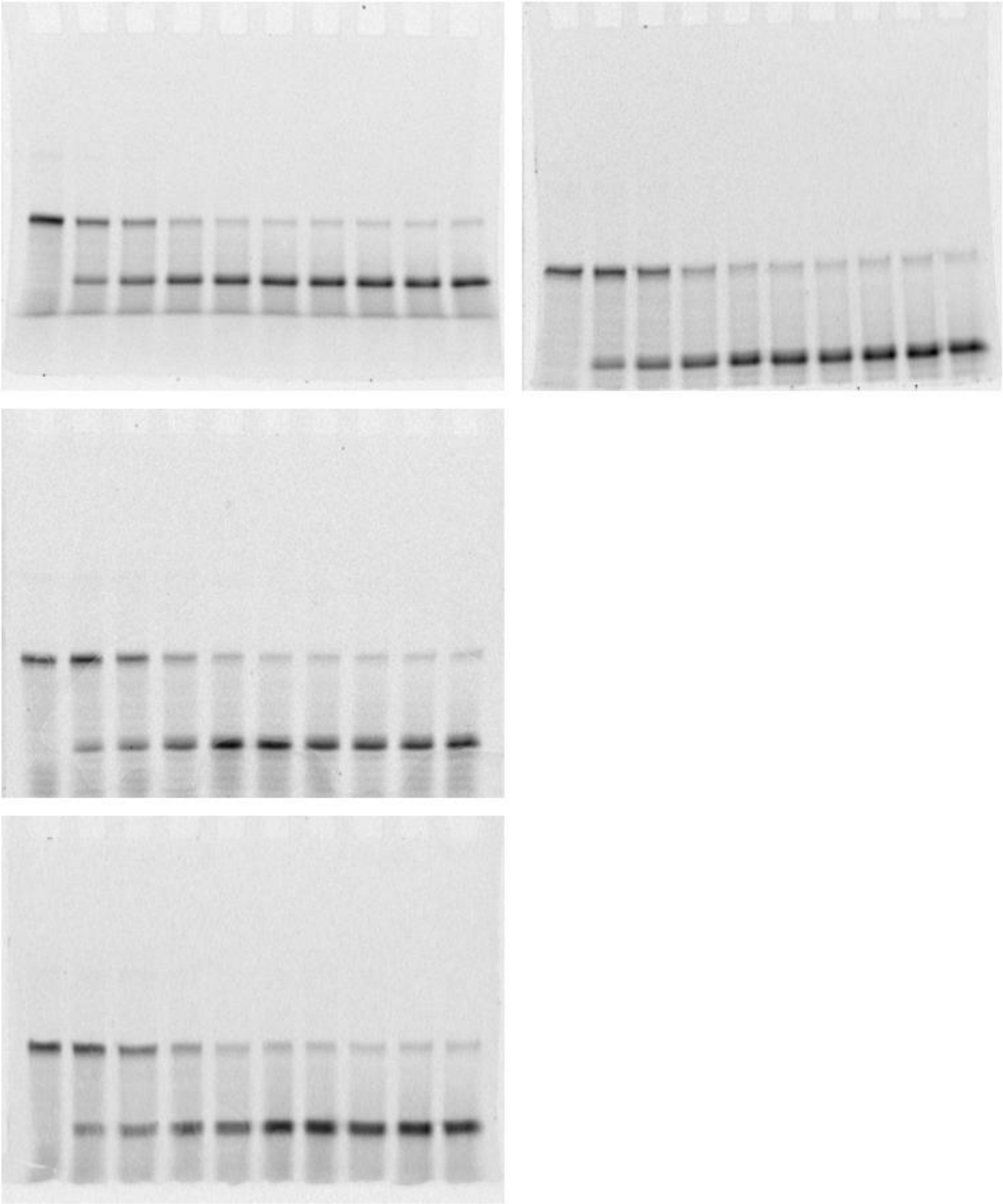

Figure S12. Unedited gel scans of hSMUG1 time course with substrate NQ-5hmU in Fig. 3D.

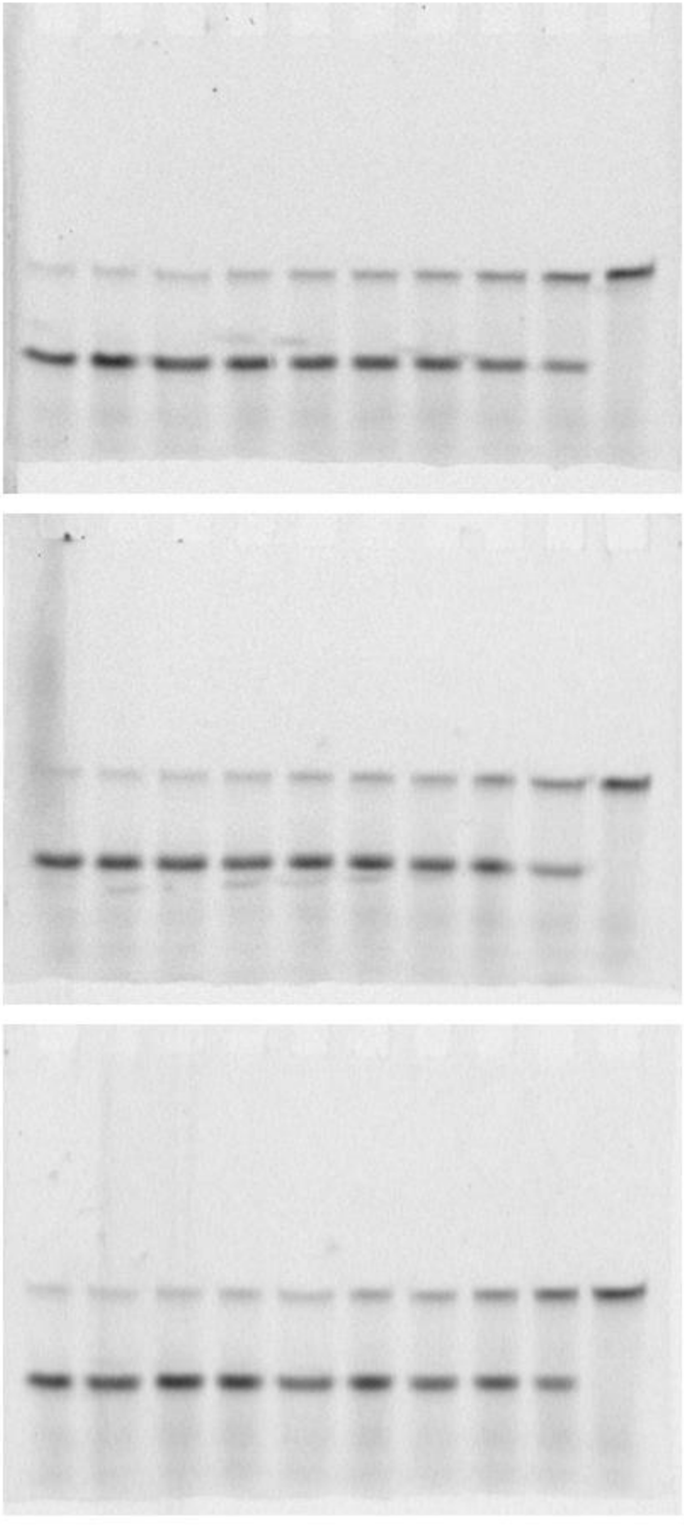

Figure S13. Unedited gel scans of APE1 time course with substrate THF:A in Fig. 3E.

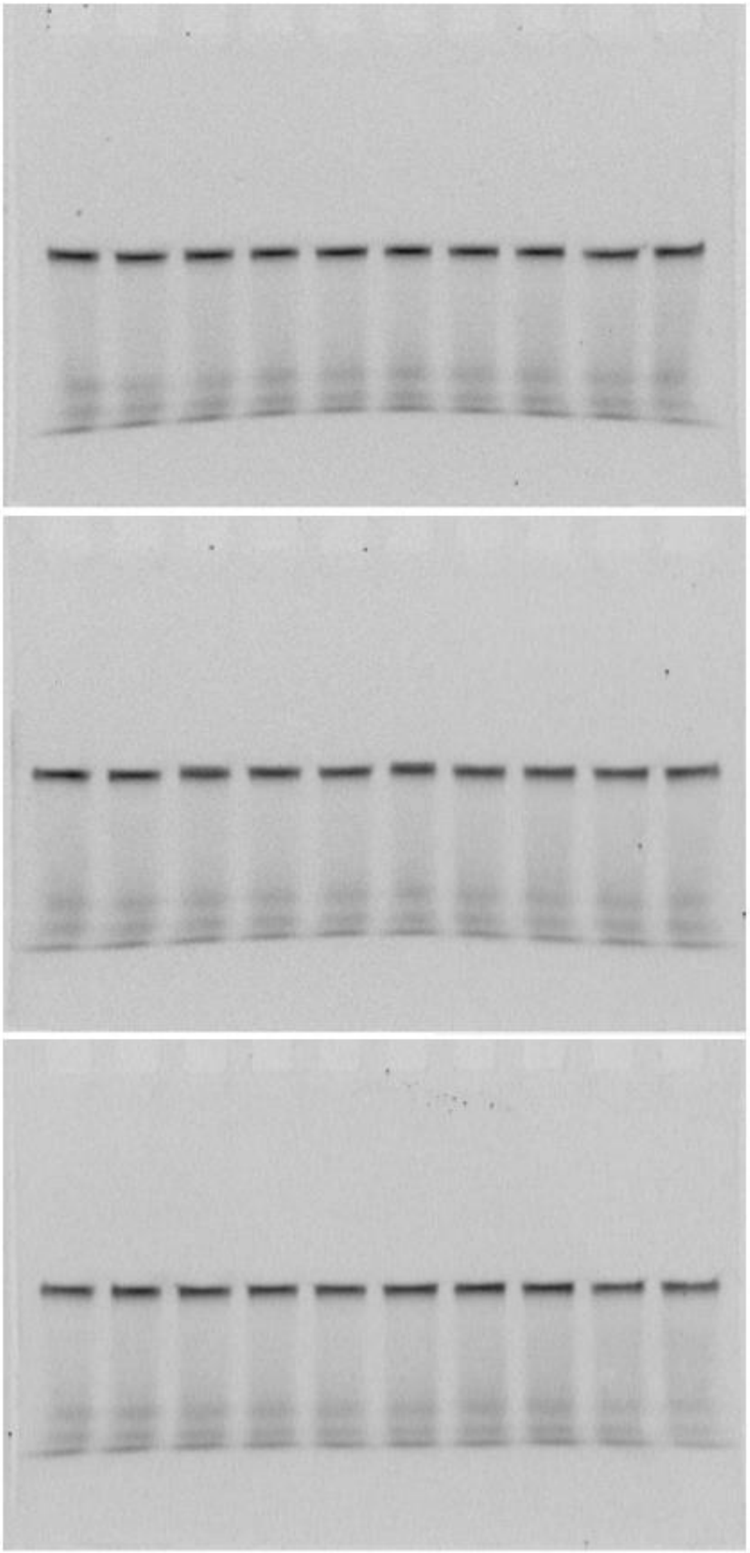

Figure S14. Unedited gel scans of APE1 time course with substrate Tel22-THF in Fig. 3E.

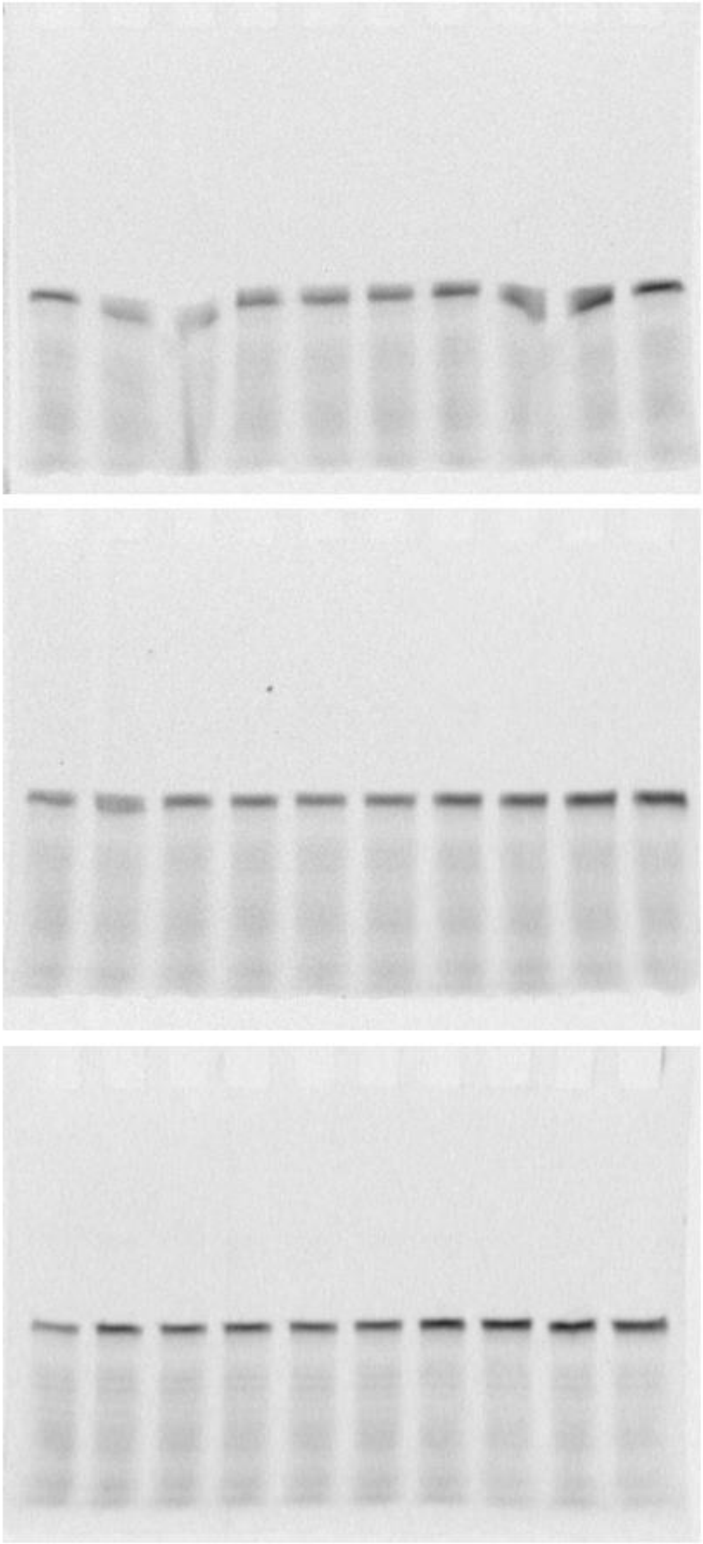

Figure S15. Unedited gel scans of APE1 time course with substrate NQ-THF in Fig. 3E.

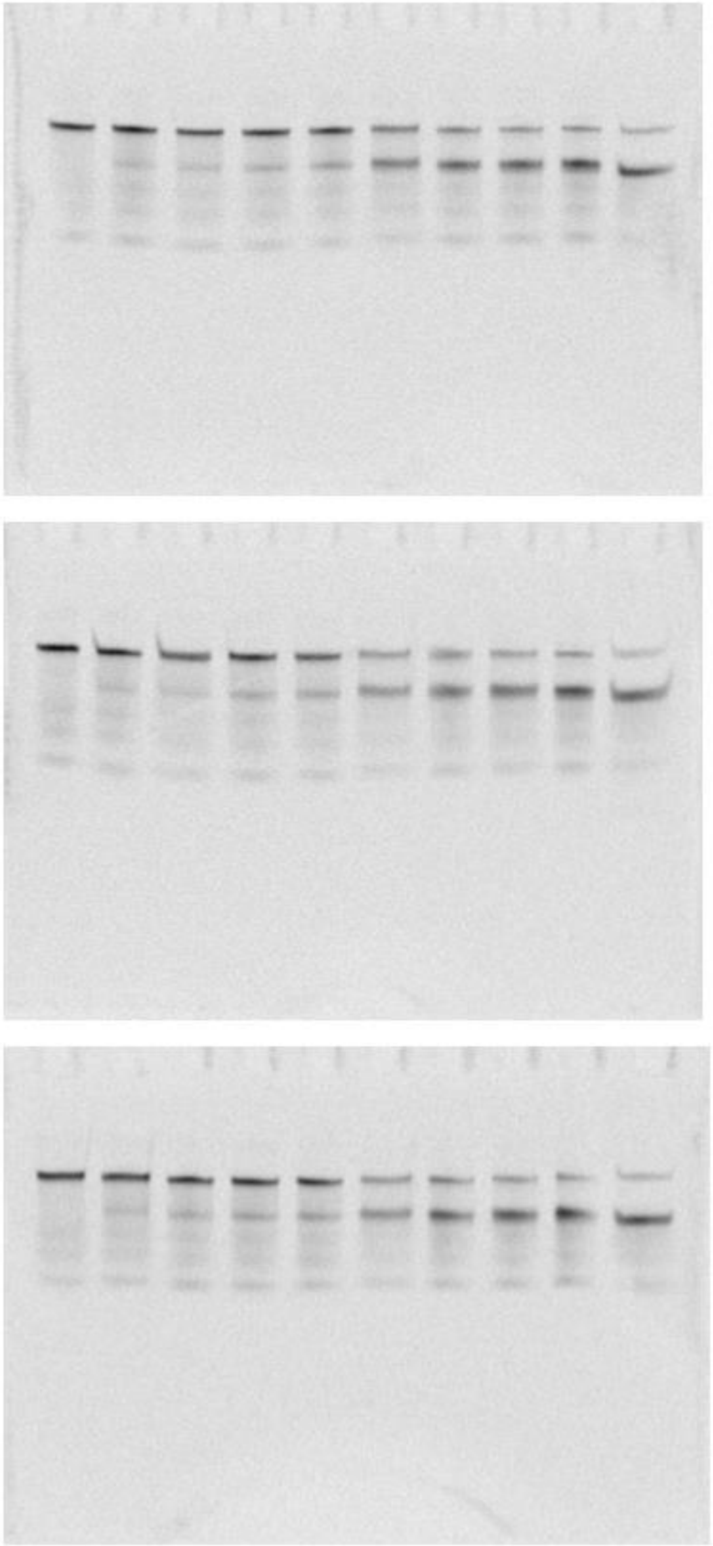

Figure S16. Unedited gel scans of the β-elimination time course with UDG treated substrate Tel22-U in Fig. 3F.

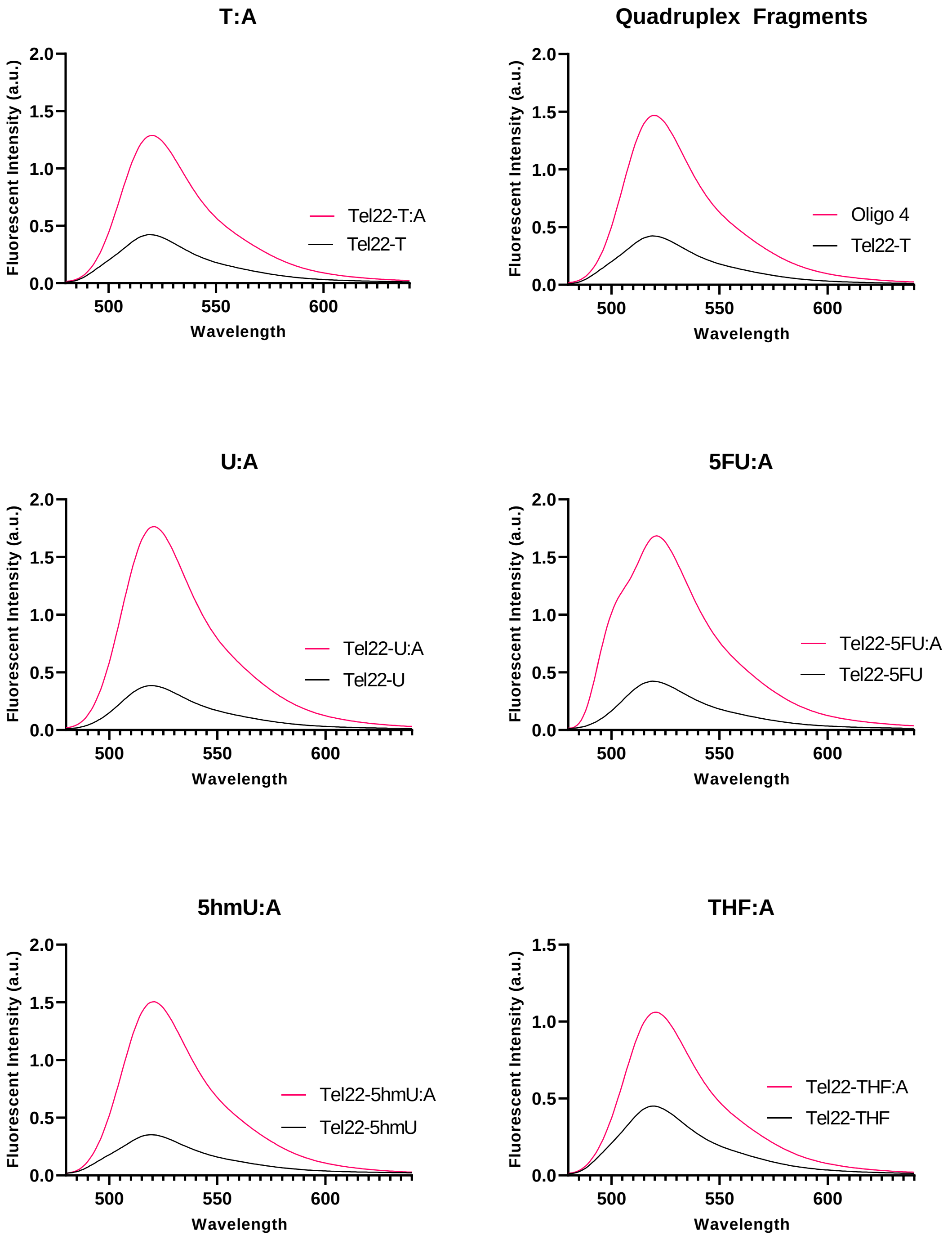

Figure S17. Quadruplexes quench fluorescence compared to duplex. In addition, when the quadruplex oligonucleotide is separated in half (quadruplex fragment), simulating a DNA repair gap, fluorescence is no longer quenched and a similar fluorescence intensity to duplex is observed.

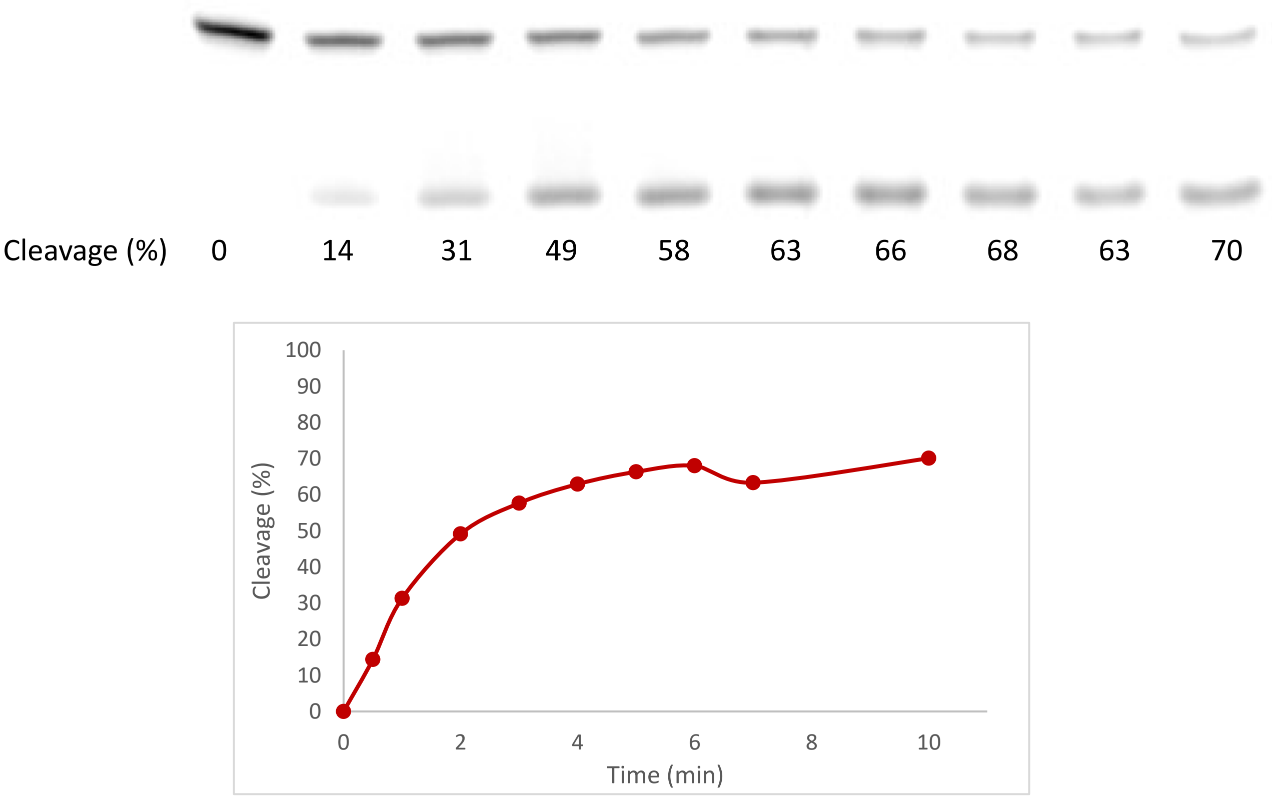

Figure S18. Rate of gap formation in Figure 6 time course. The Tel22-T oligo paired with Oligo 9 (U-Cy5) was purified using an NEB kit and then annealed to a non-fluorescent oligo to make a A:U-Cy5 duplex oligo. 20% excess of the U-Cy5 oligo was used. A 25 µl reaction was prepared in buffer (20 mM Tris, 150 mM KCl, 15 mM NaCl, 10 mM Mg-AC, pH 7.4) and 25 pmol of oligo duplex (1 µM). UDG and APE1 were combined and then an aliquot containing 1.25 U UDG (0.42 pmol, 17 nM) and 2.5 U APE 1 (0.09 pmol, 3.6 nM) was added to the reaction, mixed, and placed into a 37 °C thermocycler. At each time point, the reaction was mixed 2-3X with a pipette and then 2.5 µl (2.5 pmol oligo) was taken and quenched with 12.5 µl of cold formamide and placed on ice. Samples were heated to 95 °C for 1 min and then run on a 6 M urea 20% PAGE gel.
